# Water-Dispersible, Magnetically Recyclable Heterogeneous Cobalt Catalyst for C–C and C–N Cross-Coupling Reactions in Aqueous Media

**DOI:** 10.1021/acsomega.3c10462

**Published:** 2024-07-09

**Authors:** Safoora Sheikh, Aditya Bhattacharyya, Marco A. Henriquez, Mohammad Ali Nasseri, Mohammad Chahkandi, Ali Allahresani, Oliver Reiser

**Affiliations:** † Department of Chemistry, University of Birjand, P.O. Box 97175-615 Birjand, Iran; ‡ Institut für Organische Chemie, Universität Regensburg, Universitätsstraße 31, Regensburg 93053, German; § Department of Chemistry, Hakim Sabzevari University, P.O. Box 96179-76487 Sabzevar, Iran

## Abstract

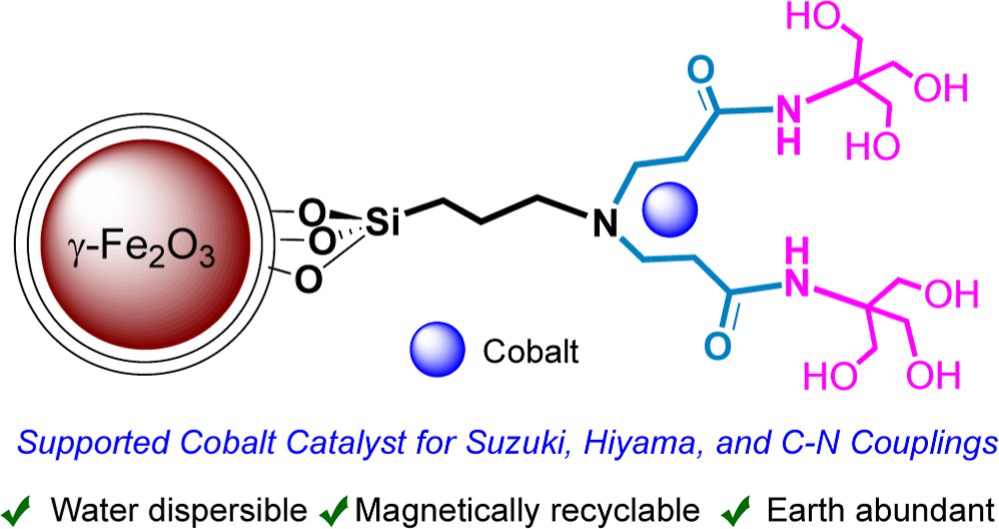

A cobalt catalyst supported on an iron oxide core, denoted as γ-Fe_2_O_3_@PEG@THMAM-Co, has been prepared and characterized by Fourier transform infrared spectroscopy, X-ray diffraction, field emission scanning electron microscopy, transmission electron microscopy, energy-dispersive X-ray mapping, thermogravimetry differential thermogravimetry, vibrating sample magnetometry, and inductively coupled plasma. Polyhydroxy end groups in the shell make the catalyst particles dispersible in water, allowing Hiyama, Suzuki, and C–N cross-coupling reactions of aryl iodides and bromides. The catalyst could be recovered by magnetic decantation and reused for at least five successive runs with a negligent decrease in its activity or changes in its morphology. Water as a solvent without requiring additives, surfactants, or organic co-solvents, as well as an abundant and low-cost cobalt catalyst combined with facile recovery, low leaching, and scalability, provides an environmentally and economically attractive alternative to established palladium-catalyzed C–C and C–N coupling reactions.

## Introduction

Carbon–carbon and carbon–nitrogen bond-forming cross-coupling reactions have attracted significant interest in organic synthesis, medicinal chemistry, and other fields.^1−5^ Consequently, the development of novel methods for their synthesis continues to be an active research area,^6−8^ focusing especially on palladium-based transformations.^9^

Lately, the use of cobalt has emerged as an alternative.^10−12^ In the past decade, a number of Co-catalyzed systems have been reported for C–C and C–N cross-coupling reactions,^13^ such as mTEG-CS-Co-Schiff-base,^14^ cobalt-bisoxazolines,^15^ a mono (NHC) dicobalt complex [(IPr)(Co_2_)Co(μ–η:^2^η^2^-HCCPh)Co(CO)_3_],^16^ graphene oxide-immobilized cobalt Schiff base complexes (CoASGO),^17^ P4VP-CoCl_2_,^18^ or Fe_3_O_4_@PEG/Cu–Co.^19^

Despite the economic benefits, applying cobalt compared with palladium catalysts in cross-coupling reactions is still rare. Besides developing cobalt catalysts with high activity, given the toxicity of this metal, it is equally important to establish systems that are recoverable along with low metal leaching and that can be applied under biocompatible conditions.^20^

One promising approach to achieve the latter is the heterogenization of catalysts by embedding them into a suitable support. Various organic and mineral materials such as zeolites,^21^ polymers,^22^ mesoporous materials,^23^ etc., have been used for this purpose.^24^ Especially, magnetic nanoparticles (MNPs) are attractive supports due to their special physical and chemical properties, holding the promise to combine the best of both worlds, i.e., homogeneous and heterogeneous catalysis.^25,26^ γ-Fe_2_O_3_ MNPs are arguably more suitable platforms than other MNPs due to their high resistance against oxidation and their high saturation magnetism while being paramagnetic and thus having a low tendency for agglomeration in the absence of a magnetic field.^27,28^ On the other hand, from the perspective of sustainable chemistry and development, the use of green solvents in chemical reactions is highly desirable.^29^ Water has unique advantages compared to conventional organic solvents as it is widely available and abundant, economical and safe, nonvolatile, nonflammable, nontoxic, and non-carcinogenic. However, the performance and selectivity of catalysts supported on MNPs are not high in water, especially when the substrates employed for a reaction have low solubility in the latter.^30^ Therefore, developing water-dispersible MNPs while maintaining their heterogeneous character for effective recovery is desirable.

**Scheme 1:**
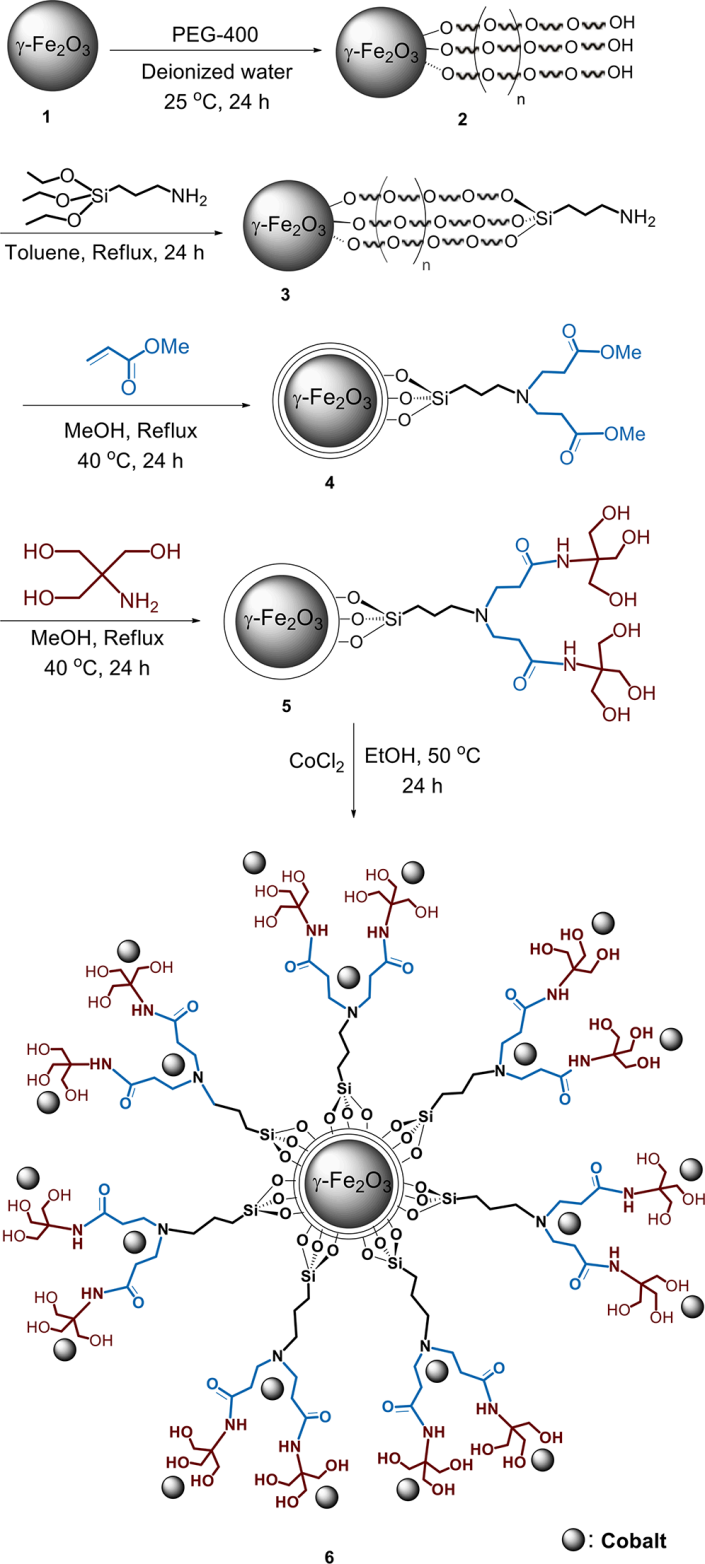
Preparation of γ-Fe_2_O_3_@PEG@THMAM-Co **6**

Aiming to design a magnetic platform that is water-dispersible, we set out to combine readily available iron oxide MNPs as the cores decorated with a hydrophilic coating that would allow the facile incorporation of catalytically active cobalt nanoparticles. As part of the continued efforts of our research groups to promote green and water-dispersible catalysts,^31,32^ we report here a water-dispersible and magnetically recyclable cobalt catalyst using γ-Fe_2_O_3_ MNPs modified by PEG (polyethylene glycol) as a hydrophilic platform (Scheme 1) for cross-coupling reactions, including, Suzuki, Hiyama, and C–N couplings in water.

## Experimental Section

### Preparation of γ-Fe_2_O_3_@APTES@MAc MNPs **4**

γ-Fe_2_O_3_ MNPs **1** were synthesized by coprecipitation and further converted to γ-Fe_2_O_3_@PEG-400 MNPs **2** following protocols described in the literature.^33^ γ-Fe_2_O_3_@PEG@APTES@Mac **4** nanoparticles were synthesized according to our previous report^31^ (for experimental details, see the Supporting Information).

### Preparation of γ-Fe_2_O_3_@PEG@APTES@MAc@THMAM **5**

**4** (1.0 g) was sonicated in methanol (40 mL) for 30 min, followed by addition of tris(hydroxymethyl)aminomethane (THMAM, 2.5 mmol, 0.3 g) under N_2_ at 25 °C. The mixture was subsequenlty heated at 40 °C for 24 h. The solid product was separated by an external magnet, washed with ethanol three times, and dried at 50 °C overnight to obtain **5** (1.1 g).

### Preparation of γ-Fe_2_O_3_@PEG@APTES@MAc@THMAM-Co **6**

**5** (1.0 g) was sonicated in ethanol (30 mL) for 30 min, followed by the addition of a solution of CoCl_2_·6H_2_O (0.237 g, 1 mmol) in ethanol (20 mL) under N_2_ at 25 °C. The temperature was increased to 50 °C for 24 h. The solid was separated by an external magnet, washed with ethanol three times, and dried in vacuo at 50 °C overnight to yield γ-Fe_2_O_3_@PEG@THMAM-Co **6** (1.0 g). Inductively coupled plasma optical emission spectrometry (ICP-OES) analysis showed 95% incorporation of Co into **6** (0.95 mmol/g).

### General Procedure for the Suzuki Cross-Coupling Reaction (**9a–9q**)

**6** (0.6 mol %, 0.007 g) was added to a mixture of aryl halide (1.0 mmol), phenylboronic acid (1.2 mmol), and K_2_CO_3_ (2.0 mmol) in water (4 mL) under stirring and heated for the indicated time at 80 °C, monitoring the progress of the reaction by thin layer chromatography (TLC) until completion. The nanocatalyst was separated by an external magnet and washed with ethyl acetate (3 × 3 mL). The aqueous phase was extracted with ethyl acetate (3 × 5 mL), and the combined organic layers were dried over anhydrous Na_2_SO_4_. After evaporation of the solvent under reduced pressure, the crude product was purified by column chromatography on silica (eluent: *n*-hexane/EtOAc 8:2) to obtain products **9a–9q**.

### General Procedure for the Hiyama Cross-Coupling Reaction (**11a–11m**)

**6** (0.6 mol %, 0.007 g) was added to a mixture of aryl halide (1.0 mmol), NaOH (2.0 mmol), and triethoxyphenylsilane (1.5 mmol) in water (4 mL) under stirring and heated for the indicated time at 80 °C, monitoring the progress of the reaction by TLC until completion. The nanocatalyst was separated by an external magnet and washed with ethyl acetate (3 × 3 mL). The aqueous phase was extracted with ethyl acetate (3 × 5 mL), and the combined organic layers were dried over anhydrous Na_2_SO_4_. After evaporation of the solvent under reduced pressure, the crude product was purified by column chromatography on silica (eluent: *n*-hexane/EtOAc. 8:2) to obtain products **11a**–**11m**.

### General Procedure for the C–N Coupling Cross-Coupling Reaction (**13a–13h**)

**6** (3 mol %, 0.035 g) was added to a mixture of phenylboronic acid (1.0 mmol), amine (1.5 mmol), and NaOH (2.0 mmol) in water (4 mL) under stirring and heated for the indicated time at 100 °C, monitoring the progress of the reaction by TLC until completion. The nanocatalyst was separated by an external magnet and washed with ethyl acetate (3 × 3 mL). The aqueous phase was diluted by water (5 mL) and extracted with ethyl acetate (3 × 10 mL). The combined organic layers were dried in anhydrous Na_2_SO_4_. After evaporation of the solvent under reduced pressure, the crude product was purified by column chromatography on silica (eluent: *n*-hexane/EtOAc, 7/3) to obtain the C–N coupling products **13a**–**13h**.

## Results and Discussion

**Figure 1:**
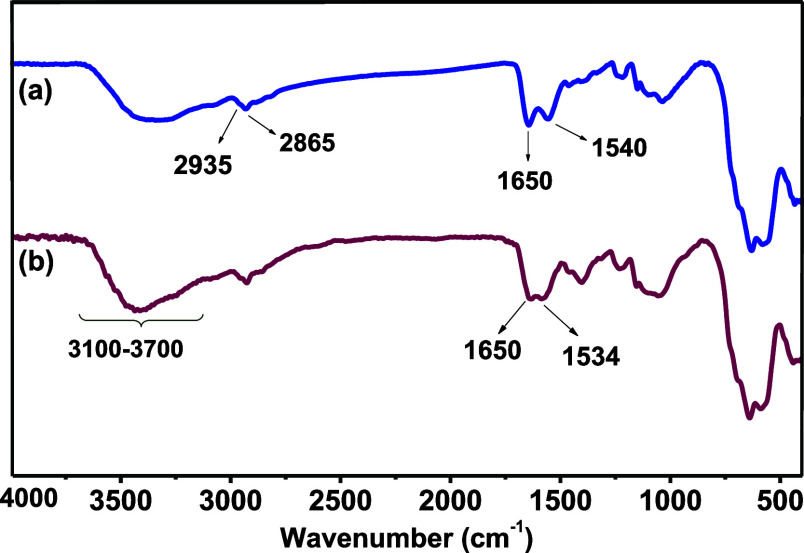
FT–IR spectra of (a) γ-Fe_2_O_3_@PEG@APTES@MAc@THMAM **5** and (b) γ-Fe_2_O_3_@PEG@ @THMAM-Co **6**.

We began our investigation by preparing γ-Fe_2_O_3_@PEG@THMAM-Co **6** (Scheme 1) based on our reported procedure for the synthesis of **4**,^31^ grafting tris(hydroxymethyl)aminomethane (THMAM) through an amino-siloxy and methyl acrylate linker onto the surface of γ-Fe_2_O_3_ core–shelled with PEG-400 **2**, followed by impregnation with cobalt (Scheme 1). Fourier transform infrared spectroscopy (FT-IR) proved to be a convenient method to follow the synthetic sequence of **6** (Figure S1a–d). The successful attachment of THMAM onto **4** to yield **5** is confirmed by the appearance of the stretching vibrations for the carbonyl moiety of the amide group (−CONH−) around 1650 cm^–1^,^34^ while the absorption band around 1540 cm^–1^ is attributed to the bending mode of N–H (Figure 1a),^35^ which slightly shifts (1534 cm^–1^) upon impregnation with cobalt in **6**, indicating Co–N coordination. The broad bands between 3100–3700 cm^–1^ correspond to −NH and −OH stretching vibrations (Figure 1a,b). The peak at 1485 cm^–1^ corresponds to the bending of the (−CH_2_−) bonds, whereas the peak around 1220 cm^–1^ is ascribed to the stretching vibrations of (C–N) bonds (Figure S1b–d).^36^

**Figure 2:**
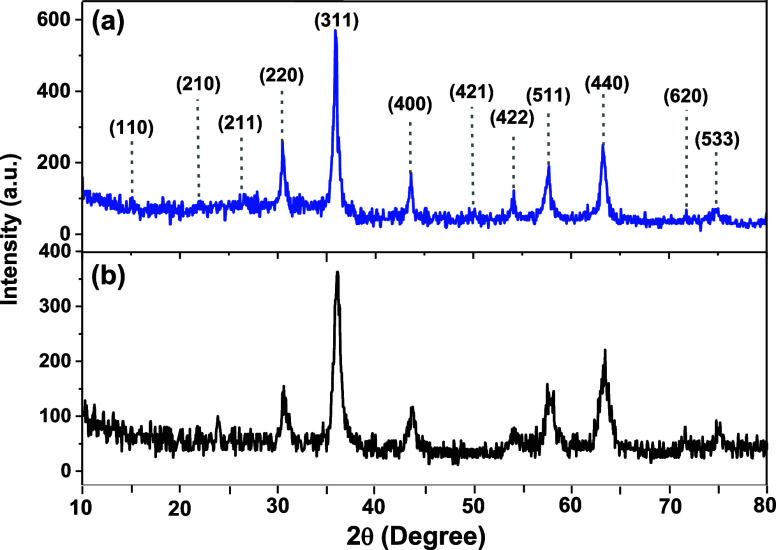
XRD patterns of (a) γ-Fe_2_O_3_**1** adapted with permission from ref (40); Copyright [2020, Elsevier] and (b) γ-Fe_2_O_3_@PEG@THMAM-Co **6**.

The X-ray diffraction (XRD) patterns of γ-Fe_2_O_3_**1** and γ-Fe_2_O_3_@PEG@THMAM-Co **6** (Figure 2) suggest in both cases a cubic magnetite crystal structure, in agreement with recorded database peaks (JCPDS card no. 39-1346). The diffractogram of **1** indicates several diffraction main peaks at 15, 23.1, 26.9, 30.3, 35.8, 43.6, 50, 54.8, 57.3, 63.2, 71.1, and 74.4° (2θ), corresponding to the (110), (210), (211), (220), (311), (400), (421), (422), (511), (440), (620), and (533) Miller indices, respectively (Figure 2a),^37,38^ being also present in the XRD pattern of **6** at a decreased peak intensity, reflecting the functionalization of maghemite NPs (Figure 2b). The crystallite size of **6** was determined to be 11.4 nm (114 Å) (Figure S1) by using the Williamson–Hall equation βcosθ = (*K*λ)/*D* + ηsinθ^39^ assuming 0% strain.

**Figure 3:**
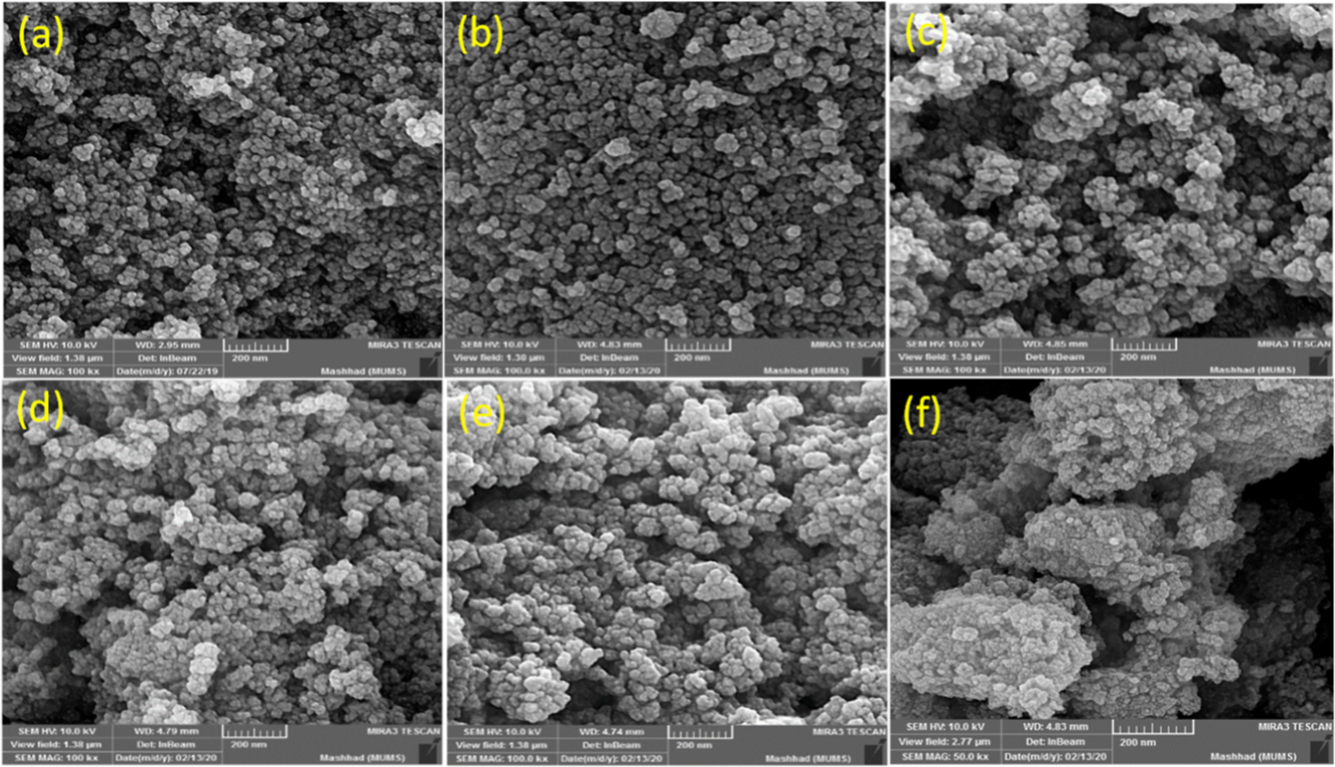
Field emission SEM pictures of (a) γ-Fe_2_O_3_**1**, (b) γ-Fe_2_O_3_@PEG **2**, (c) γ-Fe_2_O_3_@PEG@APTES **3**, (d) γ-Fe_2_O_3_@PEG@APTES@MAc **4**, (e) γ-Fe_2_O_3_@PEG@APTES@MAc@THMAM **5**, and (f) γ-Fe_2_O_3_@PEG@THMAM-Co **6**. **1**–**4** were prepared according to ref (31).

Scanning electron microscopy (SEM) investigations were also performed to study the surface and cross-sectional morphology during the preparation of **6** (Figure 3a–f), showing homogeneity, spherical morphology, and uniform size distribution of the materials in all of the steps.

**Figure 4:**
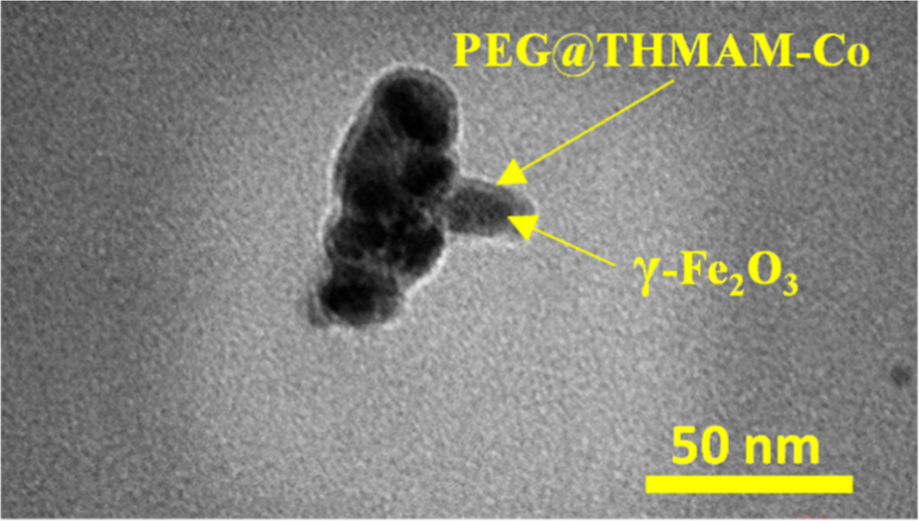
TEM picture of γ-Fe_2_O_3_@PEG@THMAM-Co **6**.

In addition, the morphology of **6** was analyzed by transmission electron microscopy (TEM) (Figure 4), showing a small, spherical structure without significant agglomeration.

**Figure 5:**
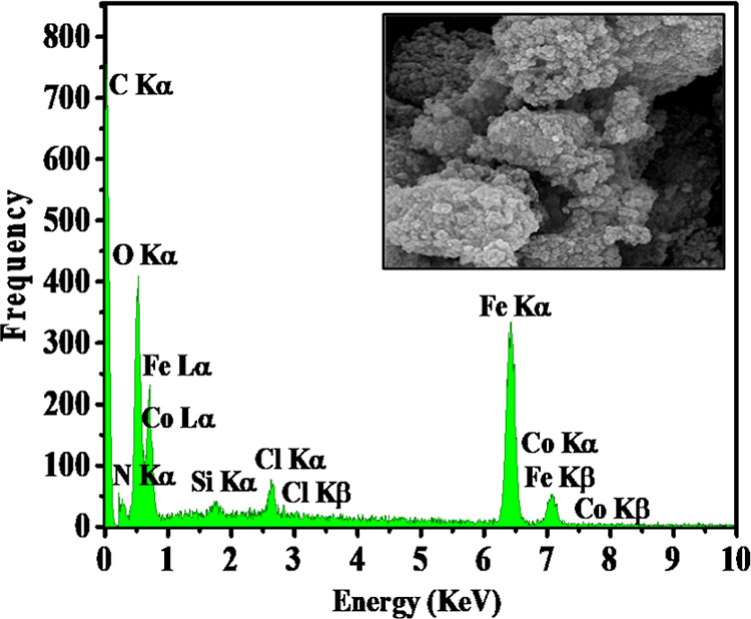
EDX analysis of γ-Fe_2_O_3_@PEG@THMAM-Co **6**.

**Figure 6:**
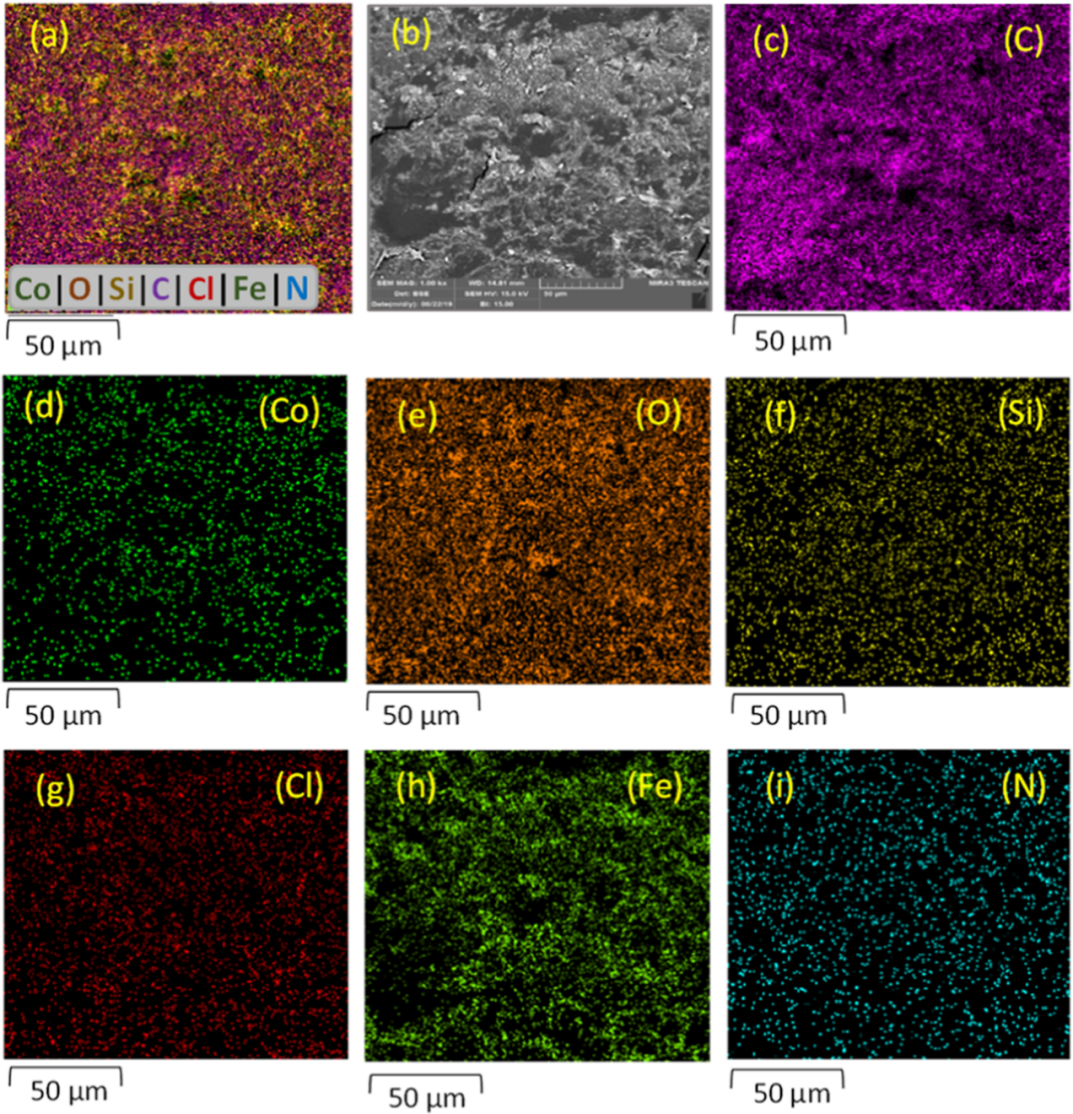
EDX elemental mappings of γ-Fe_2_O_3_@PEG@THMAM-Co **6**. (a) Overlay; (b) structural morphology related to the mapping imaging area of γ-Fe_2_O_3_@PEG@THMAM-Co **6**; (c) carbon; (d) cobalt; (e) oxygen; (f) silicon; (g) chlorine; (h) iron; and (i) nitrogen.

Energy-dispersive X-ray (EDX) analysis confirmed that **6** contains Co, N, C, O, Si, Cl, and Fe (Figure 5). EDX elemental mapping also shows all expected elements, including Fe, N, C, Si, Co, O, and Cl but, moreover, proved that Co is homogeneously distributed on the γ-Fe_2_O_3_@PEG platform (Figure 6), which is especially relevant for high catalytic activity. Important for the subsequent catalytic cross-coupling reactions, ICP-OES analysis showed no presence of copper, nickel, or palladium within the detection limit (<10 ppm).

**Figure 7:**
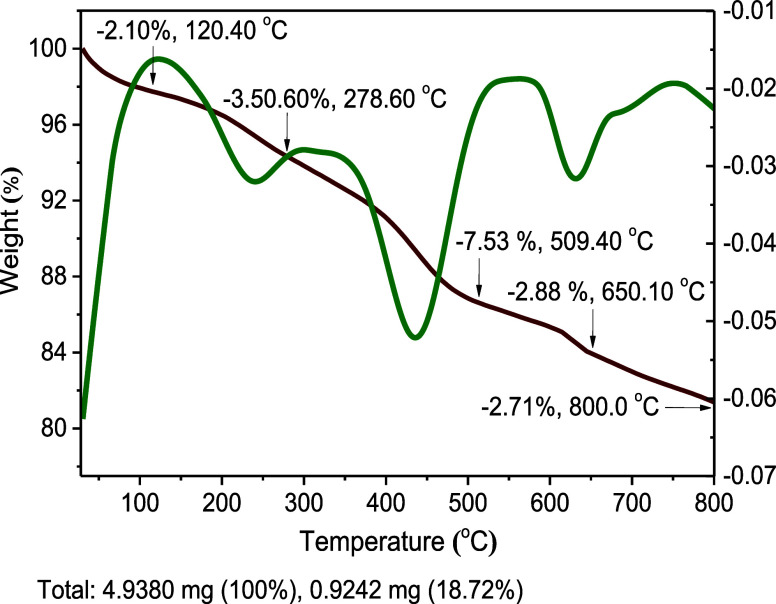
TGA differential thermogravimetry weight loss curves of γ-Fe_2_O_3_@PEG@THMAM-Co **6**.

Thermogravimetry analysis (TGA) of **6** at a heating rate of 10 °C min^–1^ in the temperature range of 25–800 °C under a nitrogen atmosphere (Figure 7) showed weight loss at five stages totaling 18.7%, giving credit to the high thermal stability of γ-Fe_2_O_3_@PEG@THMAM-Co **6**. The initially trapped water in the crystalline structure of the Co complex **6** is likely to be removed (2.1 W % loss, ∼120 °C), followed by decomposition of the organic linker materials grafted onto the surface of γ-Fe_2_O_3_ in the regions of 278 and 509 °C (3.5 and 7.5 W % loss) (Figure 7). At even higher temperatures (650 and 800 °C), the observed weight loss (2.9 and 2.7 W %) is most likely related to the removal of PEG as well as of mineral impurities in γ-Fe_2_O_3_**1** (Figure 7).

**Figure 8:**
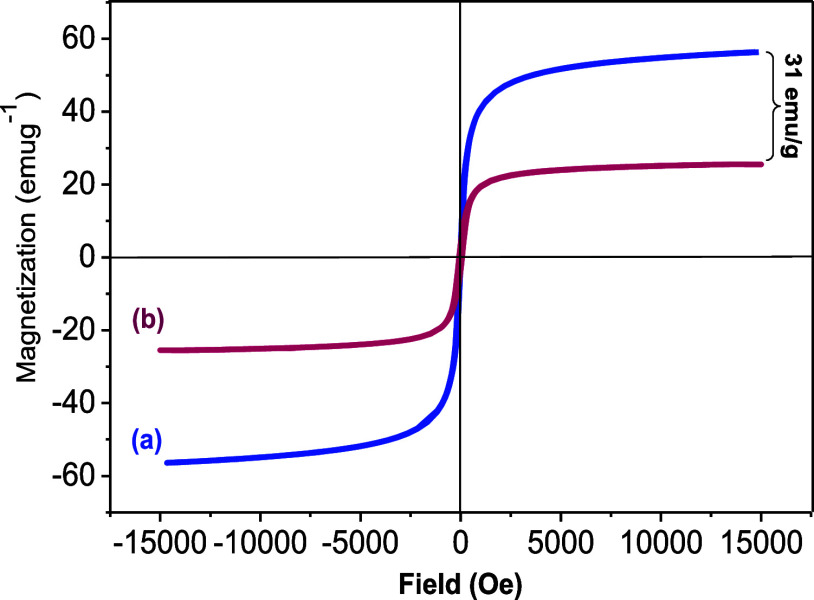
Magnetization curves of (a) γ-Fe_2_O_3_**1** and (b) γ-Fe_2_O_3_@PEG@THMAM-Co **6** at 300 K.

The magnetic properties of γ-Fe_2_O_3_ MNPs **1** and γ-Fe_2_O_3_@PEG@THMAM-Co **6** were evaluated by using vibrating sample magnetometry analysis (VSM) at 25 °C (Figure 8). According to the VSM results, the magnetization for γ-Fe_2_O_3_ MNPs was found to be 55 emu g^–1^, which dropped to 31 emu g^–1^ for γ-Fe_2_O_3_@PEG@THMAM-Co **6** (Figure 8), being in line with the successful functionalization of γ-Fe_2_O_3_ MNPs **1**. Finally, a loading of 0.95 mmol of Co/g of **6** was determined by ICP-OES analysis.

### Evaluation of the Catalytic Activity of γ-Fe_2_O_3_@PEG@THMAM-Co MNPs **6**

**Figure 9:**
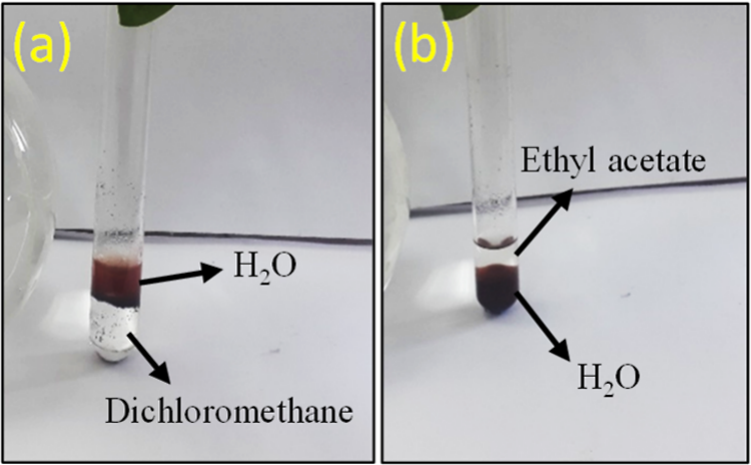
(A) Distribution of γ-Fe_2_O_3_@PEG@THMAM-Co **6** in biphasic H_2_O–dichloromethane (1:1). (B) Distribution of γ-Fe_2_O_3_@PEG@THMAM-Co **6** in biphasic H_2_O–ethyl acetate (1:1).

Given the hydrophilic properties of γ-Fe_2_O_3_@PEG@THMAM-Co **6** (Figure 9), we evaluated the prepared material for its catalytic activity in water. We targeted Suzuki, Hiyama, and C–N cross-coupling reactions with the additional benefit of using a non-noble-metal catalyst instead of commonly applied palladium catalysts for such coupling reactions.^41−46^

### Suzuki and Hiyama Cross-Coupling Reactions

Catalyst **6** with a remarkable low loading of only 0.6 mol % showed high activity for Suzuki (Table 1) and Hiyama (Table 2) cross-coupling reactions of aryl iodides and bromides. Under optimized conditions (see the Supporting Information for screening details, Tables S1 and S2), electronically and sterically varied aryl iodides and aryl bromides were successfully coupled with a representative set of phenylboronic acids or triethoxyphenylsilane. While aryl iodides (70–95%) gave generally higher yields than aryl bromides (60–86%), aryl chlorides proved to be unreactive in the title transformations.

**Table 1:**
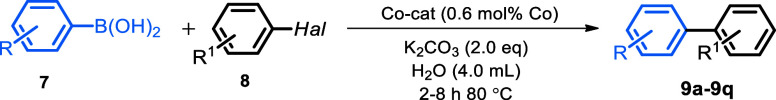
Suzuki–Miyaura Cross–Coupling Reactions of Different Aryl Halides with Phenylboronic Acid Derivatives Catalyzed by γ-Fe_2_O_3_@PEG@THMAM-Co **6**^a^

entry	**9**	halide	R	R^1^	yield [%]^b^
1	**9a**	I/Br	H	H	94/82
2	**9b**	I/Br	H	*p*-NH_2_	80/65
3	**9c**	I/Br	H	*o*-NH_2_	74/65
4	**9d**	I/Br	H	*m*-NH_2_	75/60
5	**9e**	I/Br	H	*p*-OMe	90/80
6	**9f**	I/Br	H	*p*-Me	92/78
7	**9g**	I/Br	H	*o*-Me	85/60
8	**9h**	I	H	*p*-Et	88
9	**9i**	I/Br	H	*p*-COH	92/86
10	**9j**	I/Br	H	COMe	90/78
11	**9k**	I/Br	*p*-CO_2_H	*p*-NO_2_	90/75
12	**9L**	I/Br	H	*p*-CN	91/80
13	**9m**	I	H	*o*-CN	90
14	**9n**	I/Br	H	*p*-NO_2_	94/85
15	**9o**	I/Br	H	*o*-NO_2_	85/70
16	**9p**	I/Br	H	*m*-NO_2_	80/70
17	**9q**	I/Br	*m*-NO_2_	*p*-NO_2_	85/70

a Phenylboronic acid (1.2 mmol), aryl halide (1.0 mmol).

b Isolated yield for coupling aryl iodides/aryl bromides.

**Table 2:**
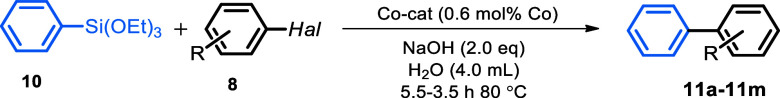
Hiyama Cross-Coupling Reactions of Different Aryl Halides with Triethoxyphenylsilane Catalyzed by γ-Fe_2_O_3_@PEG@THMAM-Co **6**^a^

entry	**11**	halide	R	yield [%]^b^
1	**11a**	I/Br	H	93/85
2	**11b**	I/Br	*p*-NH_2_	85/70
3	**11c**	I/Br	*o*-NH_2_	80/75
4	**11d**	I/Br	*m*-NH_2_	70/60
5	**11e**	I/Br	*p*-OMe	90/80
6	**11f**	I/Br	*p*-Me	88/70
7	**11g**	I/Br	*p*-COH	94/85
8	**11h**	I	COMe	92
9	**11i**	I/Br	*p*-CN	89/75
10	**11j**	I	*o*-CN	89
11	**11k**	I/Br	*p*-NO_2_	95/84
12	**11L**	I	*o*-NO_2_	85/75
13	**11m**	I/Br	*m*-NO_2_	85

a Triethoxyphenylsilane (1.5 mmol), aryl halide (1.0 mmol).

b Isolated yield for coupling aryl iodides/aryl bromides.

**Table 3:**
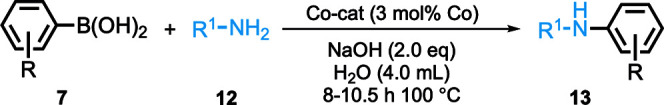
C–N Coupling Reactions of Different Amines with Phenylboronic Acid Derivatives Catalyzed by γ-Fe_2_O_3_@PEG@THMAM-Co **6**^a^

entry	R	amine	yield [%]^b^
1	H	1*H*-pyrrole	85
2	*p*-Cl	1*H*-pyrrole	90
3	H	morpholine	80
4	*p*-NH_2_	morpholine	75
5	*p*-NH_2_	piperidine	72
6	H	4-methoxyaniline	90
7	H	*p*-toluidine	84
8	H	aniline	90

a Phenylboronic acid (1.0 mmol), amine (1.5 mmol).

b Isolated yield.

### C–N Coupling Reactions

Likewise, the performance of **6** was evaluated for the coupling of phenylboronic acids and amines (see the Supporting Information for screening details, Table S3). An increased catalyst loading (3 mol %) was necessary compared to that in the previously discussed coupling reactions (vide supra) to achieve high yields (72–90%).

### Recyclability Study

Recyclability of **6** was evaluated for all three reaction types previously investigated, i.e., Suzuki, Hiyama, and C–N couplings, for 5 consecutive runs each (Table 4), giving consistent high yields with only a minimal decrease (≤5%) in activity. Capitalizing on the iron oxide core of **6**, recovery of the catalyst by application of an external magnet was facile and quantitative.

**Table 4:** Recyclability of **6** in Suzuki, Hiyama, and C–N Coupling Reactions

run^a^	Suzuki^b^	Hiyama^c^	C–N-coupling^d^
1	94	93	90
2	94	93	88
3	92	93	88
4	90	90	85
5	90	91	85

a Suzuki reaction cf Table 1, entry 1 (**9a**); Hiyama reactions cf Table 2, entry 1 (**11a**), C–N-coupling cf Table 3, entry 2 (**13b**).

b Yield of **9a**.

c Yield of **11a**.

d Yield of **13b**.

**Figure 10:**
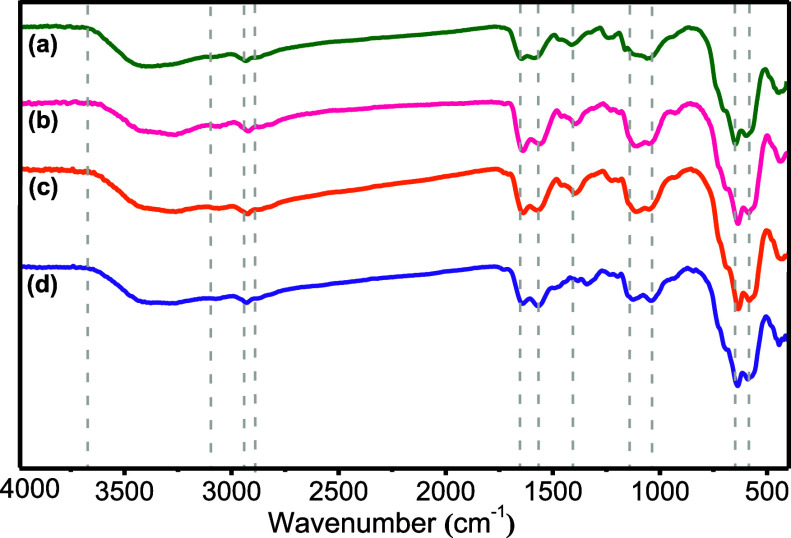
FT–IR spectra of the γ-Fe_2_O_3_@PEG@THMAM-Co catalyst **6** (a) before use and FT–IR spectra of the γ-Fe_2_O_3_@PEG@THMAM-Co catalyst **6** after 5 runs for (b) Suzuki, (c) Hiyama, and (d) C–N coupling.

FT–IR analysis was performed for reisolated **6** after five runs for all reaction types (Figure 10a–d), demonstrating the stability of the catalyst over time.

**Figure 11:**
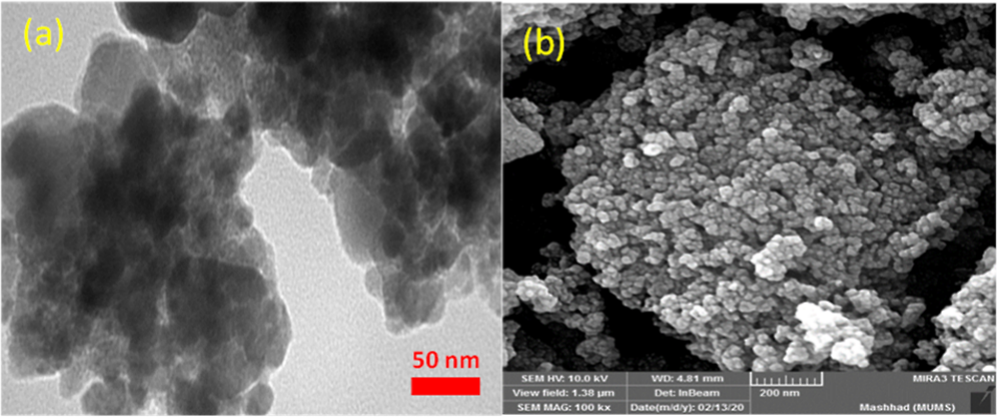
(a) TEM and (b) FE-SEM images recovered of γ-Fe_2_O_3_@PEG@THMAM-Co **6** after the 5th run for the model Suzuki reaction (cf Table 4) under optimized reaction conditions.

The comparison of TEM and SEM images of fresh and used catalysts after 5 runs in Suzuki coupling also showed no significant changes in particle size and morphology (Figure 11a,b). However, some agglomeration can be seen in the TEM image that is associated with dipole–dipole interactions of MNPs (Figures 11a,b, cf. Figures 3f and 4).

**Figure 12:**
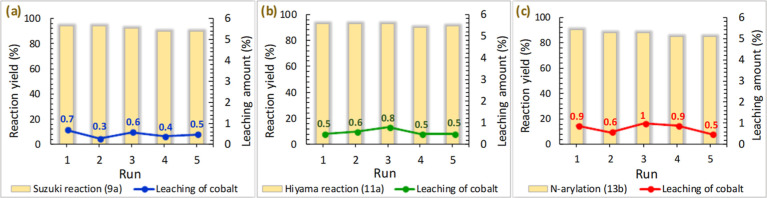
Recycling and determination of the amount of metal leaching of the catalyst during five runs under optimal reaction conditions (cf. Table 4): (a) Suzuki coupling for **9a**. (b) Hiyama reaction for **11a**. (c) C–N coupling for **13b**.

ICP analysis revealed low leaching of cobalt between ≤1 wt % for each run based on the initial cobalt content (0.6 mol % in Suzuki and Hiyama couplings and 3 mol % in C–N-coupling) used (Figure 12).

## Conclusions

In conclusion, we have developed an efficient protocol using water-dispersible, magnetically retrievable, and reusable cobalt catalyst γ-Fe_2_O_3_@PEG@THMAM-Co **6** in water for Suzuki, Hiyama, and C–N-couplings of aryl iodides and bromides, offering an alternative to the corresponding palladium-catalyzed transformations. While Suzuki couplings for aryl bromides and aryl iodides with “homeopathic amounts of palladium nanoparticles” (0.3–50 ppm) have been reported, it should be noted that taking the cost of palladium vs cobalt into account, the amount of cobalt used here would be equivalent to catalysis with 3 ppm palladium.

## Supporting Information Available

Research Details, experimental procedures, full characterization of the products, and NMR spectra (PDF)
