# Supplementary material for: Water-Dispersible, Magnetically Recyclable Heterogeneous Cobalt Catalyst for C–C and C–N Cross-Coupling Reactions in Aqueous Media
Source: ACS Omega. 2024 Jul 9;9(29):31393–400. doi: 10.1021/acsomega.3c10462 (PMC11270699; doi:10.1021/acsomega.3c10462)
Supplement: Supplementary file 1 — ao3c10462_si_001.pdf [file ao3c10462_si_001.pdf]

A Water-Dispersible, Magnetically Recyclable Heterogeneous Cobalt Catalyst for C–C and C-N Cross-Coupling Reactions in Aqueous Media

Safoora Sheikh,[†,‡] Aditya Bhattacharyya,[‡] Marco A. Henriquez,[‡] Mohammad Ali Nasseri,[*,†] Mohammad Chahkandi,[§] Ali Allahresani,[†] Oliver Reiser,[*,‡]

[†]Department of Chemistry, University of Birjand, P. O. Box 97175-615, Birjand, Iran

[‡]Institut für Organische Chemie, Universität Regensburg, Universitätsstraße 31, Regensburg, German

[§]Department of Chemistry, Hakim Sabzevari University, P. O. Box 96179-76487, Sabzevar, Iran

Table of Contents

1- Experimental Procedures 2-8

2- Characterization of products 9-27

3- NMR Spectra 28-44

General

All starting materials were commercially available and used as received. Thin layer chromatography (TLC) was performed on silica-gel 60 F_{254} plates and UV light was used for visualization and also by GC-FID on a Shimadzu GC-16A instrument using a 25 m CBP1-S25 (0.32 mm ID, 0.5 μm coating) capillary column. Melting points were determined on a Tropical Labequip apparatus. The FT-IR spectra (JASCO–FT-IR 4600) were recorded using KBr pellet. The ^{1}H-NMR and ^{13}C-NMR spectra were recorded on a Bruker Avance DPX–300 spectrometer in the deuterated solvents ($CDCl_3$), using tetramethylsilane (TMS) as an internal standard. The Field emission scanning electron microscopy (FE-SEM) images of the prepared catalysts were taken on a Tescan MIRA3. EDX analysis was performed using a FESEM (JEOL–7600F–Oxford) equipped with a spectrometer of energy dispersion of X-ray. The presence of those elements was confirmed using the point elemental mapping (Tescan–Mira 3–SAMX). The microscopic images and size distribution of the catalyst NPs were performed using TEM (Philips–EM208) operating at 100 kV voltage. The X-ray diffraction (XRD) pattern was recorded by an X'pertpro (Philips) instrument employing Cu Kα radiation (λ = 1.5418 Å), at a scanning speed of 2 °C/min from 10 to 80 °C (2θ). TGA analysis of the samples were performed using a Q600 model from TA company made in U.S.A under nitrogen atmosphere with a heating rate of 15 °C/min in the temperature range of 25–800 °C. The content of Co in the catalyst was determined by OPTIMA 7300DV ICP analyzer. All the measured yields refer to the isolated products after purification by column chromatography. All the products are characterized by NMR spectroscopy and comüared to literarture data. γ–Fe_2O_3 MNPs **1** were synthesized by co-precipitation[1] and further converted to γ-Fe_2O_3@PEG-400 MNPs **2** following protocols described in the literature.[2]

Preparation of γ-Fe_2O_3@PEG@APTES 3. γ-Fe_2O_3@PEG MNPs **2** (1.0 g) were dispersed by sonication in anhydrous toluene (30 mL) for 30 min. Then, (3-aminopropyl)triethoxysilane (APTES, 0.885 g, 0.83 mL, 4.0 mmol) was added dropwise while stirring under N_2 at 25 °C. The reaction mixture was subsequently heated under reflux for 24 h. After completion, the solid product was separated from the solvent by an

external magnet and washed twice with anhydrous toluene and diethyl ether followed by drying under vacuum at 50 °C overnight to obtain **3** (1.1 g).

Preparation of γ-Fe_2O_3@PEG@APTES@Mac 4. Methyl acrylate (MAc, 7 mL, 6.6 g, 0.077 mol) was added dropwise to the stirred mixture of **3** (1.0 g) dispersed in ethanol (30 mL) under N_2 atmosphere at 25 °C, followed by heating at 40 °C for 24 h. After completion, the solid product was separated by an external magnet and washed with ethanol three times followed by vacuum drying at 50 °C overnight to obtain **4** (1.2 g).

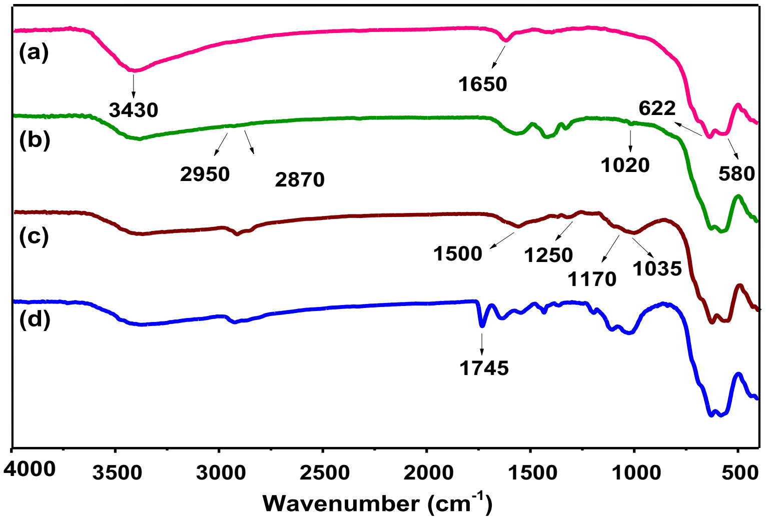

Figure S1. FT–IR spectra of the (a) γ–Fe_2O_3 **1**. (b) γ-Fe_2O_3@PEG **2**. (c) γ-Fe_2O_3@PEG@APTES **3**. (d) γ–Fe_2O_3@PEG@APTES@MAc **4**.

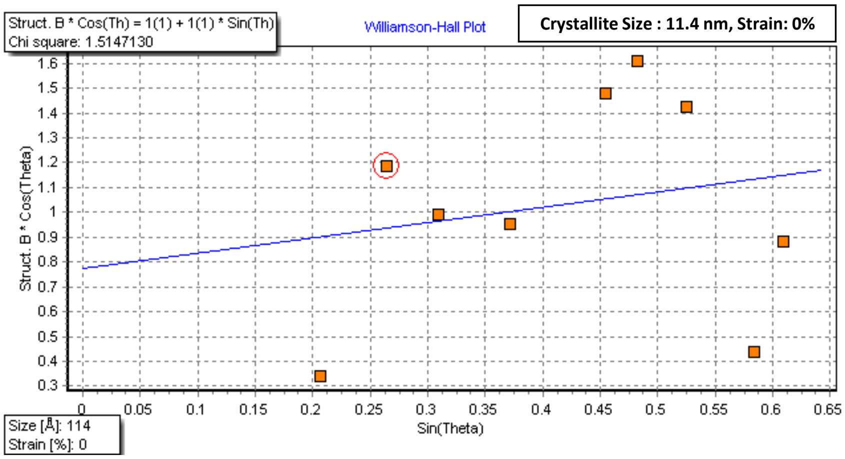

Figure S2. Williamson Hall plot, determination size and strain of γ-Fe_2O_3@PEG@THMAM-Co **6**.

Optimization study for the Suzuki cross coupling reaction

As the starting point, our investigations began with the study to identification of the optimal reaction conditions on the Suzuki cross coupling using γ-Fe_2O_3@PEG@THMAM-Co **6** as a hydrophilic catalyst (Table S1, entry 1–12). To this end, phenylboronic acid and aryl iodide were selected as test substrates to identify appropriate parameters on the progress of the target reaction. Thus, we checked the effect of temperature changes, amount of catalyst loading, type and amount of base used on the model reaction. Initially, 0.6 mol% of Co complex was exposed to the model substrates and 2 mmol of various base (Et_3N, KOH, NaOH, Cs_2CO_3, K_2CO_3) in water as the green solvent (4.0 mL) at 80 °C (Table S1, entry 1–5). When the model reaction was performed using Et_3N and Cs_2CO_3 bases (Table S1, entries 1 and 2), the formation of the biphenyl product **9a** reached to 75 and 85% yields, respectively. Also, changing type of base to KOH resulted in an improved yield of **9a** (90%, Table S1, entry 3). Even better results were obtained to the formation of **9a** when NaOH (95%, entry 4) and K_2CO_3 (95%, Table S1, entry 5) were used as bases. Subsequently, we investigated the effect of changes in the temperature on advancing the model reaction (Table S1, entries 5–8). In this series of experiments, we found that when the model reaction was performed at the room temperature, the efficiency markedly dropped and even after 12 hours of continuous reaction, the efficiency didn't increase (30%, Table S1, entry 6). Also, the decrease of the temperature to 70 °C led to incomplete production (80%, Table S1, entry 7). Additionally, increasing the temperature to 10 °C had no effect either on the formation of **9a** (94%, Table S1, entry 8). In the next phase of our research, the amount loading of Co catalyst was investigated. The best result was obtained with 0.6 mol% loading of the catalyst (0.6 mol%

of Co, 0.007 g of catalyst) that led to the formation of the biphenyl product **9a** in 94% yield (see Table S1 for more details). Furthermore, in the absence of K_2CO_3 as a mild base, the formation of the product **9a** was just 10% (Table S1, entry 11). Decreasing the amount of base used (K_2CO_3, 1 mol) did not have a favorable effect leading to moderate conversion to **9a** (73%, Table S1, entry 12). In general, the best results were found using 0.6 mol% loading of the γ-Fe_2O_3@PEG@THMAM-Co catalyst **6** (0.007 g), K_2CO_3 as a base (2 mmol) in H_2O (4 mL) at 80 °C for 180 min furnishing the compound **9a** in 94% isolated yield (see Table S1 for more details).

Table S1. Suzuki reaction of iodobenzene and phenylboronic acid catalyzed by γ-Fe_2O_3@PEG@THMAM-Co **6** in the water as a solvent under different conditions.[a]

Entry	Catalyst (mol %)	Base (mmol)	T (° C)	Time (h)	Yield (%)
1	0.6	Et_3N (2)	80	3	75
2	0.6	Cs_2CO_3 (2)	80	3	85
3	0.6	KOH (2)	80	3	90
4	0.6	NaOH (2)	80	3	94
**5**	**0.6**	**K_2CO_3 (2)**	**80**	**3**	**94**
6	0.6	K_2CO_3 (2)	r.t	12	30
7	0.6	K_2CO_3 (2)	70	3	80
8	0.6	K_2CO_3 (2)	90	3	94
9	0.7	K_2CO_3 (2)	80	3	94
10	0.5	K_2CO_3 (2)	80	3	85
11	0.6	-	80	12	10
12	0.6	K_2CO_3 (1)	80	3	73

[a]Reaction conditions: Iodobenzene (1.0 mmol), phenylboronic acid (1.2 mmol), H_2O (4.0 mL); All yields are isolated.

Optimization study for the Hiyama cross coupling reaction

In the next set of experiments, we extended our research by examining the performance of the γ-Fe_2O_3@PEG@THMAM-Co catalyst **6** on the Hiyama cross coupling reaction. For investigation of the optimal reaction conditions, triethoxyphenylsilane and aryl iodide were selected as test substrates to identify appropriate parameters on the progress of the Hiyama coupling as the target reaction. Hence, the effect of diverse parameters, including temperature, amount of catalyst loading, type and amount of base, were appraised on the progression of the model reaction using γ-Fe_2O_3@PEG@THMAM-Co catalyst **6** in water as the solvent (Table S2,

entries 1–10). Based on the results, the best yield was found by using 0.6 mol% loading of the γ-Fe_2O_3@PEG@THMAM-Co catalyst **6** (0.007 g), NaOH as a base (2 mmol) in water (4 mL) at 80 °C for 270 min producing compound **11a** in 93% isolated yield (see Table S2 to find more details, entries 1–10).

Table S2. Hiyama reaction of iodobenzene and triethoxyphenylsilane catalyzed by γ-Fe_2O_3@PEG@THMAM-Co **6** in the water as a solvent under different conditions.[a]

Entry	Catalyst (mol%)	Base (mmol)	T (° C)	Time (h)	Yield (%)
1	0.6	K_2CO_3 (2)	80	4.5	70
2	0.6	Et_3N (2)	80	4.5	80
3	0.6	KOH (2)	80	4.5	90
**4**	**0.6**	**NaOH (2)**	**80**	**4.5**	**93**
5	0.6	Cs_2CO_3 (2)	80	4.5	93
6	0.6	NaOH (2)	r.t	12	34
7	0.6	NaOH (2)	60	4.5	70
8	0.6	NaOH (2)	90	4.5	93
9	0.5	NaOH (2)	80	4.5	75
10	0.6	NaOH (1)	80	4.5	65

[a]Reaction conditions: Iodobenzene (1.0 mmol), triethoxyphenylsilane (1.5 mmol), H_2O (4.0 mL); All yields are isolated.

Optimization study for the C–N coupling reaction

Our investigations continued with the C–N coupling reaction, using γ-Fe_2O_3@PEG@THMAM-Co catalyst **6** in water as the solvent. In order to investigate the targeted process, 4-Cl-phenylboronic acid and 1*H*-pyrrole were selected as the test substrates to identify appropriate parameters on the progress of the C–N coupling reaction (Table S3, entries 1–8). Initially, 3 mol % of Co complex was exposed to the model substrates and 2 mmol of various base (Et_3N, NaOH, KOH) in water as a green solvent (4.0 mL), at 100 °C (Table S3, entries 1–3). In this part, we noticed that NaOH led to better yields of **13b** production and we decided to use NaOH as a base (90%, Table S3, entry 3). In subsequent experiment, we investigated the effect of temperature changes, on advancing the model reaction (Table S3, entries 3–5). On the basis of the results, the best yield reached at 100 °C that led to excellent production of 1**3b** (90%, Table

S3, entry 3). We found when was performed the model reaction at the room temperature, **13b** was produced in insignificant amount (Table S3, entry 5). Additionally, in the absence of NaOH as a base, formation of the product **13b** was insignificant (Table S3, entry 7). In the next step, the amount loading of Co catalyst was obtained 3 mol% (3 mol% of Co, 0.035 g of catalyst) that led to formation of the corresponding biphenyl product **13b** in 90% yield (see Table S3 for more details).

Table S3. C–N coupling reaction of 1*H*-pyrrole and 4-Cl-phenylboronic acid catalyzed by γ-Fe_2O_3@PEG@THMAM-Co **6** in the water as a solvent under different conditions.[a]

Entry	Catalyst (mol%)	Base (mmol)	T (° C)	Time (h)	Yield (%)[b]
1	3	Et_3N (2)	100	10	80
2	3	KOH (2)	100	10	87
**3**	**3**	**NaOH (2)**	**100**	**10**	**90**
4	3	NaOH (2)	90	10	80
5	3	NaOH (2)	r.t	10	trace
6	3	NaOH (1)	100	10	55
7	3	-	100	10	trace
8	2	NaOH (2)	100	10	70

[a]reaction conditions: 4-Cl-phenylboronic acid (1.0 mmol), 1*H*-pyrrole (1.5 mmol), H_2O (4.0 mL); All yields are isolated.

Catalytic performance

Catalytic efficiency of the γ-Fe_2O_3@PEG@THMAM-Co **6** complex in terms of the reaction time and yield was compared with the other reported catalysts within the Suzuki, Hiyama and C–N coupling reactions (see Table S4). The high activity of the catalyst in water medium as an abundant, and available solvent is notable. Other benefits of this catalytic methodology in comparison with the others catalytic methodologies listed in the Table S4 can be mentioned to simplicity of catalyst separation, higher yield of the corresponding products during the shorter reaction times, eco–friendly and economic aspects, as well as the use of available and inexpensive cobalt metal complex, especially in the C–N coupling reaction (see Table S4 for more details).

Table S4. Catalytic activities of the γ-Fe_2O_3@PEG@THMAM-Co **6** was compared with some catalysts reported for the Suzuki, Hiyama and C-N coupling reactions.

Prod.	Entry	Catalyst (mol%)	Reaction conditions	Tim (h)	Yield (%)	Ref.
**9a**[a]	1	γ-Fe_2O_3@PEG@THMAM-Co	K_2CO_3/H_2O/80 °C	3.5	94	This work
	2	TiO_2-Pd/Au-Al_2O_3	NaOH/CH_3CN/r.t	1	71	3
	3	GO/Au-Pd	K_2CO_3/EtOH:H_2O/r.t	2	78	4
	4	NHC-Pd(II) complex	Cs_2CO_3/THF /80 °C	12	88	5
	5	Bis (oxamato) palladate (II)	Et_3N/Bu_4NBr/120 °C	2	78	6
	6	Pd-Fe_3O_4	Na_2CO_3/DME:H_2O/Reflux	24	70	7
	7	Pd@Mag-MSN	K_2CO_3/Dioxane/80 °C	6	77	8
	8	Xerogel g_1-MNPs	K_2CO_3 /MeOH/60 °C	5	89	9
**11a**[b]	9	γ-Fe_2O_3@PEG@THMAM-Co	NaOH/H_2O/80 °C	4.5	93	This work
	10	Fe_3O_4@CS-Co	Et_3N/DMF/100 °C	3	91	10
	11	PS-tsu-Pd(II)	LiOH/Ethyleneglycol:H_2O/100 °C	10	80	11
	12	NHC-Pd(II)	CsF/DMF/120 ºC	90	99	12
	13	Palladium chloride	TBAF/Toluene/100 °C	10	90	13
**13h**[c]	14	γ-Fe_2O_3@PEG@THMAM-Co	NaOH/H_2O/90 °C	10.5	90	This work
	15	Ni cat	K_3PO_4/tuloene:DMF/70 °C	8	75	14
	16	Fe_3O_4@PEG/Cu-Co	H_2O/ Reflux	7	65	15

[a]Suzuki: reaction of iodobenzene and phenylboronic acid. [b]Hiyama: reaction of iodobenzene and triethoxyphenylsilane. [c]Reaction of phenylboronic acid and aniline.

Data of Suzuki Products

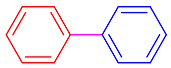

biphenyl (9a)
Chemical Formula: $C_{12}H_{10}$
Molecular Weight: 154.21 g/mol

Biphenyl (**9a**). This compound is known in the literature.[15]

Phenylboronic acid (146.4 mg, 1.2 mmol) was reacted with iodobenzene (204.0 mg, 1.00 mmol), K_2CO_3 (276.41 mg, 2.00 mmol) and γ-Fe_2O_3@ γ-Fe_2O_3@PEG@THMAM-Co **6** (0.6 mol%, 0.007 g) in water (4 mL) at 80 °C to yield 94% (144.9 mg) biphenyl (**9a**). Chromatography: *n*-hexane/EtOAc, 8:2. Chromatography: n-hexane/EtOAc, 8:2. **^{1}H-NMR** ($CDCl_3$, 300 MHz) δ 7.37–7.66 (m, 9H). **^{13}C NMR** (75 MHz, $CDCl_3$): δ 127.2, 128.6, 129.2, 141.3.

Phenylboronic acid (146.4 mg, 1.2 mmol) was reacted with bromobenzene (157.0 mg, 1.00 mmol), K_2CO_3 (276.41 mg, 2.00 mmol) and γ-Fe_2O_3@PEG@THMAM-Co **6** (0.6 mol%, 0.007 g) in water (4 mL) at 80 °C to yield 82% (126.4 mg) biphenyl (**9a**). Chromatography: *n*-hexane/EtOAc, 8:2. **^{1}H-NMR** ($CDCl_3$, 300 MHz) δ 7.37–7.64 (m, 9H). **^{13}C NMR** (75 MHz, $CDCl_3$): δ 127.2, 128.6, 129.2, 141.2.

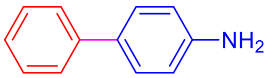

4-amine-1,1'-biphenyl (9b)
Chemical Formula: $C_{12}H_{11}N$
Molecular Weight: 169 g/mol

4-Amine-1,1'-biphenyl (**9b**). This compound is known in the literature.[15]

Phenylboronic acid (146.4 mg, 1.2 mmol) was reacted with 4-iodoaniline (219.0 mg, 1.00 mmol), K_2CO_3 (276.4 mg, 2.00 mmol) and γ-Fe_2O_3@PEG@THMAM-Co **6** (0.6 mol%, 0.007 g) in water (4 mL) at 80 °C to yield 80% (135.2 mg) 4-amine-1,1'-biphenyl (**9b**). Chromatography: *n*-hexane/EtOAc, 8:2. **^{1}H-NMR** (DMSO, 250 MHz) δ 5.25 (s, 2H, NH_2), 6.66–7.56 (m, 9H, Ar*H*). **^{13}C NMR** (75 MHz, $CDCl_3$) δ 114.7, 125.8, 126.1, 127.6, 127.8, 129.1, 141.1, 148.8.

Phenylboronic acid (146.4 mg, 1.2 mmol) was reacted with 4-bromoaniline (172.0 mg, 1.00 mmol), K_2CO_3 (276.4 mg, 2.00 mmol) and γ-Fe_2O_3@PEG@THMAM-Co **6** (0.6 mol%, 0.007 g) in water (4 mL) at 80 °C to yield 65% (109.8 mg) 4-amine-1,1'-biphenyl (**9b**). Chromatography: *n*-hexane/EtOAc, 8:2. **^{1}H-NMR** (DMSO,

250 MHz) δ 5.27 (s, 2H, NH_2), 6.65–7.55 (m, 9H, Ar*H*). ^{13}**C NMR** (75 MHz, $CDCl_3$) δ 114.7, 125.7, 126.2, 127.6, 127.8, 129.2, 141.3, 148.9.

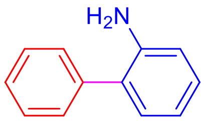

[1,1'-biphenyl]-2-amine (9c)
Chemical Formula: $C_{12}H_{11}N$
Molecular Weight: 169.21 g/mol

[1,1'-Biphenyl]-2-amine (**9c**). This compound is known in the literature.[15]

Phenylboronic acid (146.4 mg, 1.2 mmol) was reacted with 2-iodoaniline (219.0 mg, 1.00 mmol), K_2CO_3 (276.41 mg, 2.00 mmol) and γ-Fe_2O_3@PEG@THMAM-Co **6** (0.6 mol%, 0.007 g) in water (4 mL) at 80 °C to yield 74% (125.2 mg) [1,1'-biphenyl]-2-amine (**9c**). Chromatography: *n*-hexane/EtOAc, 8:2. ^{1}**H-NMR** (DMSO, 250 MHz) δ 4.76 (s, 2H), 6.65–7.50 (m, 9H). ^{13}**C NMR** (75 MHz, $CDCl_3$) δ 115.7, 117.2, 126.2, 127.2, 128.6, 129.1, 129.2, 130.5, 140.1, 145.4.

Phenylboronic acid (146.4 mg, 1.2 mmol) was reacted with 2-bromoaniline (172.0 mg, 1.00 mmol), K_2CO_3 (276.41 mg, 2.00 mmol) and γ-Fe_2O_3@PEG@THMAM-Co **6** (0.6 mol%, 0.007 g) in water (4 mL) at 80 °C to yield 65% (109.9 mg) [1,1'-biphenyl]-2-amine (**9c**). Chromatography: *n*-hexane/EtOAc, 8:2. ^{1}**H-NMR** (DMSO, 250 MHz) δ 4.76 (s, 2H), 6.66–7.51 (m, 9H). ^{13}**C NMR** (75 MHz, $CDCl_3$) δ 115.9, 117.2, 126.2, 127.7, 128.8, 129.2, 129.5, 130.5, 140.3, 145.1.

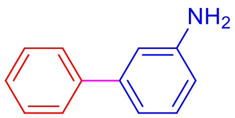

3-amine-1,1'-biphenyl (9d)
Chemical Formula: $C_{12}H_{11}N$
Molecular Weight: 169 g/mol

3-Amine-1,1'-biphenyl (**9d**). This compound is known in the literature.[15]

Phenylboronic acid (146.4 mg, 1.2 mmol) was reacted with 3-iodoaniline (219.0 mg, 1.00 mmol), K_2CO_3 (276.41 mg, 2.00 mmol) and γ-Fe_2O_3@PEG@THMAM-Co **6** (0.6 mol%, 0.007 g) in water (4 mL) at 80 °C to yield 75% (126.7 mg) 3-amine-1,1'-biphenyl (**9d**). Chromatography: *n*-hexane/EtOAc, 8:2. ^{1}**H-NMR** (DMSO,

250 MHz) δ 4.78 (s, 2H), 6.58–7.52 (m, 9H). **^{13}C NMR** (75 MHz, $CDCl_3$) δ 114.3, 118.3, 118.8, 127.2, 127.9, 129.2, 130.8, 140.2, 140.5, 149.1.

Phenylboronic acid (146.4 mg, 1.2 mmol) was reacted with 3-bromoaniline (172.0 mg, 1.00 mmol), K_2CO_3 (276.41 mg, 2.00 mmol) and γ-Fe_2O_3@PEG@THMAM-Co **6** (0.6 mol%, 0.007 g) in water (4 mL) at 80 °C to yield 60% (101.4 mg) 3-amine-1,1'-biphenyl (**9d**). Chromatography: *n*-hexane/EtOAc, 8:2. **^{1}H-NMR** (DMSO, 250 MHz) δ 4.78 (s, 2H), 6.58–7.54 (m, 9H). **^{13}C NMR** (75 MHz, $CDCl_3$) δ 114.5, 118.6, 118.9, 127.2, 127.9, 129.2, 130.6, 140.1, 140.6, 149.1.

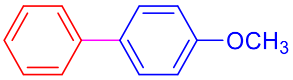

4-methoxy-1,1'-biphenyl (9e)
Chemical Formula: $C_{13}H_{12}O$
Molecular Weight: 184 g/mol

4-Methoxy-1,1'-biphenyl (**9e**). This compound is known in the literature.[16]

Phenylboronic acid (146.4 mg, 1.2 mmol) was reacted with 1-iodo-4-methoxybenzene (234.0 mg, 1.00 mmol), K_2CO_3 (276.41 mg, 2.00 mmol) and γ-Fe_2O_3@PEG@THMAM-Co **6** (0.6 mol%, 0.007 g) in water (4 mL) at 80 °C to yield 90% (165.6 mg) 4-methoxy-1,1'-biphenyl (**9e**). Chromatography: *n*-hexane/EtOAc, 8:2. **^{1}H-NMR** ($CDCl_3$, 300 MHz) δ 2.92 (s, 3H), 7.00–7.61 (m, 9H, Ar*H*). **^{13}C NMR** (75 MHz, $CDCl_3$) δ 55.8, 114.6, 127.1, 127.2, 128.6, 129.2, 134.2, 141.3, 159.6.

Phenylboronic acid (146.4 mg, 1.2 mmol) was reacted with 1-bromo-4-methoxybenzene (187.0 mg, 1.00 mmol), K_2CO_3 (276.41 mg, 2.00 mmol) and γ-Fe_2O_3@PEG@THMAM-Co **6** (0.6 mol%, 0.007 g) in water (4 mL) at 80 °C to yield 80% (147.2 mg) 4-methoxy-1,1'-biphenyl (**9e**). Chromatography: *n*-hexane/EtOAc, 8:2. **^{1}H-NMR** ($CDCl_3$, 300 MHz) δ 2.92 (s, 3H), 7.01–7.63 (m, 9H, Ar*H*). **^{13}C NMR** (75 MHz, $CDCl_3$) δ 55.7, 114.6, 127.2, 127.3, 128.8, 129.2, 134.4, 141.1, 159.9.

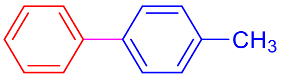

4-methyl-1,1'-biphenyl (9f)
Chemical Formula: $C_{13}H_{12}$
Molecular Weight: 168 g/mol

4-Methyl-1,1'-biphenyl (**9f**). This compound is known in the literature.[15]

Phenylboronic acid (146.4 mg, 1.2 mmol) was reacted with 1-iodo-4-methylbenzene (218.0 mg, 1.00 mmol), K_2CO_3 (276.41 mg, 2.00 mmol) and γ-Fe_2O_3@PEG@THMAM-Co **6** (0.6 mol%, 0.007 g) in water (4 mL) at 80 °C to yield 92% (154.5 mg) 4-methyl-1,1'-biphenyl (**9f**). Chromatography: *n*-hexane/EtOAc, 8:2. **^{1}H-NMR** ($CDCl_3$, 300 MHz) δ 2.47 (s, 3H), 7.26–7.68 (m, 9H). **^{13}C NMR** (75 MHz, $CDCl_3$) δ 21.6, 127.5, 127.7, 127.9, 129.2, 130.0, 137.5, 138.8, 141.6.

Phenylboronic acid (146.4 mg, 1.2 mmol) was reacted with 1-bromo-4-methylbenzene (171.0 mg, 1.00 mmol), K_2CO_3 (276.41 mg, 2.00 mmol) and γ-Fe_2O_3@PEG@THMAM-Co **6** (0.6 mol%, 0.007 g) in water (4 mL) at 80 °C to yield 78% (131.0 mg) 4-methyl-1,1'-biphenyl (**9f**). Chromatography: *n*-hexane/EtOAc, 8:2. **^{1}H-NMR** ($CDCl_3$, 300 MHz) δ 2.47 (s, 3H), 7.26–7.67 (m, 9H). **^{13}C NMR** (75 MHz, $CDCl_3$) δ 21.7, 127.5, 127.5, 127.9, 129.2, 130.0, 137.4, 138.5, 141.6.

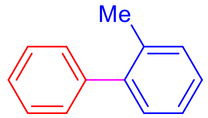

2-methyl-1,1'-biphenyl (9g)
Chemical Formula: $C_{13}H_{12}$
Molecular Weight: 168 g/mol

2-Methyl-1,1'-biphenyl (**9g**). This compound is known in the literature.[15]

Phenylboronic acid (146.4 mg, 1.2 mmol) was reacted with 1-iodo-2-methylbenzene (218.0 mg, 1.00 mmol), K_2CO_3 (276.41 mg, 2.00 mmol) and γ-Fe_2O_3@PEG@THMAM-Co **6** (0.6 mol%, 0.007 g) in water (4 mL) at 80 °C to yield 85% (142.8 mg) 2-methyl-1,1'-biphenyl (**9g**). Chromatography: *n*-hexane/EtOAc, 8:2. . **^{1}H NMR** ($CDCl_3$, 250 MHz) δ 2.33 (s, 3H), 7.26–9.13 (m, 9H). **^{13}C NMR** (75 MHz, $CDCl_3$) δ 21.0, 126.2, 127.2, 127.7, 128.5, 129.7, 130.3, 130.5, 130.8, 135.8, 142.4.

Phenylboronic acid (146.4 mg, 1.2 mmol) was reacted with 1-bromo-2-methylbenzene (171.0 mg, 1.00 mmol), K_2CO_3 (276.41 mg, 2.00 mmol) and γ-Fe_2O_3@PEG@THMAM-Co **6** (0.6 mol%, 0.007 g) in water (4 mL) at 80 °C to yield 60% (100.8 mg) 2-methyl-1,1'-biphenyl (**9g**). Chromatography: *n*-hexane/EtOAc, 8:2. . **^{1}H NMR** ($CDCl_3$, 250 MHz) δ 2.35 (s, 3H), 7.28–9.15 (m, 9H). **^{13}C NMR** (75 MHz, $CDCl_3$) δ 21.1, 126.3, 127.2, 127.8, 128.5, 129.7, 130.4, 130.7, 130.9, 135.8, 142.5.

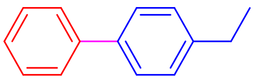

4-ethyl-1,1'-biphenyl (**9h**)
Chemical Formula: $C_{14}H_{14}$
Molecular Weight: 182.26 g/mol

4-Ethyl-1,1'-biphenyl (**9h**). This compound is known in the literature.[17]

Phenylboronic acid (146.4 mg, 1.2 mmol) was reacted with 1-iodo-4-ethylbenzene (232.0 mg, 1.00 mmol), K_2CO_3 (276.41 mg, 2.00 mmol) and γ-Fe_2O_3@PEG@THMAM-Co **6** (0.6 mol%, 0.007 g) in water (4 mL) at 80 °C to yield 88% (160.3 mg) 4-ethyl-1,1'-biphenyl (**9h**). Chromatography: *n*-hexane/EtOAc, 8:2. **^{1}H-NMR** ($CDCl_3$, 300 MHz) *δ* 1.55 (t, 3H), 2.95 (q, 2H), 7.51–7.88 (m, 9H). **^{13}C-NMR** (75 MHz, $CDCl_3$) δ 15.9, 28.8, 127.2, 127.3, 127.6, 128.5, 128.9, 138.8, 141.4, 143.5.[15]

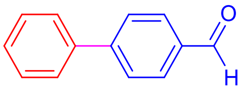

[1,1'-biphenyl]-4-carbaldehyde (9i)
Chemical Formula: $C_{13}H_{10}O$
Molecular Weight: 182 g/mol

[1,1'-biphenyl]-4-carbaldehyde (**9i**). This compound is known in the literature.[15]

Phenylboronic acid (146.4 mg, 1.2 mmol) was reacted with 4-iodobenzaldehyde (232.0 mg, 1.00 mmol), K_2CO_3 (276.41 mg, 2.00 mmol) and γ-Fe_2O_3@PEG@THMAM-Co **6** (0.6 mol%, 0.007 g) in water (4 mL) at 80 °C to yield 92% (167.4 mg) [1,1'-biphenyl]-4-carbaldehyde (**9i**). Chromatography: *n*-hexane/EtOAc, 8:2. **^{1}H-NMR** ($CDCl_3$, 300 MHz) *δ* 7.44–8.21 (m, 9H), 10.10 (s, 1H). **^{13}C NMR** (75 MHz, $CDCl_3$) *δ* 127.8, 128.9, 129.4, 129.9, 130.7, 135.6, 140.2, 147.7, 192.4.

Phenylboronic acid (146.4 mg, 1.2 mmol) was reacted with 4-bromobenzaldehyde (185.0 mg, 1.00 mmol), K_2CO_3 (276.41 mg, 2.00 mmol) and γ-Fe_2O_3@PEG@THMAM-Co **6** (0.6 mol%, 0.007 g) in water (4 mL) at 80 °C to yield 86% (156.5 mg) [1,1'-biphenyl]-4-carbaldehyde (**9i**). Chromatography: *n*-hexane/EtOAc, 8:2. **^{1}H-NMR** ($CDCl_3$, 300 MHz) *δ* 7.45–8.24 (m, 9H), 10.10 (s, 1H). **^{13}C NMR** (75 MHz, $CDCl_3$) *δ* 127.9, 128.9, 129.4, 129.9, 130.5, 135.6, 140.1, 147.7, 192.8.

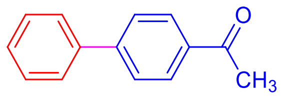

4-Acetylbiphenyl (9j)
Chemical Formula: $C_{14}H_{12}O$
Molecular Weight: 196 g/mol

4-Acetylbiphenyl (**9j**). This compound is known in the literature.[18]

Phenylboronic acid (146.4 mg, 1.2 mmol) was reacted with 1-(4-iodophenyl)ethan-1-one (246.0 mg, 1.00 mmol), K_2CO_3 (276.41 mg, 2.00 mmol) and γ-Fe_2O_3@PEG@THMAM-Co **6** (0.6 mol%, 0.007 g) in water (4 mL) at 80 °C to yield 90% (176.4 mg) [1,1'-biphenyl]-4-carbaldehyde (**9j**). Chromatography: *n*-hexane/EtOAc, 8:2. **^{1}H-NMR** ($CDCl_3$, 300 MHz) δ 2.69 (s, 3H), 7.29–8.09 (m, 9H). **^{13}C NMR** (75 MHz, $CDCl_3$) δ 26.7, 127.25, 127.29, 128.2, 128.9, 130.0, 135.8, 139.8, 145.8, 197.8.

Phenylboronic acid (146.4 mg, 1.2 mmol) was reacted with 1-(4-bromophenyl)ethanone (199.0 mg, 1.00 mmol), K_2CO_3 (276.41 mg, 2.00 mmol) and γ-Fe_2O_3@PEG@THMAM-Co **6** (0.6 mol%, 0.007 g) in water (4 mL) at 80 °C to yield 78% (152.8 mg) [1,1'-biphenyl]-4-carbaldehyde (**9j**). Chromatography: *n*-hexane/EtOAc, 8:2. **^{1}H-NMR** ($CDCl_3$, 300 MHz) δ 2.68 (s, 3H), 7.29–8.08 (m, 9H). **^{13}C NMR** (75 MHz, $CDCl_3$) δ 26.6, 127.25, 127.29, 128.5, 128.8, 130.0, 135.8, 139.8, 145.5, 197.8.

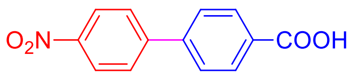

4'-nitro-[1,1'-biphenyl]-4-carboxylic acid **(9k)**
Chemical Formula: $C_{13}H_9NO_4$
Molecular Weight: 243.21 g/mol

4'-Nitro-[1,1'-biphenyl]-4-carboxylic acid (**9k**).

4-Nitrophenylboronic acid (200.28 mg, 1.2 mmol) was reacted with 4-iodobenzoic acid (248.0 mg, 1.00 mmol), K_2CO_3 (276.41 mg, 2.00 mmol) and γ-Fe_2O_3@PEG@THMAM-Co **6** (0.6 mol%, 0.007 g) in water (4 mL) at 80 °C to yield 90% (218.8 mg) 4'-Nitro-[1,1'-biphenyl]-4-carboxylic acid (**9k**). Chromatography: *n*-hexane/EtOAc, 8:2. **^{1}H-NMR** (DMSO, 300 MHz) δ 7.89–8.35 (m, 8H), 13.17 (s, 1H). **^{13}C NMR** (75 MHz, $CDCl_3$) δ 124.6, 127.9, 128.7, 130.5, 131.4, 143.2, 145.8, 147.5, 167.3.

4-Nitrophenylboronic acid (200.28 mg, 1.2 mmol) was reacted with 4-bromobenzoic acid (201.0 mg, 1.00 mmol), K_2CO_3 (276.41 mg, 2.00 mmol) and γ-Fe_2O_3@PEG@THMAM-Co **6** (0.6 mol%, 0.007 g) in water (4 mL) at 80 °C to yield 75% (182.4 mg) 4'-Nitro-[1,1'-biphenyl]-4-carboxylic acid (**9k**). Chromatography: *n*-

hexane/EtOAc, 8:2. **^{1}H-NMR** (DMSO, 300 MHz) δ 7.90–8.36 (m, 8H), 13.15 (s, 1H). **^{13}C NMR** (75 MHz, $CDCl_3$) δ 124.6, 127.7, 128.7, 130.8, 131.4, 143.3, 145.4, 147.5, 167.4.

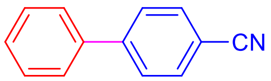

4-cyano-1,1'-biphenyl (9l)
Chemical Formula: $C_{13}H_9N$
Molecular Weight: 179 g/mol

4-Cyano-1,1'-biphenyl (**9l**). This compound is known in the literature.[15]

Phenylboronic acid (146.4 mg, 1.2 mmol) was reacted with 4-iodobenzonitril (229.0 mg, 1.00 mmol), K_2CO_3 (276.41 mg, 2.00 mmol) and γ-Fe_2O_3@PEG@THMAM-Co **6** (0.6 mol%, 0.007 g) in water (4 mL) at 80 °C to yield 91% (162.8 mg) 4-cyano-1,1'-biphenyl (**9l**). Chromatography: *n*-hexane/EtOAc, 8:2. **^{1}H-NMR** ($CDCl_3$, 250 MHz) δ 7.44–7.77 (m, 9H). **^{13}C NMR** (75 MHz, $CDCl_3$) δ 111.3, 119.4, 127.7, 128.2, 129.1, 129.6, 133.1, 139.6, 146.1.

Phenylboronic acid (146.4 mg, 1.2 mmol) was reacted with 4-bromobenzonitril (182.0 mg, 1.00 mmol), K_2CO_3 (276.41 mg, 2.00 mmol) and γ-Fe_2O_3@PEG@THMAM-Co **6** (0.6 mol%, 0.007 g) in water (4 mL) at 80 °C to yield 80% (143.2 mg) 4-cyano-1,1'-biphenyl (**9l**). Chromatography: *n*-hexane/EtOAc, 8:2. **^{1}H-NMR** ($CDCl_3$, 250 MHz) δ 7.44–7.79 (m, 9H). **^{13}C NMR** (75 MHz, $CDCl_3$) δ 111.2, 119.4, 127.7, 128.5, 129.1, 129.6, 133.3, 139.5, 146.1.

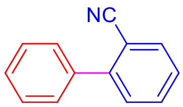

2-phenylbenzonitrile (9m)
Chemical Formula: $C_{13}H_9N$
Molecular Weight: 179.21 g/mol

2-Phenylbenzonitrile (**9m**). This compound is known in the literature.[20]

Phenylboronic acid (146.4 mg, 1.2 mmol) was reacted with 4-iodobenzonitril (229.0 mg, 1.00 mmol), K_2CO_3 (276.41 mg, 2.00 mmol) and γ-Fe_2O_3@PEG@THMAM-Co **6** (0.6 mol%, 0.007 g) in water (4 mL) at 80 °C to yield 90% (161.2 mg) [1,1'-biphenyl]-2-carbonitrile (**9m**). Chromatography: *n*-hexane/EtOAc, 8:2. **^{1}H-NMR** ($CDCl_3$, 300 MHz) δ 7.41–7.96 (m, 9H). **^{13}C NMR** (75 MHz) δ 111.2, 118.6, 127.5, 128.6, 128.7, 129.0, 130.0, 132.7, 133.6, 138.0, 145.4.

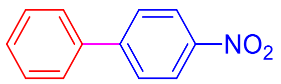

4-nitro-1,1'-biphenyl (9n)
Chemical Formula: $C_{12}H_9NO_2$
Molecular Weight: 199 g/mol

4-Nitro-1,1'-biphenyl (**9n**). This compound is known in the literature.[15]

Phenylboronic acid (146.4 mg, 1.2 mmol) was reacted with 1-iodo-4-nitrobenzene (249.0 mg, 1.00 mmol), K_2CO_3 (276.41 mg, 2.00 mmol) and γ-Fe_2O_3@PEG@THMAM-Co **6** (0.6 mol%, 0.007 g) in water (4 mL) at 80 °C to yield 94% (187.0 mg) 4-nitro-1,1'-biphenyl (**9n**). Chromatography: *n*-hexane/EtOAc, 8:2. **^{1}H-NMR** (DMSO, 300 MHz) δ 7.46–9.12 (m, 9H). **^{13}C NMR** (75 MHz, $CDCl_3$) δ 121.8, 124.6, 127.8, 128.3, 129.4, 129.6, 139.2, 148.1.

Phenylboronic acid (146.4 mg, 1.2 mmol) was reacted with 1-bromo-4-nitrobenzene (202.0 mg, 1.00 mmol), K_2CO_3 (276.41 mg, 2.00 mmol) and γ-Fe_2O_3@PEG@THMAM-Co **6** (0.6 mol%, 0.007 g) in water (4 mL) at 80 °C to yield 85% (169.1 mg) 4-nitro-1,1'-biphenyl (**9n**). Chromatography: *n*-hexane/EtOAc, 8:2. **^{1}H-NMR** (DMSO, 300 MHz) δ 7.44–9.10 (m, 9H). **^{13}C NMR** (75 MHz, $CDCl_3$) δ 121.9, 124.5, 127.5, 128.4, 129.3, 129.4, 139.2, 148.2.

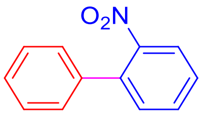

2-nitro-1,1'-biphenyl (9o)
Chemical Formula: $C_{12}H_9NO_2$
Molecular Weight: 199 g/mol

2-Nitro-1,1'-biphenyl (**9o**). This compound is known in the literature.[15]

Phenylboronic acid (146.4 mg, 1.2 mmol) was reacted with 1-iodo-2-nitrobenzene (249.0 mg, 1.00 mmol), K_2CO_3 (276.41 mg, 2.00 mmol) and γ-Fe_2O_3@PEG@THMAM-Co **6** (0.6 mol%, 0.007 g) in water (4 mL) at 80 °C to yield 85% (169.1 mg) 2-nitro-1,1'-biphenyl (**9o**). Chromatography: *n*-hexane/EtOAc, 8:2. **^{1}H-NMR** (DMSO, 300 MHz) δ 7.46–8.10 (m, 9H). **^{13}C NMR** (75 MHz, $CDCl_3$) δ 121.9, 122.0, 127.2, 128.6, 129.2, 129.8, 133.0, 136.7, 142.9, 148.8.

Phenylboronic acid (146.4 mg, 1.2 mmol) was reacted with 1-bromo-2-nitrobenzene (202.0 mg, 1.00 mmol), K_2CO_3 (276.41 mg, 2.00 mmol) and γ-Fe_2O_3@PEG@THMAM-Co **6** (0.6 mol%, 0.007 g) in water (4

mL) at 80 °C to yield 70% (139.3 mg) 2-nitro-1,1'-biphenyl (**9o**). Chromatography: *n*-hexane/EtOAc, 8:2. **^{1}H-NMR** (DMSO, 300 MHz) *δ* 7.46–8.11 (m, 9H). **^{13}C NMR** (75 MHz, $CDCl_3$) *δ* 121.8, 122.2, 127.4, 128.5, 129.3, 129.8, 133.1, 136.5, 142.9, 148.9.

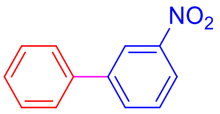

3-nitro-1,1'-biphenyl (9p)
Chemical Formula: $C_{12}H_9NO_2$
Molecular Weight: 199 g/mol

3-Nitro-1,1'-biphenyl (**9p**). This compound is known in the literature.[18]

Phenylboronic acid (146.4 mg, 1.2 mmol) was reacted with 1-iodo-3-nitrobenzene (249.0 mg, 1.00 mmol), K_2CO_3 (276.41 mg, 2.00 mmol) and γ-Fe_2O_3@PEG@THMAM-Co **6** (0.6 mol%, 0.007 g) in water (4 mL) at 80 °C to yield 80% (159.2 mg) 3-nitro-1,1'-biphenyl (**9p**). Chromatography: *n*-hexane/EtOAc, 8:2. **^{1}H-NMR** (DMSO, 300 MHz) *δ* 7.44–8.12 (m, 9H). **^{13}C NMR** (75 MHz, $CDCl_3$) *δ* 121.5, 122.6, 127.4, 129.0, 129.6, 130.9, 133.7, 138.2, 142.2, 148.8.

Phenylboronic acid (146.4 mg, 1.2 mmol) was reacted with 1-bromo-3-nitrobenzene (202.0 mg, 1.00 mmol), K_2CO_3 (276.41 mg, 2.00 mmol) and γ-Fe_2O_3@PEG@THMAM-Co **6** (0.6 mol%, 0.007 g) in water (4 mL) at 80 °C to yield 70% (139.3 mg) 3-nitro-1,1'-biphenyl (**9p**). Chromatography: *n*-hexane/EtOAc, 8:2. **^{1}H-NMR** (DMSO, 300 MHz) *δ* 7.44–8.11 (m, 9H). **^{13}C NMR** (75 MHz, $CDCl_3$) *δ* 121.9, 122.8, 127.2, 129.1, 129.6, 130.9, 133.7, 138.3, 142.2, 148.6.

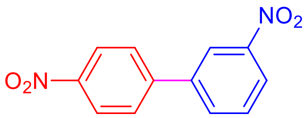

3,4'-dinitro-1,1'-biphenyl (9q)
Chemical Formula: $C_{12}H_8N_2O_4$
Molecular Weight: 244 g/mol

3,4'-Dinitro-1,1'-biphenyl (**9q**). This compound is known in the literature.[19]

(4-nitrophenyl)boronic acid (200.0 mg, 1.2 mmol) was reacted with 1-iodo-3-nitrobenzene (249.0 mg, 1.00 mmol), K_2CO_3 (276.41 mg, 2.00 mmol) and γ-Fe_2O_3@PEG@THMAM-Co **6** (0.6 mol%, 0.007 g) in water (4 mL) at 80 °C to yield 85% (207.4 mg) 3,4'-dinitro-1,1'-biphenyl (**9q**). Chromatography: *n*-hexane/EtOAc,

8:2. ^{1}**H-NMR** ($CDCl_3$, 300 MHz) δ 7.76–8.63 (m, 8H). ^{13}**C NMR** (75 MHz, $CDCl_3$) δ 123.3, 123.5, 124.4, 128.1, 130.2, 133.0, 133.2, 140.3, 140.4, 144.8.

(4-nitrophenyl)boronic acid (200.0 mg, 1.2 mmol) was reacted with 1-bromo-3-nitrobenzene (202.0 mg, 1.00 mmol), K_2CO_3 (276.41 mg, 2.00 mmol) and γ-Fe_2O_3@PEG@THMAM-Co **6** (0.6 mol%, 0.007 g) in water (4 mL) at 80 °C to yield 70% (170.8 mg) 3,4'-dinitro-1,1'-biphenyl (**9q**). Chromatography: *n*-hexane/EtOAc, 8:2. ^{1}**H-NMR** ($CDCl_3$, 300 MHz) δ 7.73–8.51 (m, 8H). ^{13}**C NMR** (75 MHz, $CDCl_3$) δ 123.3, 123.8, 124.7, 128.6, 130.1, 133.0, 133.2, 140.8, 141.0, 144.8.

Data of Hiyama Products

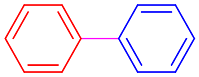

biphenyl (11a)
Chemical Formula: $C_{12}H_{10}$
Molecular Weight: 154.21 g/mol

Biphenyl (**11a**). This compound is known in the literature.[15]

Triethoxyphenylsilane (360.0 mg, 1.5 mmol) was reacted with iodobenzene (204.0 mg, 1.00 mmol), NaOH (80.0 mg, 2.00 mmol) and γ-Fe_2O_3@ γ-Fe_2O_3@PEG@THMAM-Co **6** (0.6 mol%, 0.007 g) in water (4 mL) at 80 °C to yield 93% (143.4 mg) biphenyl (**11a**). Chromatography: *n*-hexane/EtOAc, 8:2. ^{1}**H-NMR** ($CDCl_3$, 300 MHz) δ 7.35–7.68 (m, 9H). ^{13}**C NMR** (75 MHz, $CDCl_3$): δ 127.2, 128.6, 129.4, 141.3.

Triethoxyphenylsilane (360.0 mg, 1.5 mmol) was reacted with bromobenzene (157.0 mg, 1.00 mmol), NaOH (80.0 mg, 2.00 mmol) and γ-Fe_2O_3@ γ-Fe_2O_3@PEG@THMAM-Co **6** (0.6 mol%, 0.007 g) in water (4 mL) at 80 °C to yield 85% (131.0 mg) biphenyl (**11a**). Chromatography: *n*-hexane/EtOAc, 8:2. ^{1}**H-NMR** ($CDCl_3$, 300 MHz) δ 7.38–7.65 (m, 9H). ^{13}**C NMR** (75 MHz, $CDCl_3$): δ 127.1, 128.8, 129.0, 141.3.

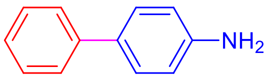

4-amine-1,1'-biphenyl (11b)
Chemical Formula: $C_{12}H_{11}N$
Molecular Weight: 169 g/mol

4-Amine-1,1'-biphenyl (**11b**). This compound is known in the literature.[15]

Triethoxyphenylsilane (360.0 mg, 1.5 mmol) was reacted with 4-iodoaniline (219.0 mg, 1.00 mmol), NaOH (80.0 mg, 2.00 mmol) and γ-Fe_2O_3@PEG@THMAM-Co **6** (0.6 mol%, 0.007 g) in water (4 mL) at 80 °C to yield 85% (143.6 mg) 4-amine-1,1'-biphenyl (**11b**). Chromatography: *n*-hexane/EtOAc, 8:2. ^{1}**H-NMR** (DMSO, 250 MHz) δ 5.26 (s, 2H, NH_2), 6.68–7.57 (m, 9H, Ar*H*). ^{13}**C NMR** (75 MHz, $CDCl_3$) δ 114.6, 125.8, 126.6, 127.5, 127.8, 129.2, 141.3, 148.9.

Triethoxyphenylsilane (360.0 mg, 1.5 mmol) was reacted with 4-bromoaniline (172.0 mg, 1.00 mmol), NaOH (80.0 mg, 2.00 mmol) and γ-Fe_2O_3@PEG@THMAM-Co **6** (0.6 mol%, 0.007 g) in water (4 mL) at 80 °C to yield 70% (118.3 mg) 4-amine-1,1'-biphenyl (**11b**). Chromatography: *n*-hexane/EtOAc, 8:2. ^{1}**H-NMR** (DMSO, 250 MHz) δ 5.25 (s, 2H, NH_2), 6.65–7.54 (m, 9H, Ar*H*). ^{13}**C NMR** (75 MHz, $CDCl_3$) δ 114.5, 125.6, 126.4, 127.6, 127.9, 129.3, 141.1, 148.9.

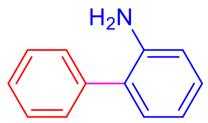

[1,1'-biphenyl]-2-amine (11c)
Chemical Formula: $C_{12}H_{11}N$
Molecular Weight: 169.21 g/mol

[1,1'-Biphenyl]-2-amine (**11c**). This compound is known in the literature.[15]

Triethoxyphenylsilane (360.0 mg, 1.5 mmol) was reacted with 2-iodoaniline (219.0 mg, 1.00 mmol), NaOH (80.0 mg, 2.00 mmol) and γ-Fe_2O_3@PEG@THMAM-Co **6** (0.6 mol%, 0.007 g) in water (4 mL) at 80 °C to yield 80% (135.3 mg) [1,1'-biphenyl]-2-amine (**11c**). Chromatography: *n*-hexane/EtOAc, 8:2. ^{1}**H-NMR** ($CDCl_3$, 250 MHz) δ 4.79 (s, 2H), 6.64–7.53 (m, 9H). ^{13}**C NMR** (75 MHz, $CDCl_3$) δ 115.4, 117.0, 126.1, 127.2, 128.8, 129.3, 129.7, 130.4, 140.2, 145.4.

Triethoxyphenylsilane (360.0 mg, 1.5 mmol) was reacted with 2-bromoaniline (172.0 mg, 1.00 mmol), NaOH (80.0 mg, 2.00 mmol) and γ-Fe_2O_3@PEG@THMAM-Co **6** (0.6 mol%, 0.007 g) in water (4 mL) at 80 °C to yield 75% (126.9 mg) [1,1'-biphenyl]-2-amine (**11c**). Chromatography: *n*-hexane/EtOAc, 8:2. ^{1}**H**-NMR ($CDCl_3$, 250 MHz) δ 4.78 (s, 2H), 6.65–7.51 (m, 9H). ^{13}**C NMR** (75 MHz, $CDCl_3$) δ 115.3, 117.2, 126.2, 127.1, 128.5, 129.1, 129.5, 130.3, 140.2, 145.5.

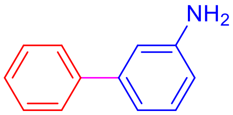

3-amine-1,1'-biphenyl (11d)
Chemical Formula: $C_{12}H_{11}N$
Molecular Weight: 169 g/mol

3-Amine-1,1'-biphenyl (**11d**). This compound is known in the literature.[15]

Triethoxyphenylsilane (360.0 mg, 1.5 mmol) was reacted with 3-iodoaniline (219.0 mg, 1.00 mmol), NaOH (80.0 mg, 2.00 mmol) and γ-Fe_2O_3@PEG@THMAM-Co **6** (0.6 mol%, 0.007 g) in water (4 mL) at 80 °C to yield 70% (118.3 mg) 3-amine-1,1'-biphenyl (**11d**). Chromatography: *n*-hexane/EtOAc, 8:2. **^{1}H-NMR** (DMSO, 250 MHz) *δ* 4.73 (s, 2H), 6.60–7.58 (m, 9H). **^{13}C NMR** (75 MHz, $CDCl_3$) *δ* 114.4, 118.0, 118.9, 127.7, 127.9, 129.0, 130.8, 140.7, 140.9, 149.4.

Triethoxyphenylsilane (360.0 mg, 1.5 mmol) was reacted with 3-bromoaniline (172.0 mg, 1.00 mmol), NaOH (80.0 mg, 2.00 mmol) and γ-Fe_2O_3@PEG@THMAM-Co **6** (0.6 mol%, 0.007 g) in water (4 mL) at 80 °C to yield 60% (101.4 mg) 3-amine-1,1'-biphenyl (**11d**). Chromatography: *n*-hexane/EtOAc, 8:2. **^{1}H-NMR** (DMSO, 250 MHz) *δ* 4.75 (s, 2H), 6.57–7.54 (m, 9H). **^{13}C NMR** (75 MHz, $CDCl_3$) *δ* 114.1, 118.3, 118.9, 129.4, 127.5, 127.9, 130.9, 140.2, 140.8, 149.5.

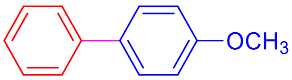

4-methoxy-1,1'-biphenyl (11e)
Chemical Formula: $C_{13}H_{12}O$
Molecular Weight: 184 g/mol

4-Methoxy-1,1'-biphenyl (**11e**). This compound is known in the literature.[16]

Triethoxyphenylsilane (360.0 mg, 1.5 mmol) was reacted with 1-iodo-4-methoxybenzene (234.0 mg, 1.00 mmol), NaOH (80.0 mg, 2.00 mmol) and γ-Fe_2O_3@PEG@THMAM-Co **6** (0.6 mol%, 0.007 g) in water (4 mL) at 80 °C to yield 90% (165.6 mg) 4-methoxy-1,1'-biphenyl (**11e**). Chromatography: *n*-hexane/EtOAc, 8:2. **^{1}H-NMR** ($CDCl_3$, 300 MHz) *δ* 2.91 (s, 3H), 7.02–7.64 (m, 9H, Ar*H*). **^{13}C NMR** (75 MHz, $CDCl_3$) *δ* 55.7, 114.5, 127.0, 127.6, 128.6, 129.2, 134.1, 141.4, 159.6.

Triethoxyphenylsilane (360.0 mg, 1.5 mmol) was reacted with 1-bromo-4-methoxybenzene (187.0 mg, 1.00 mmol), NaOH (80.0 mg, 2.00 mmol) and γ-Fe_2O_3@PEG@THMAM-Co **6** (0.6 mol%, 0.007 g) in water

(4 mL) at 80 °C to yield 80% (147.2 mg) 4-methoxy-1,1'-biphenyl (**11e**). Chromatography: *n*-hexane/EtOAc, 8:2. **^{1}H-NMR** ($CDCl_3$, 300 MHz) δ 2.90 (s, 3H), 7.05–7.63 (m, 9H, Ar*H*). **^{13}C NMR** (75 MHz, $CDCl_3$) δ 55.8, 114.6, 127.1, 127.3, 128.6, 129.0, 134.5, 141.3, 159.8.

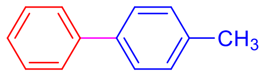

4-methyl-1,1'-biphenyl (11f)
Chemical Formula: $C_{13}H_{12}$
Molecular Weight: 168 g/mol

4-Methyl-1,1'-biphenyl (**11f**). This compound is known in the literature.[15]

Triethoxyphenylsilane (360.0 mg, 1.5 mmol) was reacted with 1-iodo-4-methylbenzene (218.0 mg, 1.00 mmol), NaOH (80.0 mg, 2.00 mmol) and γ-Fe_2O_3@PEG@THMAM-Co **6** (0.6 mol%, 0.007 g) in water (4 mL) at 80 °C to yield 88% (147.8 mg) 4-methyl-1,1'-biphenyl (**11f**). Chromatography: *n*-hexane/EtOAc, 8:2. **^{1}H-NMR** ($CDCl_3$, 300 MHz) δ 2.47 (s, 3H), 7.27–7.67 (m, 9H). **^{13}C NMR** (75 MHz, $CDCl_3$) δ 21.6, 127.5, 127.5, 127.9, 129.0, 130.2, 137.7, 138.9, 141.8.

Triethoxyphenylsilane (360.0 mg, 1.5 mmol) was reacted with 1-bromo-4-methylbenzene (171.0 mg, 1.00 mmol), NaOH (80.0 mg, 2.00 mmol) and γ-Fe_2O_3@PEG@THMAM-Co **6** (0.6 mol%, 0.007 g) in water (4 mL) at 80 °C to yield 70% (117.6 mg) 4-methyl-1,1'-biphenyl (**11f**). Chromatography: *n*-hexane/EtOAc, 8:2. **^{1}H-NMR** ($CDCl_3$, 300 MHz) δ 2.44 (s, 3H), 7.25–7.69 (m, 9H). **^{13}C NMR** (75 MHz, $CDCl_3$) δ 21.5, 127.5, 127.7, 127.8, 129.2, 130.0, 137.6, 138.8, 141.8.

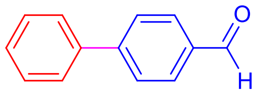

[1,1'-biphenyl]-4-carbaldehyde (11g)
Chemical Formula: $C_{13}H_{10}O$
Molecular Weight: 182 g/mol

[1,1'-Biphenyl]-4-carbaldehyde (**11g**). This compound is known in the literature.[15]

Triethoxyphenylsilane (360.0 mg, 1.5 mmol) was reacted with 4-iodobenzaldehyde (232.0 mg, 1.00 mmol), NaOH (80.0 mg, 2.00 mmol) and γ-Fe_2O_3@PEG@THMAM-Co **6** (0.6 mol%, 0.007 g) in water (4 mL) at 80 °C to yield 94% (171.0 mg) [1,1'-biphenyl]-4-carbaldehyde (**11g**). Chromatography: *n*-hexane/EtOAc, 8:2. **^{1}H-NMR** ($CDCl_3$, 300 MHz) δ 7.44–8.25 (m, 9H), 10.15 (s, 1H). **^{13}C NMR** (75 MHz, $CDCl_3$) δ 127.7, 128.9, 129.5, 129.9, 130.8, 135.6, 140.0, 147.7, 192.0.

Triethoxyphenylsilane (360.0 mg, 1.5 mmol) was reacted with 4-bromobenzaldehyde (185.0 mg, 1.00 mmol), NaOH (80.0 mg, 2.00 mmol) and γ-Fe_2O_3@PEG@THMAM-Co **6** (0.6 mol%, 0.007 g) in water (4 mL) at 80 °C to yield 85% (154.7 mg) [1,1'-biphenyl]-4-carbaldehyde (**11g**). Chromatography: *n*-hexane/EtOAc, 8:2. **^{1}H-NMR** ($CDCl_3$, 300 MHz) δ 7.43–8.21 (m, 9H), 10.13 (s, 1H). **^{13}C NMR** (75 MHz, $CDCl_3$) δ 127.5, 128.9, 129.5, 129.6, 130.7, 135.5, 140.2, 147.8, 192.4.

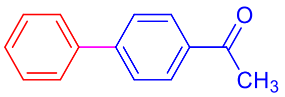

1-([1,1'-biphenyl]-4-yl)ethan-1-one (11h)
Chemical Formula: $C_{14}H_{12}O$
Molecular Weight: 196 g/mol

[1,1'-Biphenyl]-4-carbaldehyde (**11h**). This compound is known in the literature.[18]

Triethoxyphenylsilane (360.0 mg, 1.5 mmol) was reacted with 1-(4-iodophenyl)ethanone (246.0 mg, 1.00 mmol), NaOH (80.0 mg, 2.00 mmol) and γ-Fe_2O_3@PEG@THMAM-Co **6** (0.6 mol%, 0.007 g) in water (4 mL) at 80 °C to yield 92% (180.3 mg) [1,1'-biphenyl]-4-carbaldehyde (**11h**). Chromatography: *n*-hexane/EtOAc, 8:2. **^{1}H-NMR** ($CDCl_3$, 300 MHz) δ 2.72 (s, 3H), 7.31–8.12 (m, 9H). **^{13}C NMR** (75 MHz, $CDCl_3$) δ 26.6, 127.22, 127.29, 128.1, 128.7, 130.1, 135.8, 139.6, 145.5, 197.9.

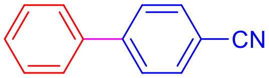

4-cyano-1,1'-biphenyl (11i)
Chemical Formula: $C_{13}H_9N$
Molecular Weight: 179 g/mol

4-Cyano-1,1'-biphenyl (**11i**). This compound is known in the literature.[15]

Triethoxyphenylsilane (360.0 mg, 1.5 mmol) was reacted with 4-iodobenzonitril (229.0 mg, 1.00 mmol), NaOH (80.0 mg, 2.00 mmol) and γ-Fe_2O_3@PEG@THMAM-Co **6** (0.6 mol%, 0.007 g) in water (4 mL) at 80 °C to yield 89% (159.3 mg) 4-Cyano-1,1'-biphenyl (**11i**). Chromatography: *n*-hexane/EtOAc, 8:2. **^{1}H-NMR** ($CDCl_3$, 250 MHz) δ 7.43–7.75 (m, 9H). **^{13}C NMR** (75 MHz, $CDCl_3$) δ 111.2, 119.0, 127.4, 128.2, 129.0, 129.3, 133.1, 139.6, 146.2.

Triethoxyphenylsilane (360.0 mg, 1.5 mmol) was reacted with 4-bromobenzonitril (182.0 mg, 1.00 mmol), NaOH (80.0 mg, 2.00 mmol) and γ-Fe_2O_3@PEG@THMAM-Co **6** (0.6 mol%, 0.007 g) in water (4 mL) at 80

°C to yield 75% (134.2 mg) 4-Cyano-1,1'-biphenyl (**11i**). Chromatography: *n*-hexane/EtOAc, 8:2. ^{1}**H-NMR** ($CDCl_3$, 250 MHz) *δ* 7.44–7.78 (m, 9H). ^{13}**C NMR** (75 MHz, $CDCl_3$) *δ* 111.1, 119.4, 127.7, 128.2, 129.3, 129.5, 133.1, 139.6, 146.0.

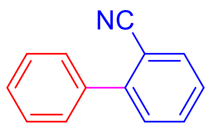

2-cyano-1,1'-biphenyl (11j)
Chemical Formula: $C_{13}H_9N$
Molecular Weight: 179 g/mol

2-Cyano-1,1'-biphenyl (**11j**). This compound is known in the literature.[15]

Triethoxyphenylsilane (360.0 mg, 1.5 mmol) was reacted with 4-iodobenzonitril (229.0 mg, 1.00 mmol), NaOH (80.0 mg, 2.00 mmol) and γ-Fe_2O_3@PEG@THMAM-Co **6** (0.6 mol%, 0.007 g) in water (4 mL) at 80 °C to yield 89% (161.0 mg) 2-Cyano-1,1'-biphenyl (**11j**). Chromatography: *n*-hexane/EtOAc, 8:2. ^{1}**H-NMR** ($CDCl_3$, 250 MHz) *δ* 7.43–7.75 (m, 9H). ^{13}**C NMR** (75 MHz, $CDCl_3$) *δ* 111.2, 119.0, 127.4, 128.2, 129.0, 129.3, 133.1, 139.6, 146.2.

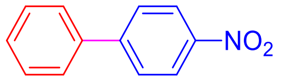

4-nitro-1,1'-biphenyl (11k)
Chemical Formula: $C_{12}H_9NO_2$
Molecular Weight: 199 g/mol

4-Nitro-1,1'-biphenyl (**11k**). This compound is known in the literature.[15]

Triethoxyphenylsilane (360.0 mg, 1.5 mmol) was reacted with 1-iodo-4-nitrobenzene (249.0 mg, 1.00 mmol), NaOH (80.0 mg, 2.00 mmol) and γ-Fe_2O_3@PEG@THMAM-Co **6** (0.6 mol%, 0.007 g) in water (4 mL) at 80 °C to yield 95% (189.0 mg) 4-nitro-1,1'-biphenyl (**11k**). Chromatography: *n*-hexane/EtOAc, 8:2. ^{1}**H-NMR** ($CDCl_3$, 300 MHz) *δ* 7.43–9.10 (m, 9H). ^{13}**C NMR** (75 MHz, $CDCl_3$) *δ* 121.9, 124.4, 127.5, 128.41 129.3, 129.6, 139.2, 148.0.

Triethoxyphenylsilane (360.0 mg, 1.5 mmol) was reacted with 1-bromo-4-nitrobenzene (202.0 mg, 1.00 mmol), NaOH (80.0 mg, 2.00 mmol) and γ-Fe_2O_3@PEG@THMAM-Co **6** (0.6 mol%, 0.007 g) in water (4 mL) at 80 °C to yield 84% (167.1 mg) 4-nitro-1,1'-biphenyl (**11k**). Chromatography: *n*-hexane/EtOAc, 8:2. ^{1}**H-**

NMR ($CDCl_3$, 300 MHz) δ 7.46–9.08 (m, 9H). **^{13}C NMR** (75 MHz, $CDCl_3$) δ 121.9, 124.9, 127.5, 128.2, 129.4, 129.5, 139.1, 148.8.

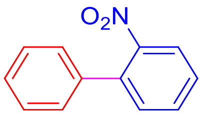

2-nitro-1,1'-biphenyl (11m)
Chemical Formula: $C_{12}H_9NO_2$
Molecular Weight: 199 g/mol

2-Nitro-1,1'-biphenyl (**11l**). This compound is known in the literature.[15]

Triethoxyphenylsilane (360.0 mg, 1.5 mmol) was reacted with 1-iodo-2-nitrobenzene (249.0 mg, 1.00 mmol), NaOH (80.0 mg, 2.00 mmol) and γ-Fe_2O_3@PEG@THMAM-Co **6** (0.6 mol%, 0.007 g) in water (4 mL) at 80 °C to yield 84% (167.1 mg) 2-nitro-1,1'-biphenyl (**11m**). Chromatography: *n*-hexane/EtOAc, 8:2. **^{1}H-NMR** (DMSO, 300 MHz) δ 7.46–8.15 (m, 9H). **^{13}C NMR** (75 MHz, $CDCl_3$) δ 121.8, 122.1, 127.2, 128.6, 129.2, 129.6, 133.0, 136.7, 142.9, 148.9.

Triethoxyphenylsilane (360.0 mg, 1.5 mmol) was reacted with 1-bromo-2-nitrobenzene (202.0 mg, 1.00 mmol), NaOH (80.0 mg, 2.00 mmol) and γ-Fe_2O_3@PEG@THMAM-Co **6** (0.6 mol%, 0.007 g) in water (4 mL) at 80 °C to yield 75% (149.2 mg) 2-nitro-1,1'-biphenyl (**11m**). Chromatography: *n*-hexane/EtOAc, 8:2. **^{1}H-NMR** (DMSO, 300 MHz) δ 7.49–8.13 (m, 9H). **^{13}C NMR** (75 MHz, $CDCl_3$) δ 121.9, 122.0, 127.3, 128.5, 129.0, 129.8, 133.2, 136.6, 142.9, 148.5.

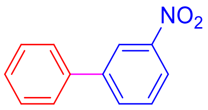

3-nitro-1,1'-biphenyl (11l)
Chemical Formula: $C_{12}H_9NO_2$
Molecular Weight: 199 g/mol

3-Nitro-1,1'-biphenyl (**11m**). This compound is known in the literature.[18]

Triethoxyphenylsilane (360.0 mg, 1.5 mmol) was reacted with 1-iodo-3-nitrobenzene (249.0 mg, 1.00 mmol), NaOH (80.0 mg, 2.00 mmol) and γ-Fe_2O_3@PEG@THMAM-Co **6** (0.6 mol%, 0.007 g) in water (4 mL) at 80 °C to yield 85% (169.1 mg) 3-nitro-1,1'-biphenyl (**11l**). Chromatography: *n*-hexane/EtOAc, 8:2. **^{1}H-NMR** ($CDCl_3$, 300 MHz) δ 7.44–8.13 (m, 9H). **^{13}C NMR** (75 MHz, $CDCl_3$) δ 121.7, 122.8, 127.5, 129.1, 129.8, 130.9, 133.7, 138.2, 142.1, 148.8.

Data of C-N coupling Products

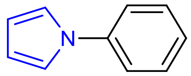

1-phenyl-1*H*-pyrrole (**13a**)
Chemical Formula: $C_{10}H_9N$
Molecular Weight: 143 g/mol

1-Phenyl-1*H*-pyrrole (13a). This compound is known in the literature.[15]

Phenylboronic acid (121.9 mg, 1.0 mmol) was reacted with 1*H*-pyrrole (101.8 mg, 1.5 mmol), NaOH (80.0 mg, 2.00 mmol) and γ-Fe_2O_3@PEG@THMAM-Co **6** (3 mol%, 0.035 g) in water (4 mL) at 100 °C to yield 85% (121.5 mg) 1-phenyl-1*H*-pyrrole (1**3a**). Chromatography: *n*-hexane/EtOAc, 7:3. **^{1}H NMR** (300 MHz, $CDCl_3$) *δ* (ppm) 6.41 (t, 2H), 7.11 (m, 2H), 7.35–7.46 (m, 5H). **^{13}C-NMR** (75 MHz, $CDCl_3$) *δ* 110.3, 120.4, 121.5, 126.3, 130.8, 140.9.

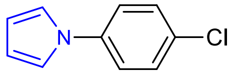

1-(4-chlorophenyl)-1*H*-pyrrole (**13b**)
Chemical Formula: $C_{10}H_8ClN$
Molecular Weight: 177.63 g/mol

1-(4-Chlorophenyl)-1*H*-pyrrole (**13b**)**.** This compound is known in the literature.[21]

4-Cl-phenylboronic acid (156.3 mg, 1.0 mmol) was reacted with 1*H*-pyrrole (101.8 mg, 1.5 mmol), NaOH (80.0 mg, 2.00 mmol) and γ-Fe_2O_3@PEG@THMAM-Co **6** (3 mol%, 0.035 g) in water (4 mL) at 100 °C to yield 90% (159.8 mg) 1-(4-chlorophenyl)-1*H*-pyrrole (**13b**). Chromatography: *n*-hexane/EtOAc, 7:3. **^{1}H-NMR** ($CDCl_3$, 300 MHz) *δ* 6.42 (s, 2H), 7.11 (s, 2H), 7.30–7.47 (m, 4H). **^{13}C NMR** (75 MHz, $CDCl_3$) *δ* 110.8, 119.3, 121.6, 129.6, 131.0, 139.3.

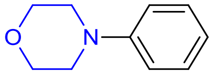

4-phenylmorpholine (**13c**)
Chemical Formula: $C_{10}H_{13}NO$
Molecular Weight: 163 g/mol

4-Phenylmorpholine (1**3c**). This compound is known in the literature.[15]

Phenylboronic acid (121.9 mg, 1.0 mmol) was reacted with morpholine (130.6 mg, 1.5 mmol), NaOH (80.0 mg, 2.00 mmol) and γ-Fe_2O_3@PEG@THMAM-Co **6** (3 mol%, 0.035 g) in water (4 mL) at 100 °C to yield 80% (130.4 mg) 4-phenylmorpholine (1**3c**). Chromatography: *n*-hexane/EtOAc, 7:3. ^{1}**H NMR** (300 MHz, DMSO) δ (ppm) 3.09 (t, 4H), 3.13 (t, 4H), 6.81–7.29 (m, 5H). ^{13}**C NMR** (75 MHz, $CDCl_3$) δ 48.9, 66.6, 115.5, 119.6, 129.4, 151.5.

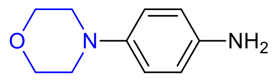

4-morpholinoaniline (**13d**)
Chemical Formula: $C_{10}H_{14}N_2O$
Molecular Weight: 178.23 g/mol

4-Morpholinoaniline (1**3d**)**.**

4-Amino-phenylboronic acid (136.9 mg, 1.0 mmol) was reacted with morpholine (130.6 mg, 1.5 mmol), NaOH (80.0 mg, 2.00 mmol) and γ-Fe_2O_3@PEG@THMAM-Co **6** (3 mol%, 0.035 g) in water (4 mL) at 100 °C to yield 75% (133.6 mg) 4-morpholinoaniline (**13d**). Chromatography: *n*-hexane/EtOAc, 7:3. ^{1}**H NMR** (300 MHz, DMSO) δ 2.89 (t, 4H), 3.71 (t, 4H), 6.50–6.75 (m, 5H). ^{13}**C NMR** (75 MHz, $CDCl_3$) δ 51.1, 66.8, 115.2, 118.0, 142.7, 142.8.

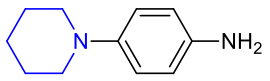

4-(piperidin-1-yl)aniline (**13e**)
Chemical Formula: $C_{11}H_{16}N_2$
Molecular Weight: 176 g/mol

4-(Piperidin-1-yl)aniline (1**3e**).

4-Amino-phenylboronic acid (136.9 mg, 1.0 mmol) was reacted with piperidine (127.7 mg, 1.5 mmol), NaOH (80.0 mg, 2.00 mmol) and γ-Fe_2O_3@PEG@THMAM-Co **6** (3 mol%, 0.035 g) in water (4 mL) at 100 °C to yield 72% (126.7 mg) 4-(piperidin-1-yl)aniline (1**3e**). Chromatography: *n*-hexane/EtOAc, 7:3. ^{1}**H NMR** (300 MHz, $CDCl_3$) δ 2.65 (m, 6H), 3.12 (m, 4H), 6.65 (s, 2H), 6.85–7.01 (m, 4H). ^{13}**C NMR** ($CDCl_3$, 75 MHz) δ 24.3, 26.2, 52.6, 116.1, 119.1, 139.8, 145.8.

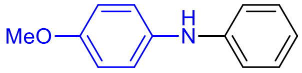

4-methoxy-*N*-phenylaniline (13f)
Chemical Formula: $C_{13}H_{13}NO$
Molecular Weight: 199.25 g/mol

4-Methoxy-*N*-phenylaniline (**13f**). This compound is known in the literature.[22]

Phenylboronic acid (121.9 mg, 1.0 mmol) was reacted with 4-methoxyaniline (184.7 mg, 1.5 mmol), NaOH (80.0 mg, 2.00 mmol) and γ-Fe_2O_3@PEG@THMAM-Co **6** (3 mol%, 0.035 g) in water (4 mL) at 100 °C to yield 90% (179.3 mg) 4-methoxy-N-phenylaniline (**13f**). Chromatography: *n*-hexane/EtOAc, 7:3. **^{1}H NMR** (400 MHz, Acetone-d_6) δ 7.19–6.85 (m, 9H), 5.90 (s, 1H), 3.78 (s, 3H). **^{13}C NMR** (75 MHz, $CDCl_3$) δ 55.4, 115.2, 121.6, 121.9, 125.3, 129.5, 134.7, 142.9, 152.7.

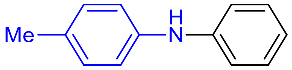

4-methyl-*N*-phenylaniline (13g)
Chemical Formula: $C_{13}H_{13}N$
Molecular Weight: 183 g/mol

4-Methyl-*N*-phenylaniline (**13g**). This compound is known in the literature.[22]

Phenylboronic acid (121.9 mg, 1.0 mmol) was reacted with 4-methylaniline (160.7 mg, 1.5 mmol), NaOH (80.0 mg, 2.00 mmol) and γ-Fe_2O_3@PEG@THMAM-Co **6** (3 mol%, 0.035 g) in water (4 mL) at 100 °C to yield 84% (153.7 mg) 4-methyl-*N*-phenylaniline (**13g**). Chromatography: *n*-hexane/EtOAc, 7:3. **^{1}H NMR** (400 MHz, $CDCl_3$) δ 6.92–7.30 (m, 9H), 5.80–5.60 (s, 1H), 2.35 (s, 3H). **^{13}C NMR** (75 MHz, $CDCl_3$) δ 20.2, 116.1, 118.4, 120.3, 129.2, 129.8, 131.0, 140.5, 144.3.

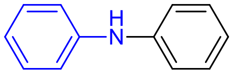

diphenylamine (13h)
Chemical Formula: $C_{12}H_{11}N$
Molecular Weight: 169 g/mol

Diphenylamine (**13h**)**.** This compound is known in the literature.[15]

Phenylboronic acid (121.9 mg, 1.0 mmol) was reacted with aniline (139.6 mg, 1.5 mmol), NaOH (80.0 mg, 2.00 mmol) and γ-Fe_2O_3@PEG@THMAM-Co **6** (3 mol%, 0.035 g) in water (4 mL) at 100 °C to yield 90% (152.1 mg) diphenylamine (**13h**). Chromatography: *n*-hexane/EtOAc, 7:3. **^{1}H NMR** (250 MHz, $CDCl_3$) δ 5.64 (s, 1H), 6.95–7.26 (m, 10H). **^{13}C NMR** (75 MHz, $CDCl_3$) δ 117.7, 121.2, 130.3, 145.8.

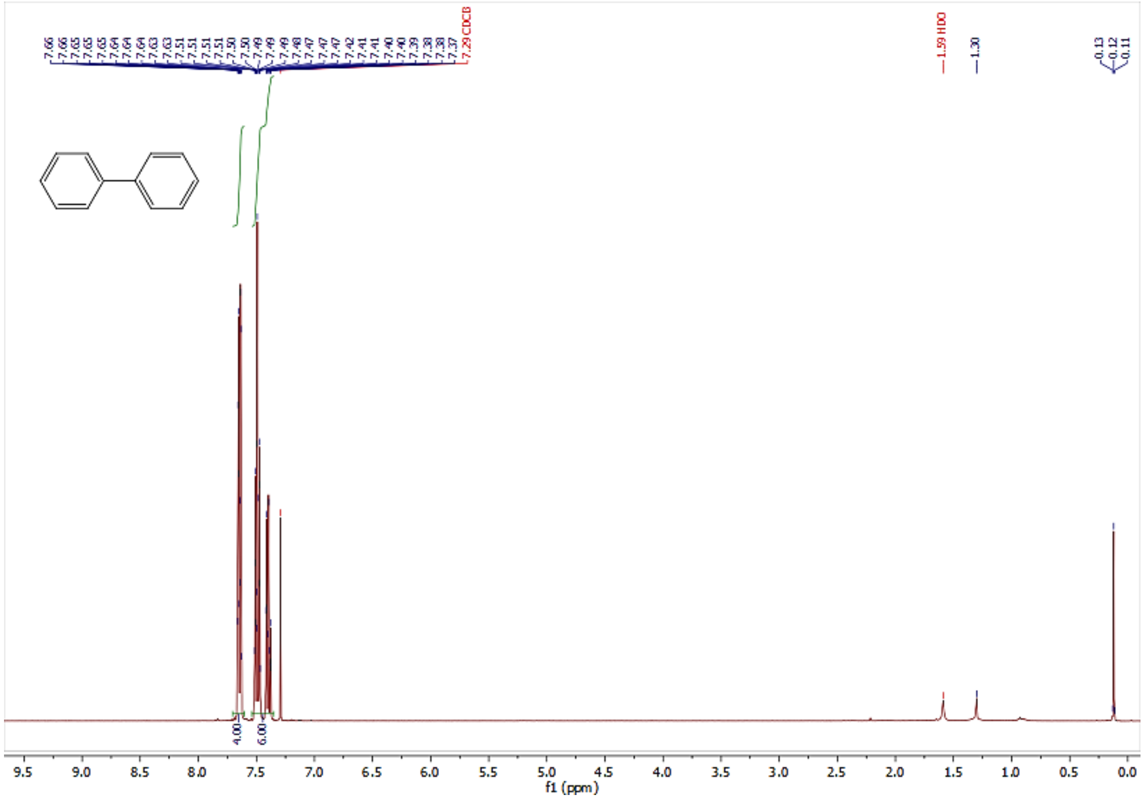
7.29 CDCl3
1.59 HDO
1.30
4.00
6.00
f1 (ppm)

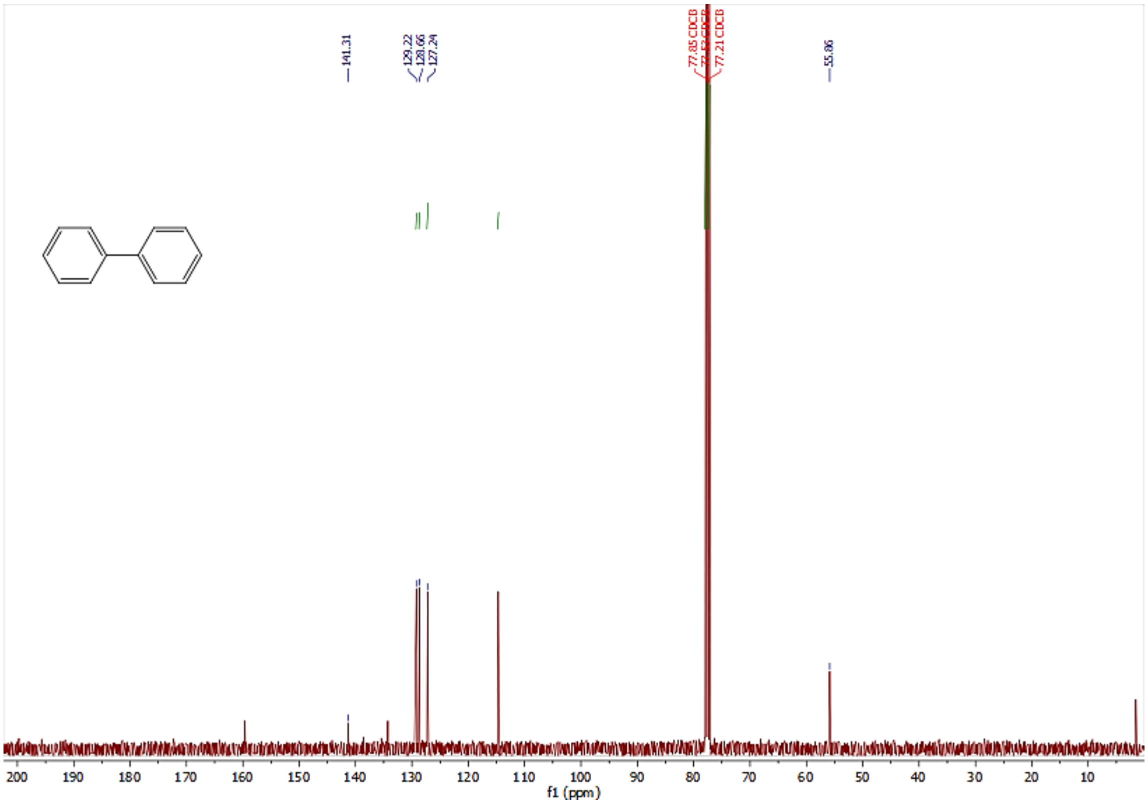
141.31
129.22
128.66
127.24
77.85 CDCl3
77.21 CDCl3
55.86
f1 (ppm)

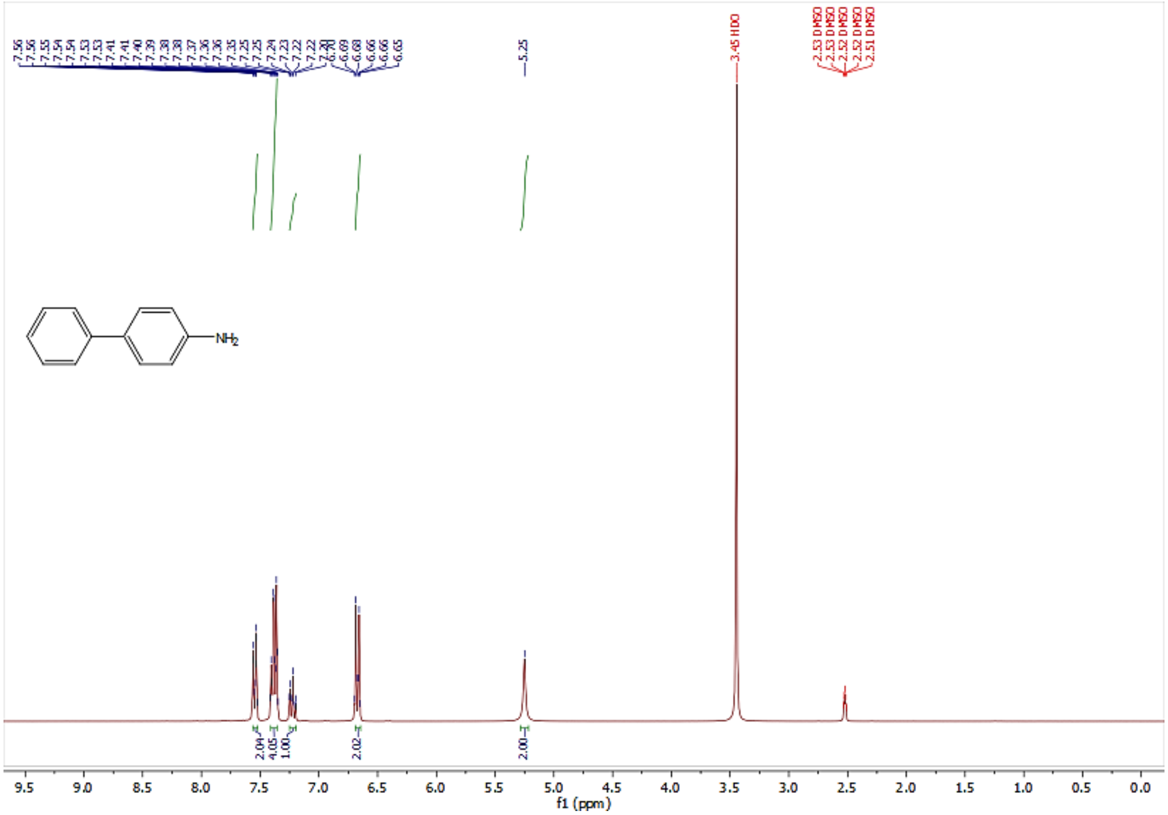
NH2
7.56
7.56
7.55
7.54
7.54
7.53
7.53
7.41
7.41
7.40
7.39
7.38
7.38
7.37
7.36
7.36
7.35
7.25
7.25
7.24
7.23
7.22
7.22
6.69
6.68
6.66
6.66
6.65
5.25
3.45 HDO
2.53 DMSO
2.53 DMSO
2.52 DMSO
2.52 DMSO
2.51 DMSO
2.04
4.05
1.00
2.02
2.00
f1 (ppm)

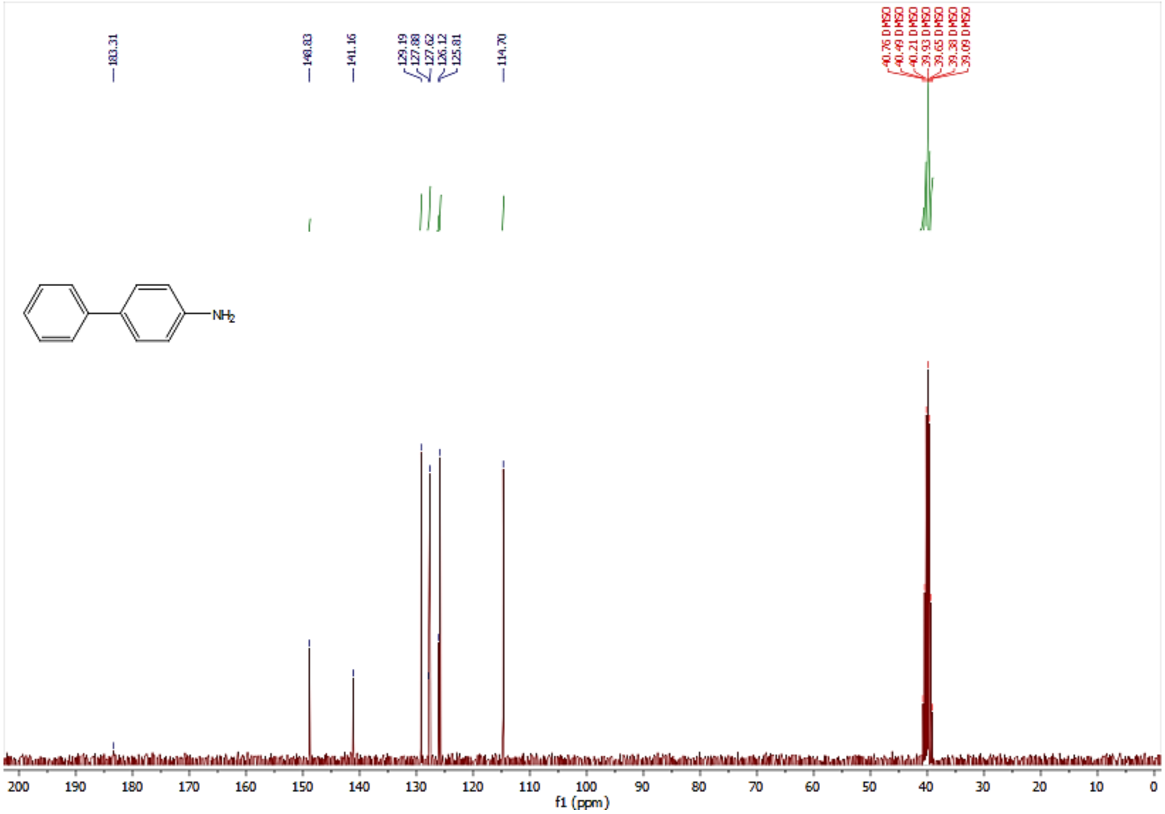
NH2
183.31
148.83
141.16
129.19
127.88
127.62
126.12
125.81
114.70
40.76 DMSO
40.49 DMSO
40.21 DMSO
39.93 DMSO
39.65 DMSO
39.38 DMSO
39.09 DMSO
f1 (ppm)

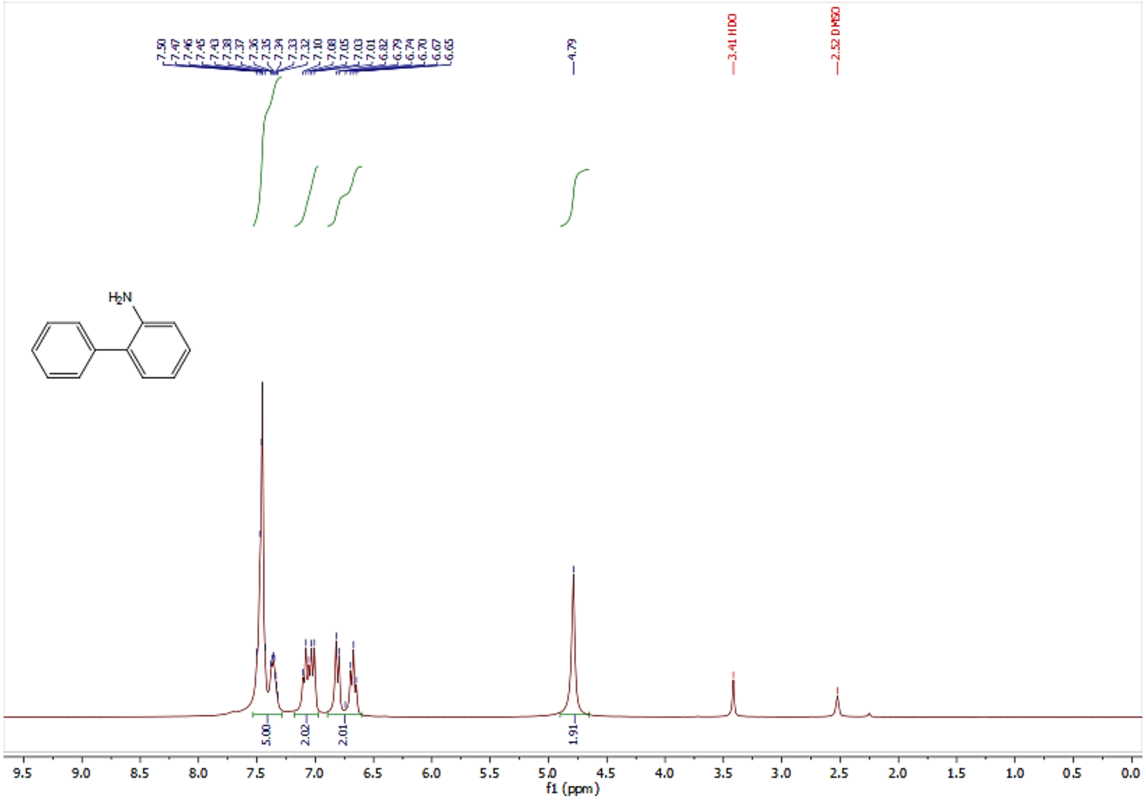
H2N
7.50
7.47
7.46
7.45
7.43
7.40
7.38
7.37
7.36
7.35
7.34
7.33
7.32
7.10
7.08
7.05
7.03
7.01
6.82
6.79
6.74
6.70
6.67
6.65
4.79
3.41 HDO
2.52 DMSO
5.00
2.02
2.01
1.91
f1 (ppm)

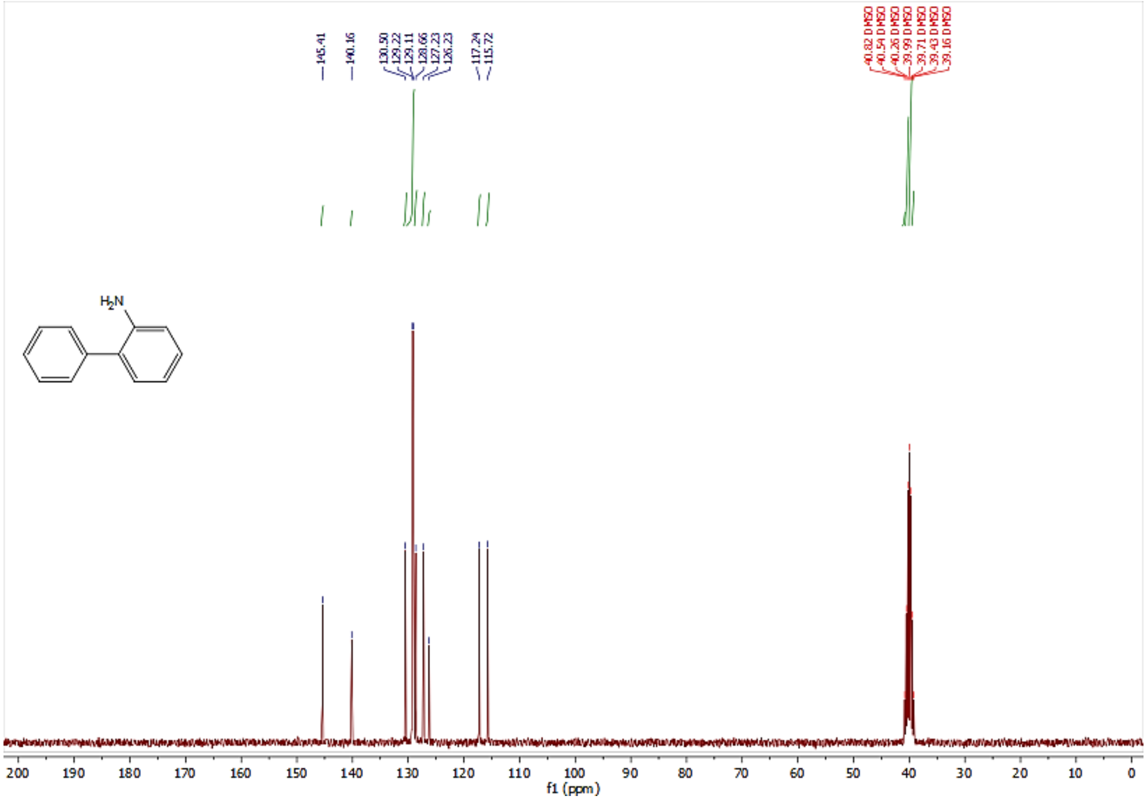
H2N
145.41
140.16
130.50
129.22
129.11
128.66
127.23
126.23
117.24
115.72
40.82 DMSO
40.54 DMSO
40.26 DMSO
39.99 DMSO
39.71 DMSO
39.43 DMSO
39.16 DMSO
f1 (ppm)

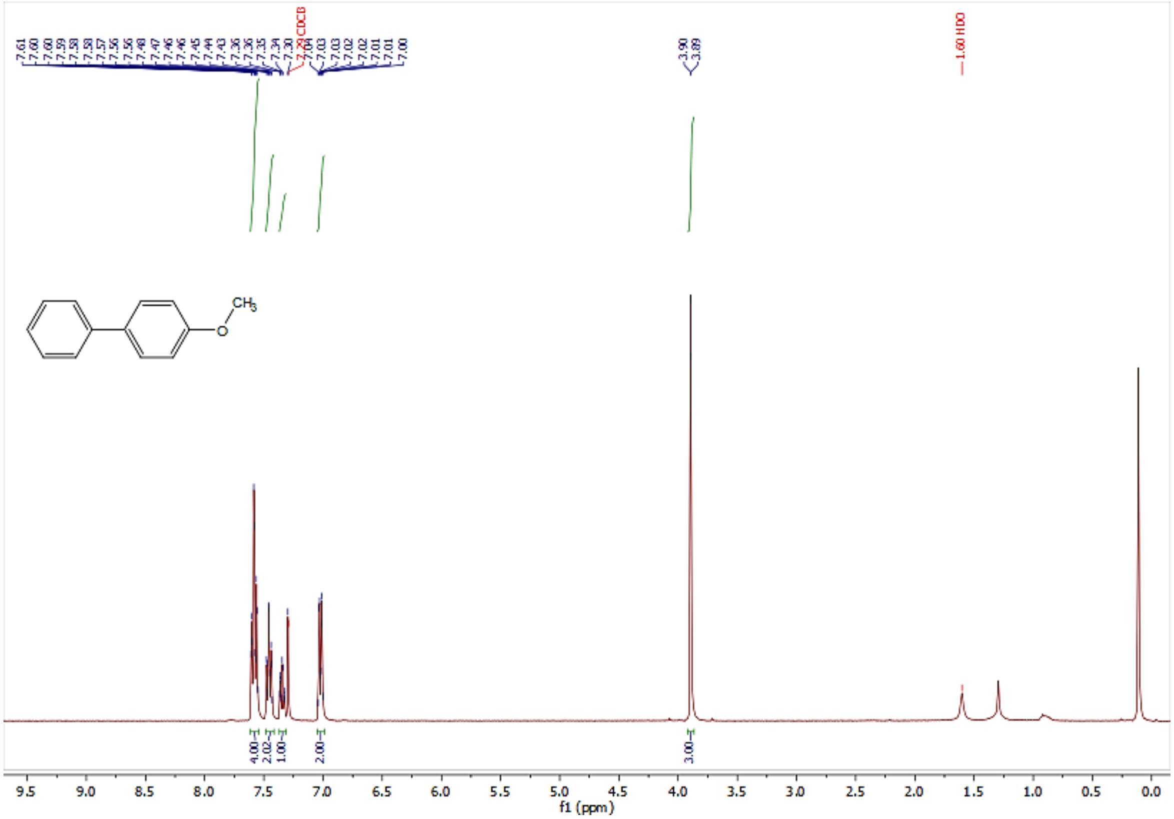
3.90
3.89
1.60 HDO
CH3
O
4.00
2.02
1.00
2.00
3.00
f1 (ppm)

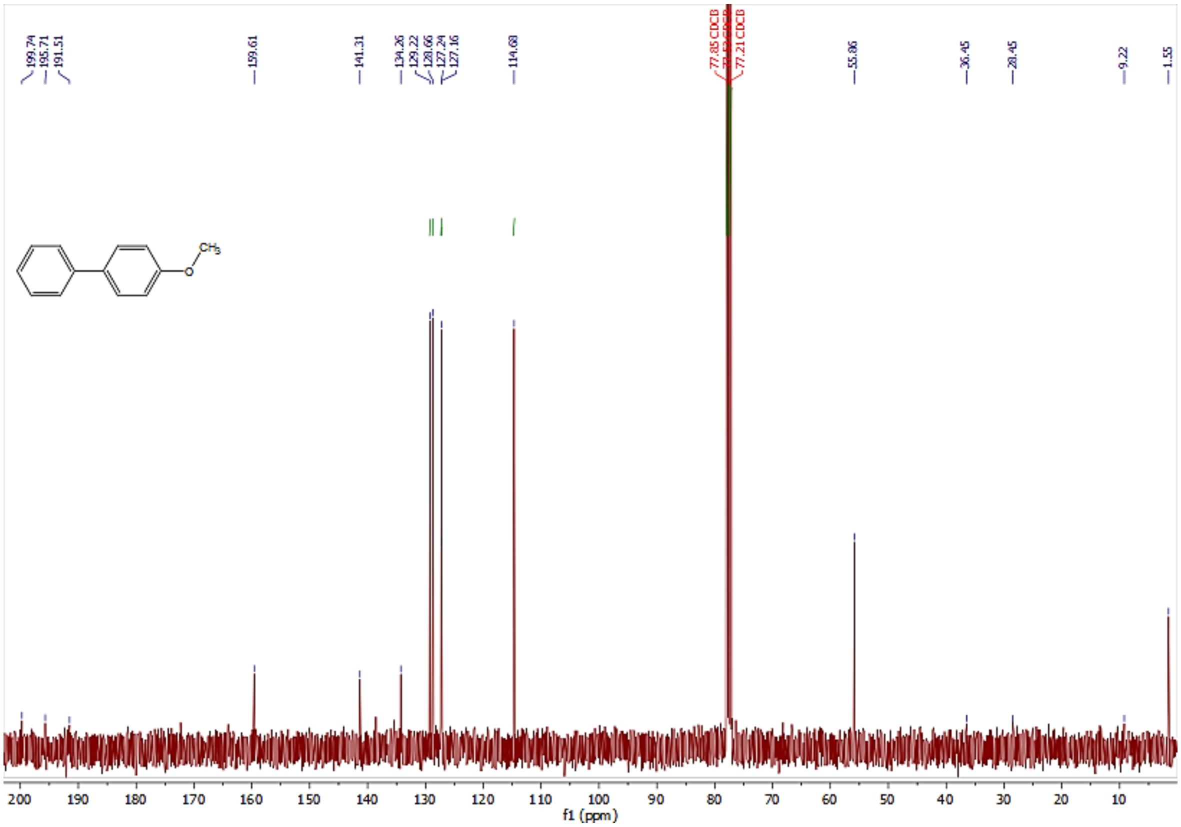
199.74
195.71
191.51
159.61
141.31
134.26
129.22
128.66
127.24
127.16
114.68
77.85 CDCB
77.21 CDCB
55.86
36.45
28.45
9.22
1.55
CH3
O
f1 (ppm)

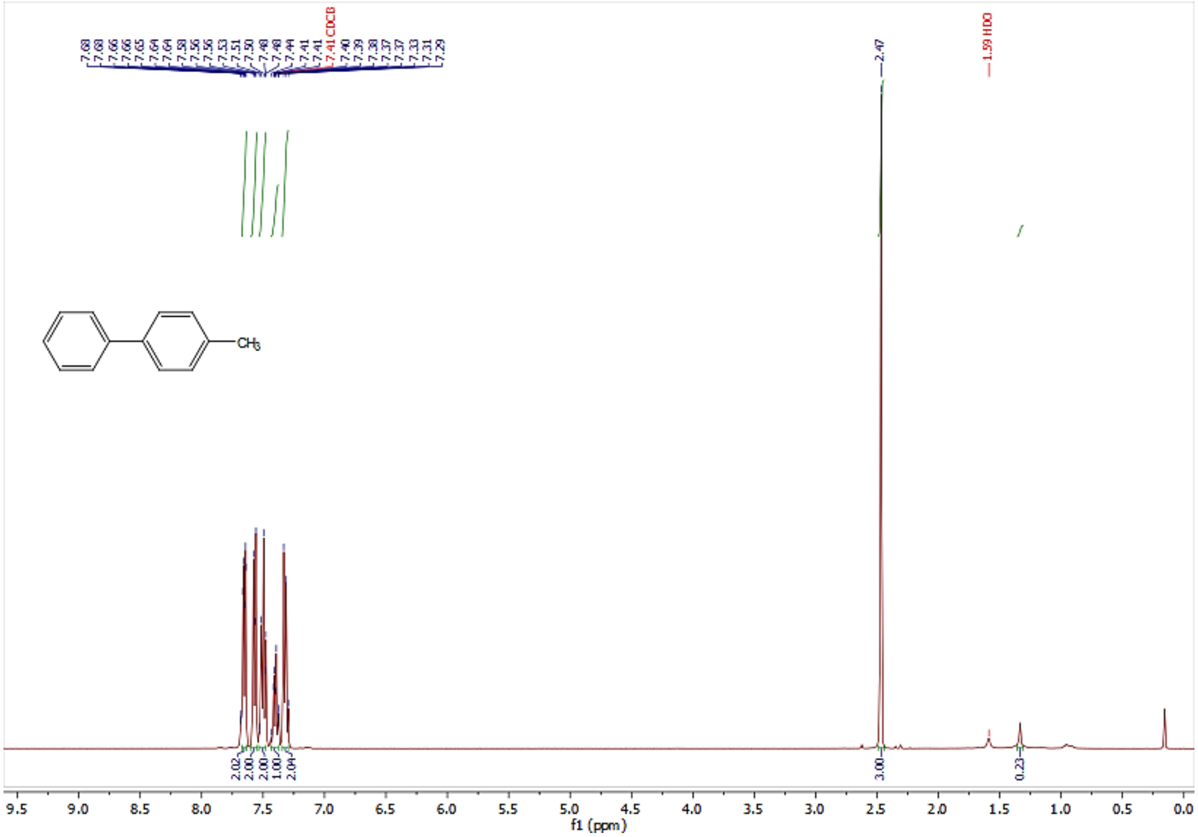

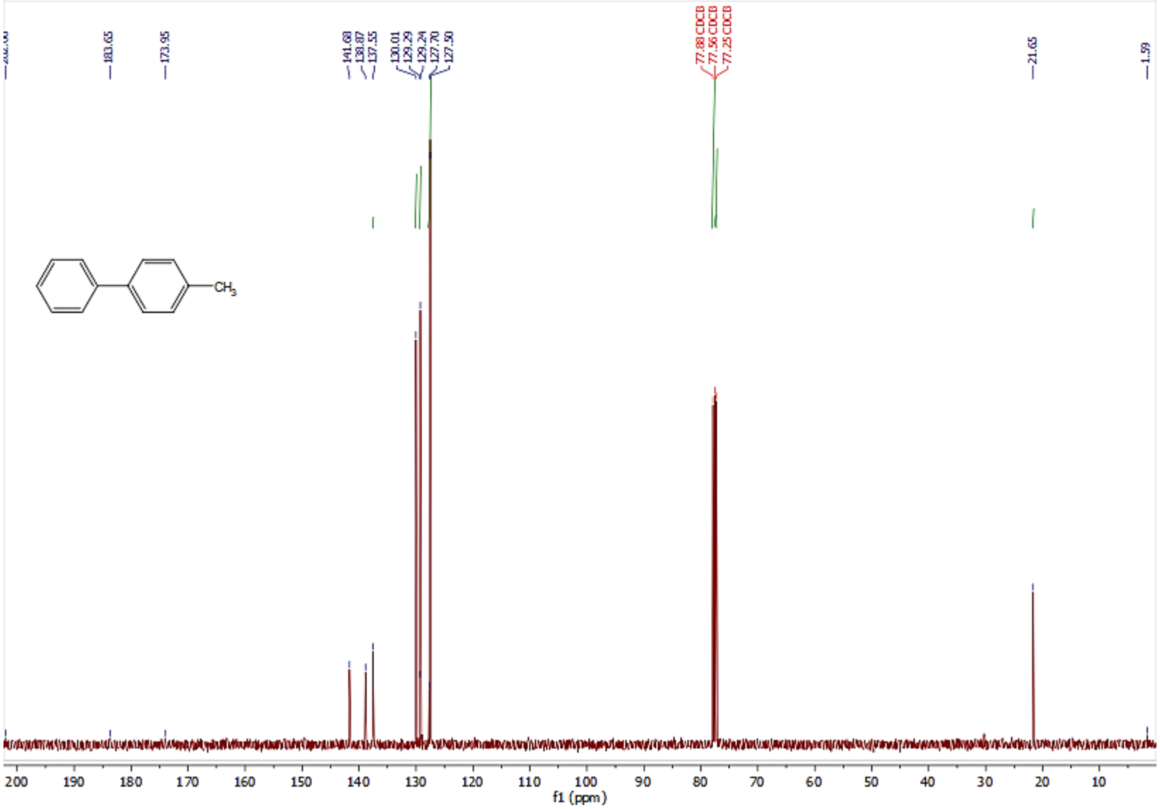

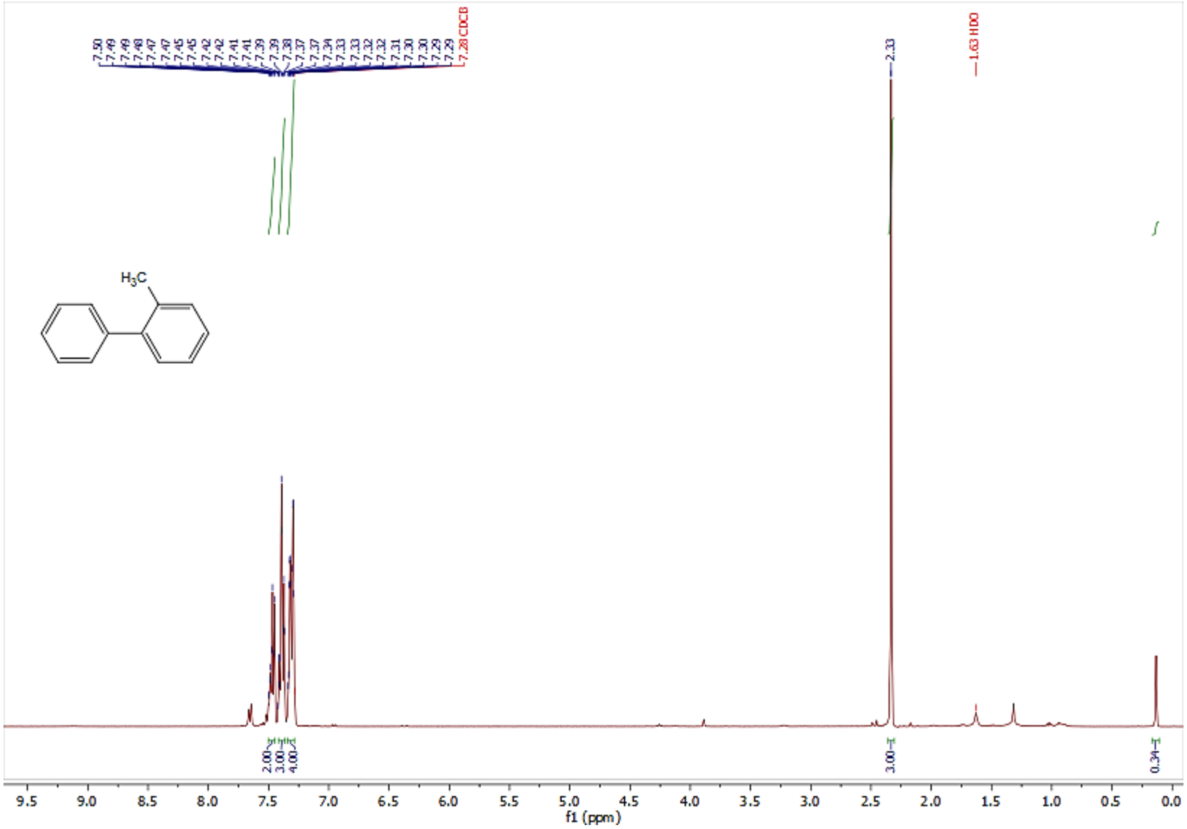
7.28 CDCl3
2.33
1.63 HDO
H3C
2.00
3.00
4.00
3.00
0.34
9.5 9.0 8.5 8.0 7.5 7.0 6.5 6.0 5.5 5.0 4.5 4.0 3.5 3.0 2.5 2.0 1.5 1.0 0.5 0.0
f1 (ppm)

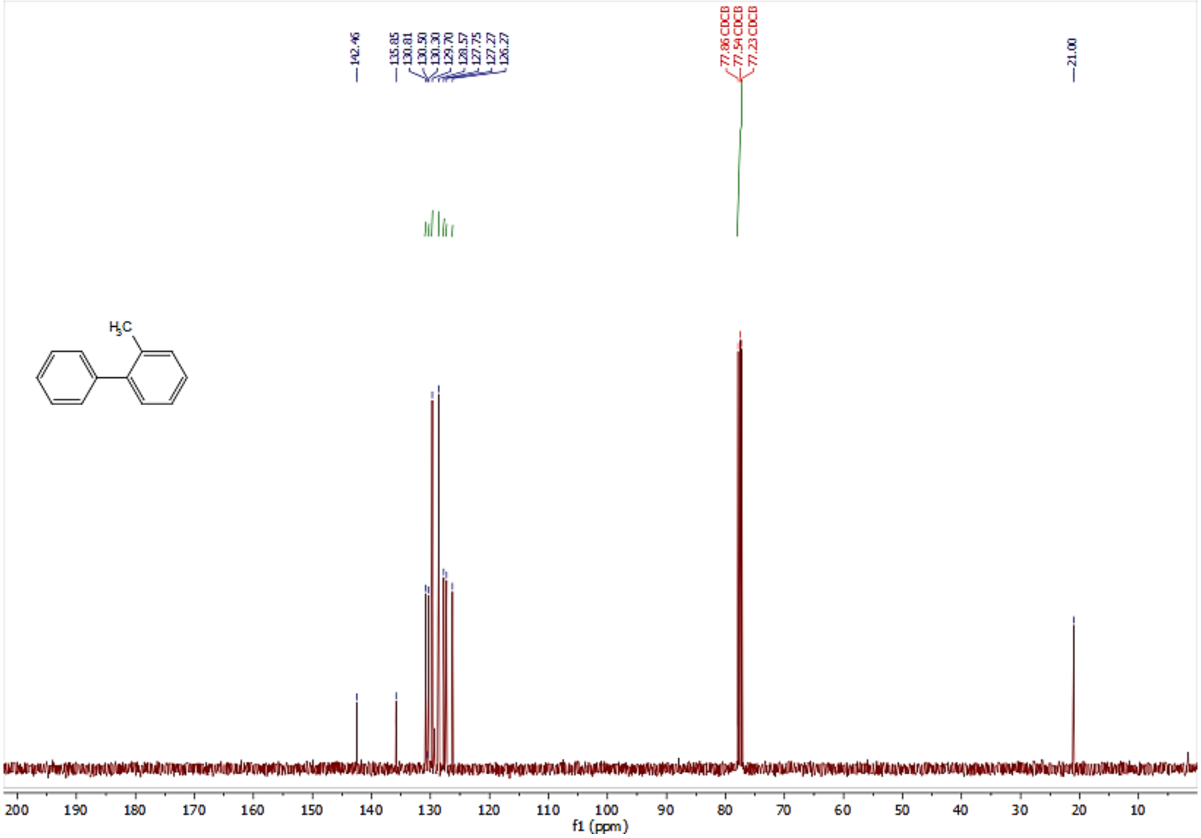
142.46
135.85
130.81
130.50
130.30
129.70
128.57
127.75
127.27
126.27
77.86 CDCl3
77.54 CDCl3
77.23 CDCl3
21.00
H3C
200 190 180 170 160 150 140 130 120 110 100 90 80 70 60 50 40 30 20 10
f1 (ppm)

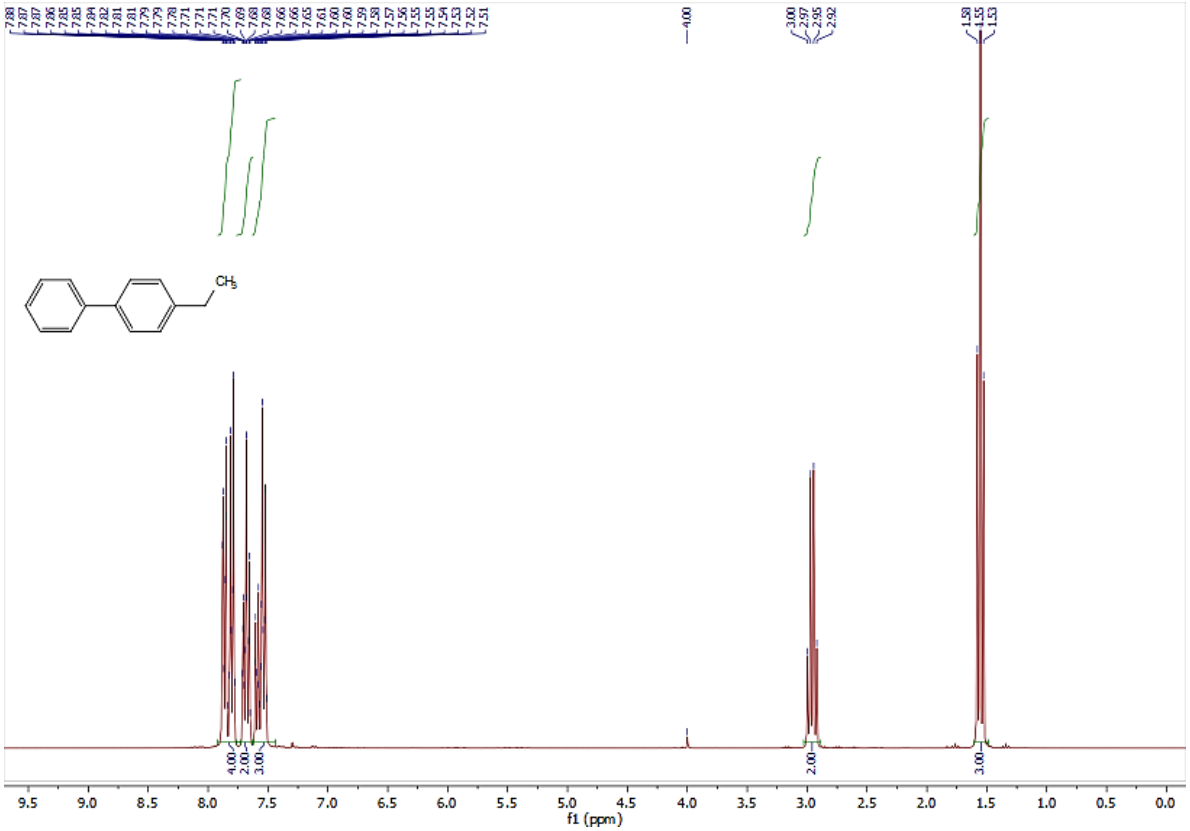
CH3
4.00
3.00
2.97
2.95
2.92
1.58
1.55
1.53
4.00
2.00
3.00
2.00
3.00
f1 (ppm)

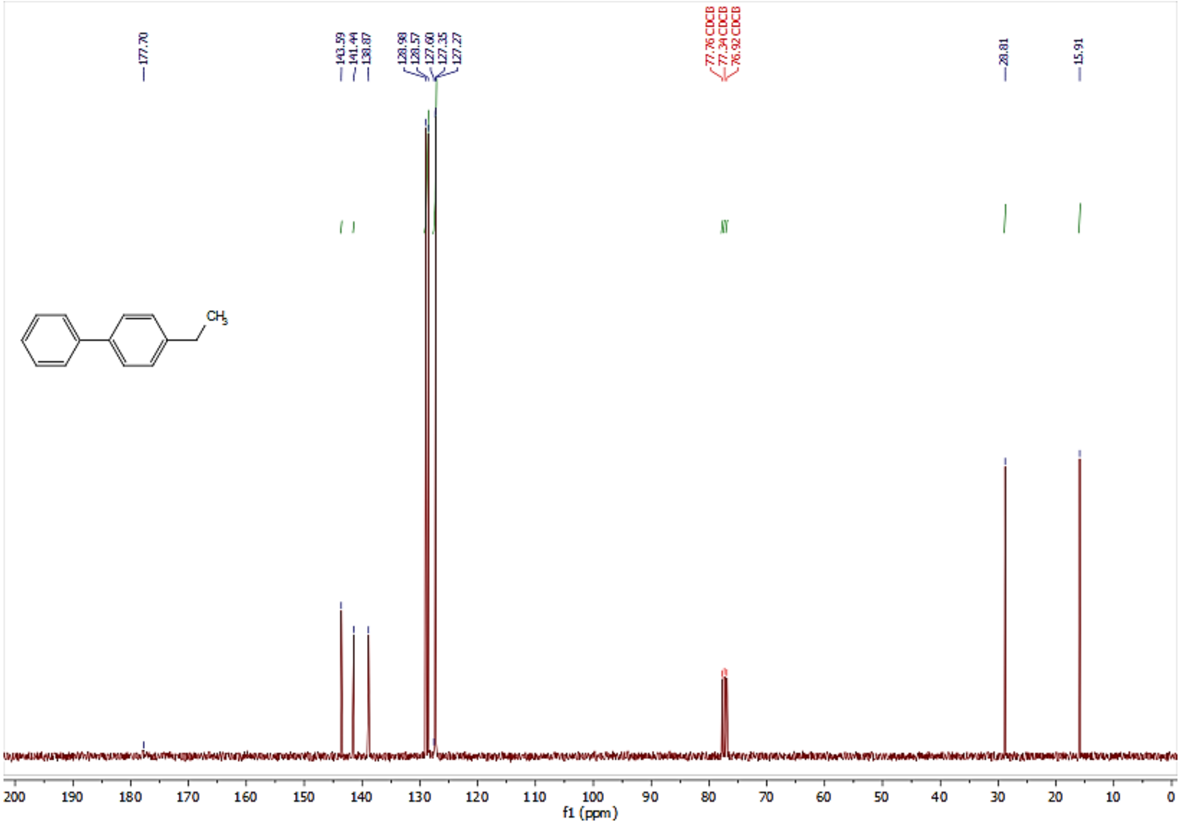
177.70
143.59
141.44
138.87
128.98
128.57
127.60
127.35
127.27
28.81
15.91
CH3
f1 (ppm)

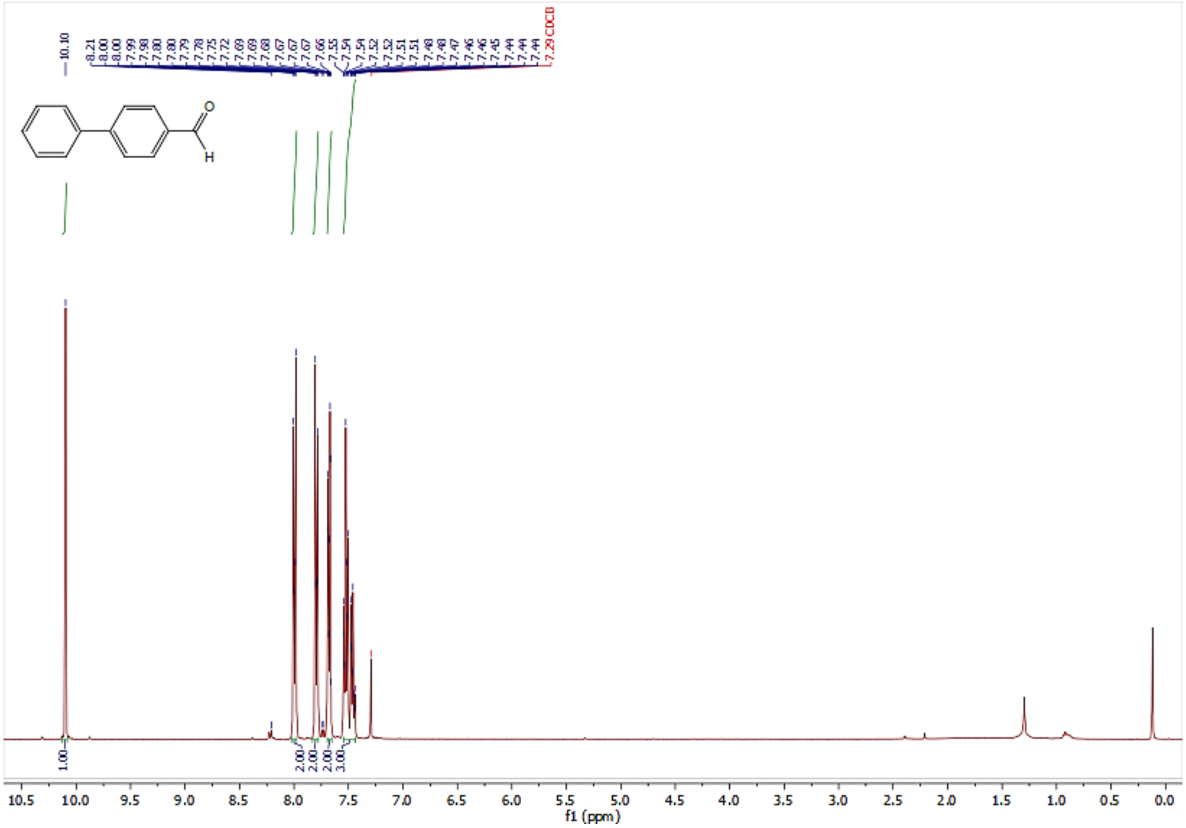
10.10
8.21 8.00 8.00 7.99 7.98 7.80 7.80 7.79 7.78 7.75 7.72 7.69 7.69 7.69 7.68 7.67 7.67 7.67 7.66 7.55 7.54 7.54 7.52 7.52 7.51 7.51 7.48 7.48 7.47 7.46 7.46 7.45 7.44 7.44 7.44
7.29 CDCl3
O
H
1.00
2.00
2.00
2.00
3.00
10.5 10.0 9.5 9.0 8.5 8.0 7.5 7.0 6.5 6.0 5.5 5.0 4.5 4.0 3.5 3.0 2.5 2.0 1.5 1.0 0.5 0.0
f1 (ppm)

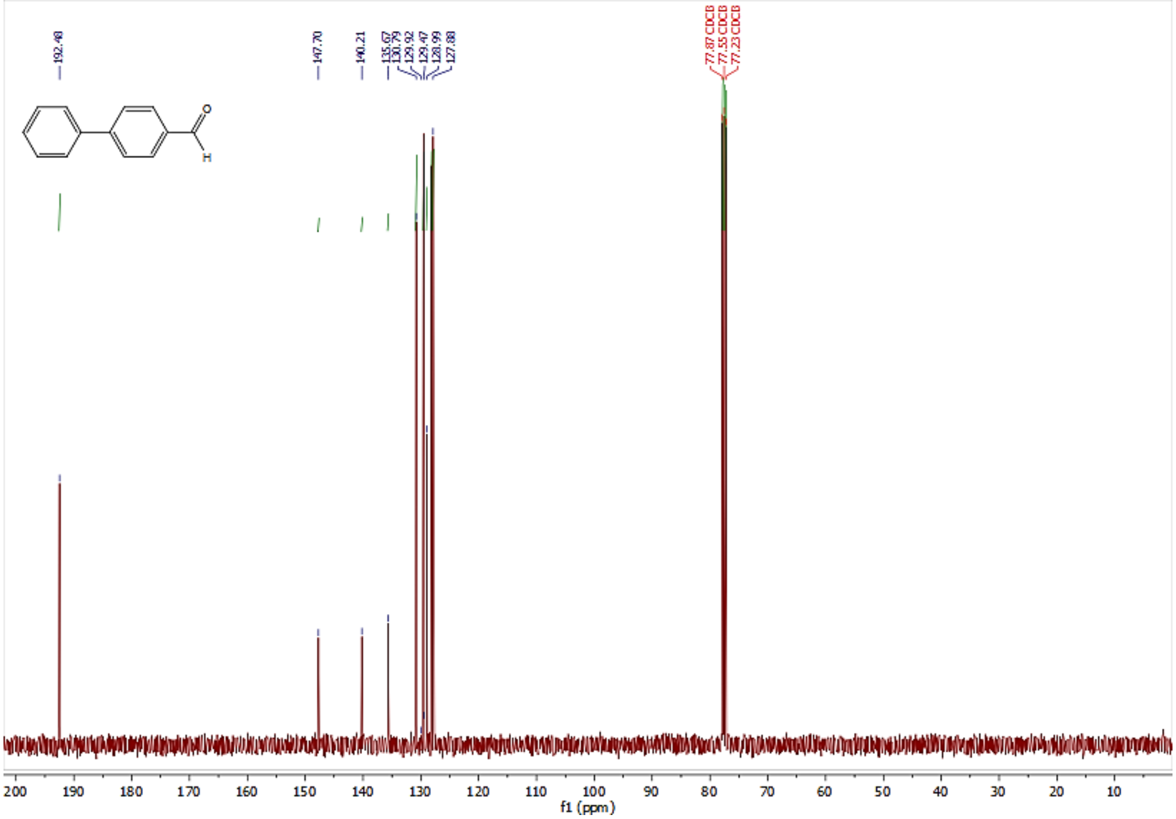
192.48
147.70
140.21
135.67 130.79 129.92 129.47 128.99 127.88
77.87 CDCl3
77.55 CDCl3
77.23 CDCl3
O
H
200 190 180 170 160 150 140 130 120 110 100 90 80 70 60 50 40 30 20 10
f1 (ppm)

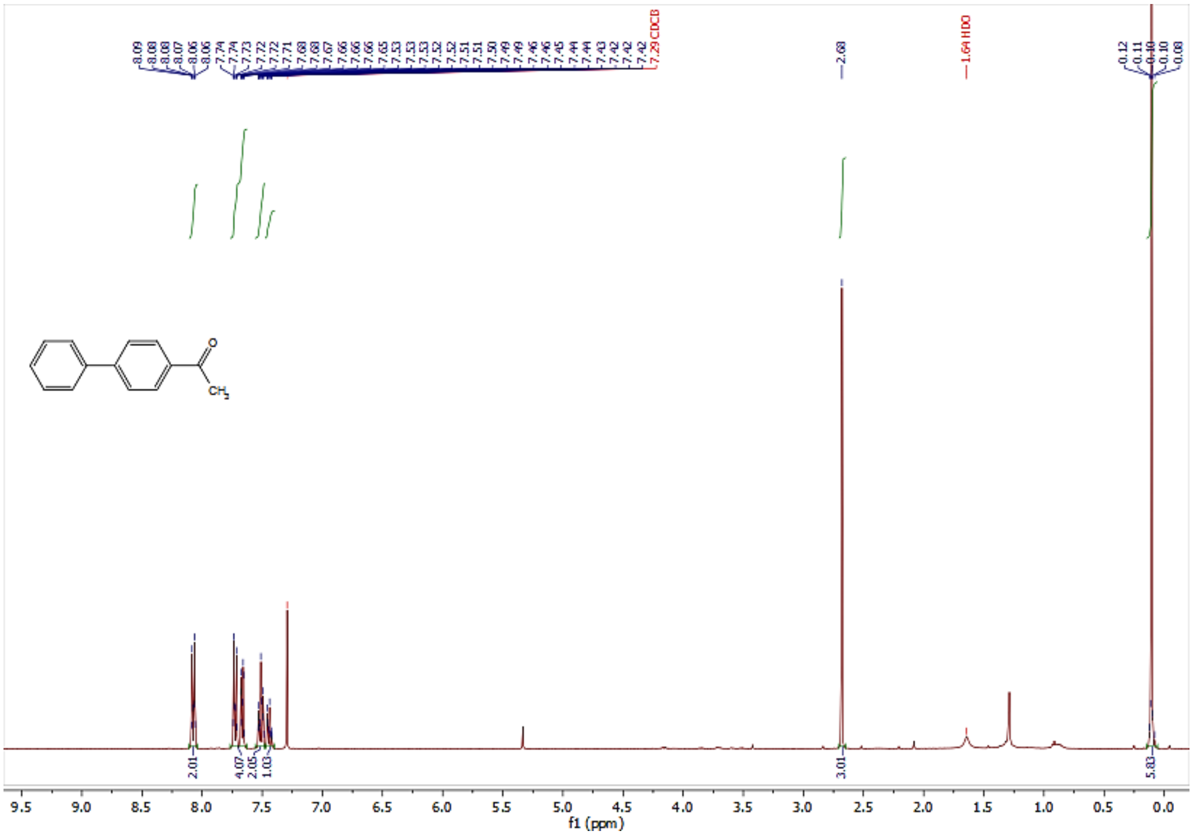
2.68
f1 (ppm)

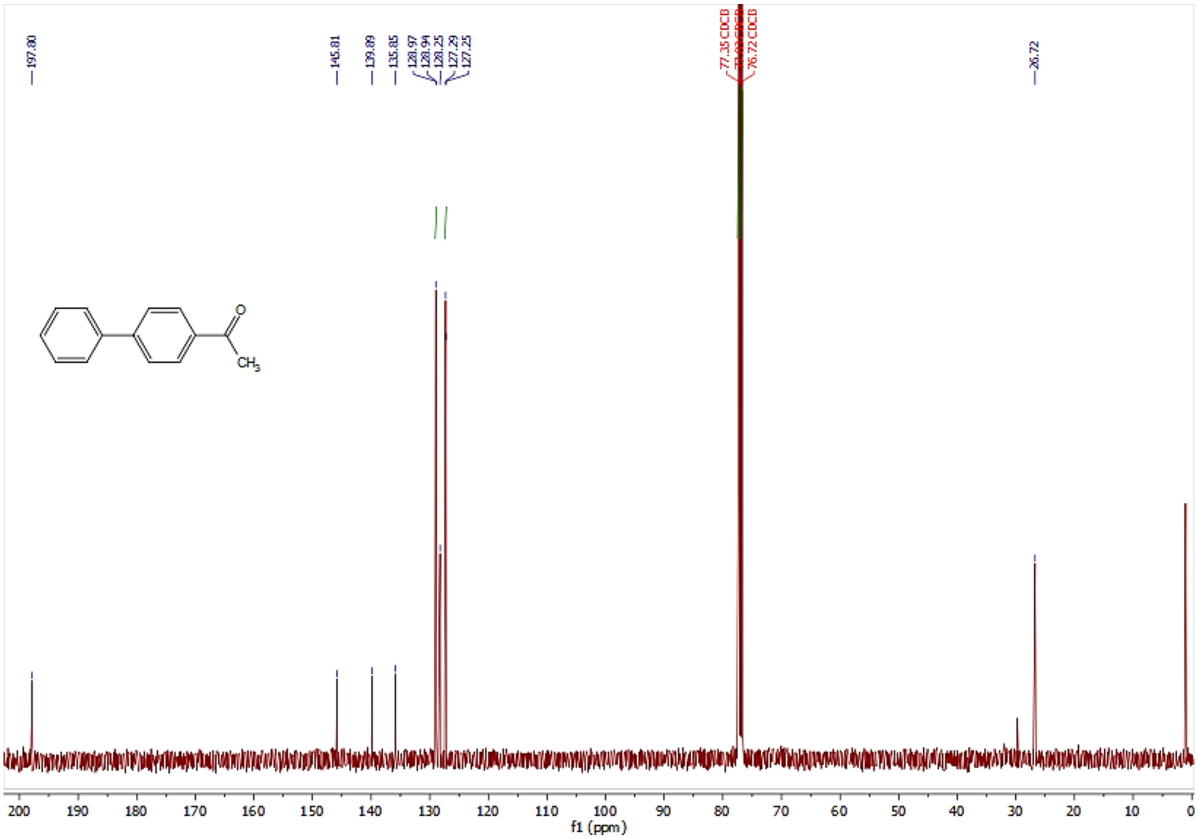
197.80
145.81
139.89
135.85
26.72
f1 (ppm)

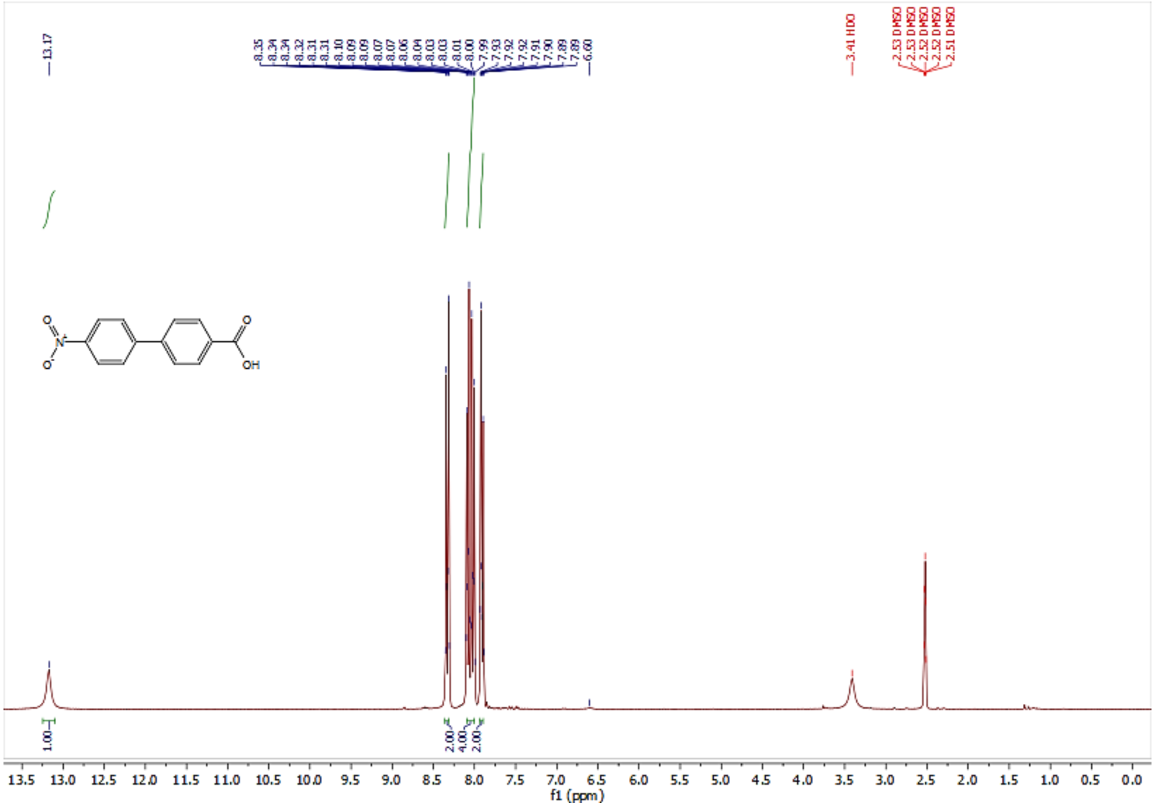
13.17
3.41 HDO
2.53 DMSO
2.51 DMSO
6.60
1.00
2.00
4.00
2.00
OH
f1 (ppm)

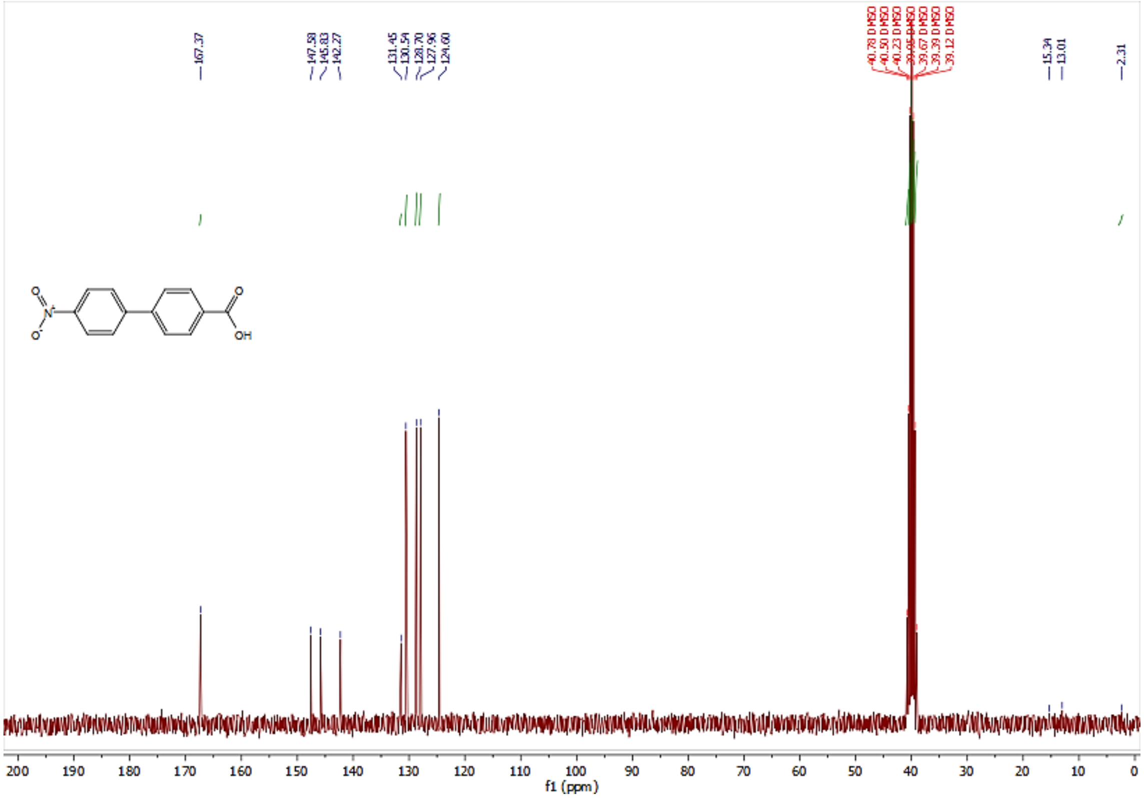
167.37
147.58
145.83
142.27
131.45
130.54
128.70
127.96
124.60
15.34
13.01
2.31
OH
f1 (ppm)

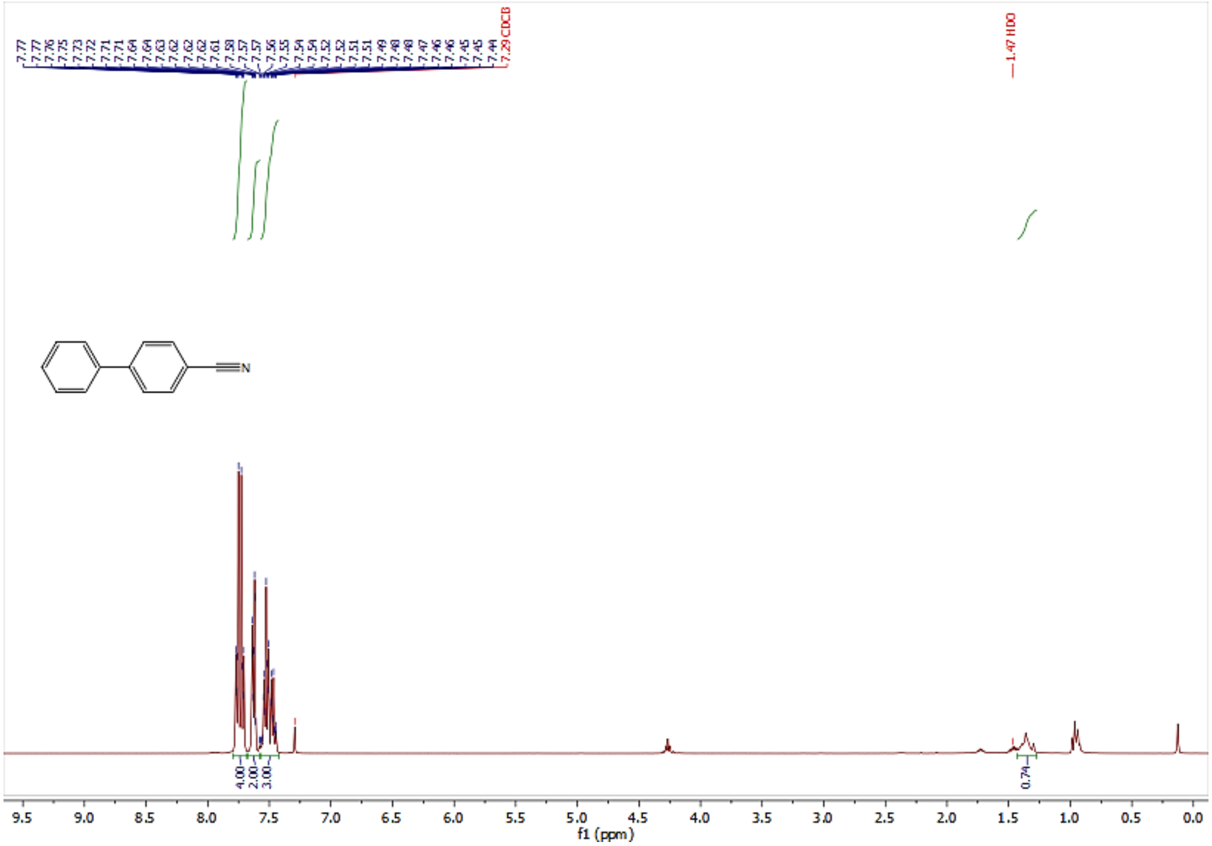
7.77
7.77
7.76
7.75
7.73
7.72
7.71
7.71
7.64
7.64
7.63
7.62
7.62
7.62
7.61
7.58
7.57
7.57
7.56
7.55
7.54
7.54
7.54
7.52
7.52
7.51
7.51
7.49
7.48
7.48
7.48
7.47
7.46
7.46
7.45
7.45
7.44
7.29 CDCl3
1.47 HDO
N
4.00
2.00
3.00
0.74
9.5 9.0 8.5 8.0 7.5 7.0 6.5 6.0 5.5 5.0 4.5 4.0 3.5 3.0 2.5 2.0 1.5 1.0 0.5 0.0
f1 (ppm)

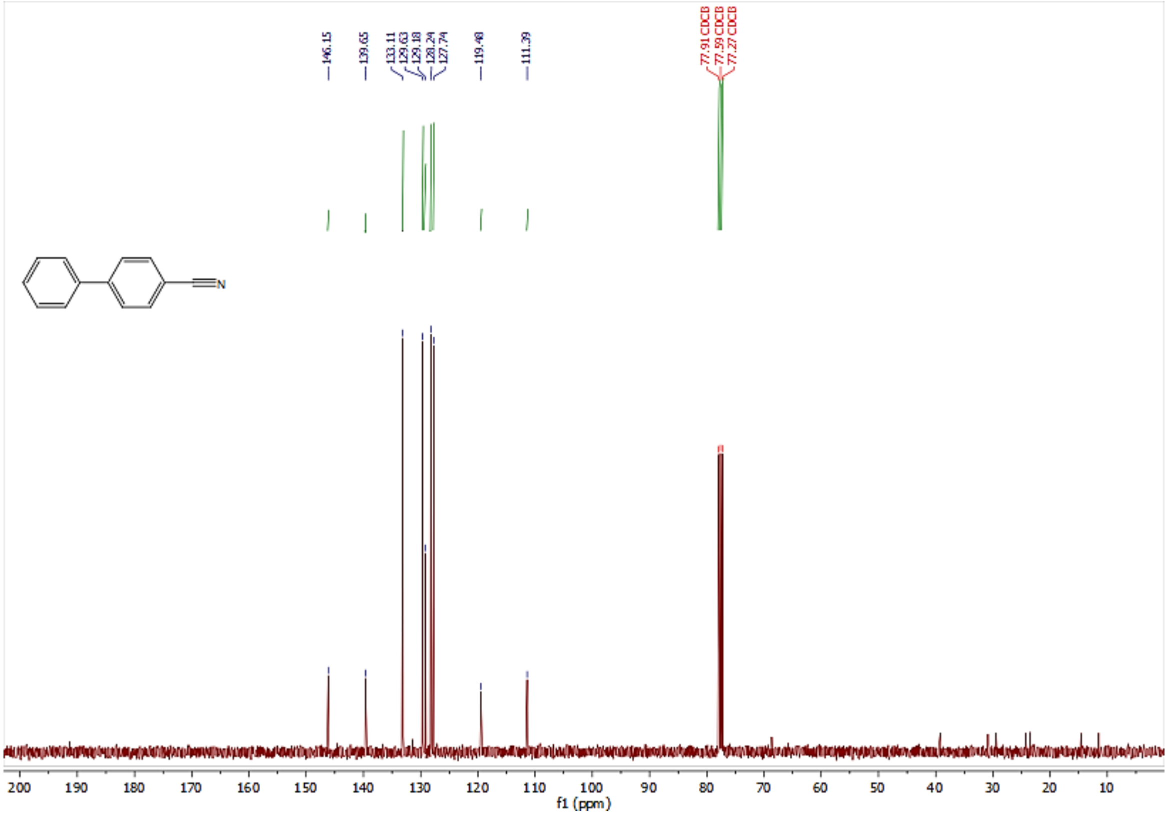
146.15
139.65
133.11
129.63
129.18
128.24
127.74
119.48
111.39
77.91 CDCl3
77.59 CDCl3
77.27 CDCl3
N
200 190 180 170 160 150 140 130 120 110 100 90 80 70 60 50 40 30 20 10
f1 (ppm)

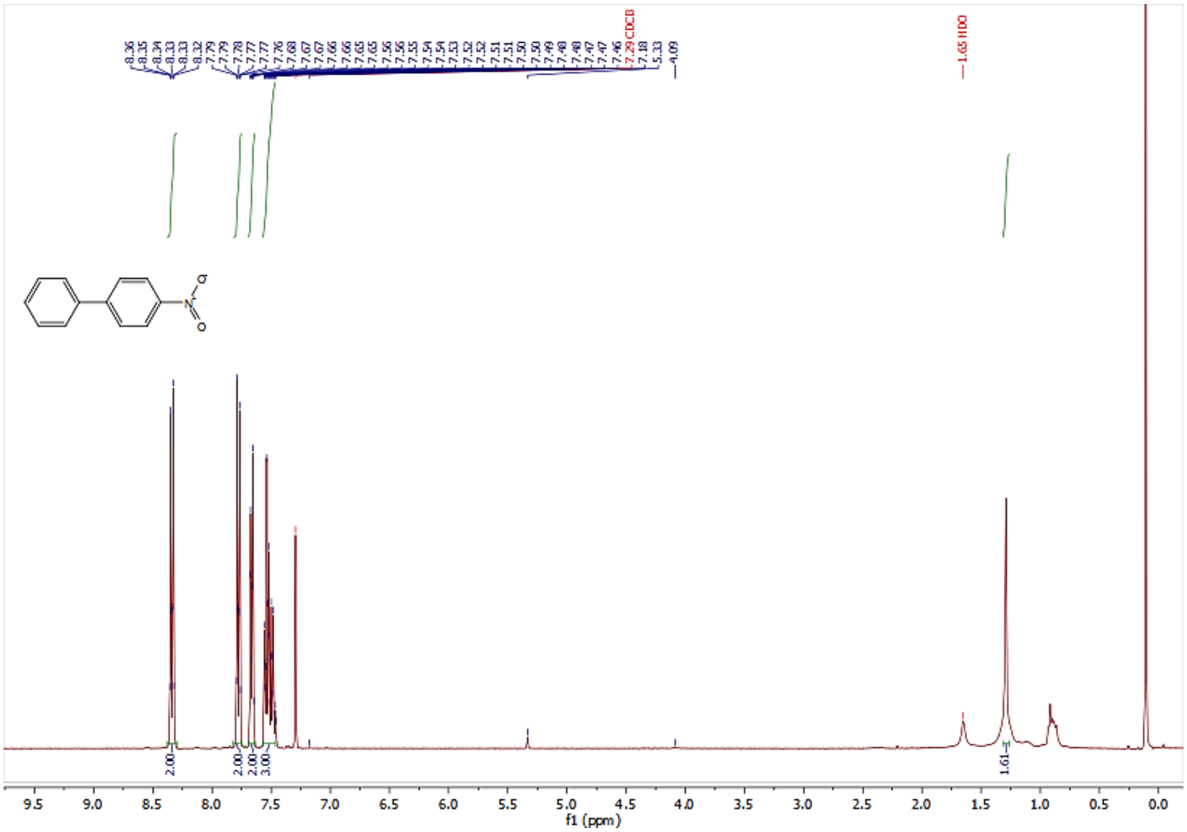
7.29 CDCl3
1.65 HDO
f1 (ppm)

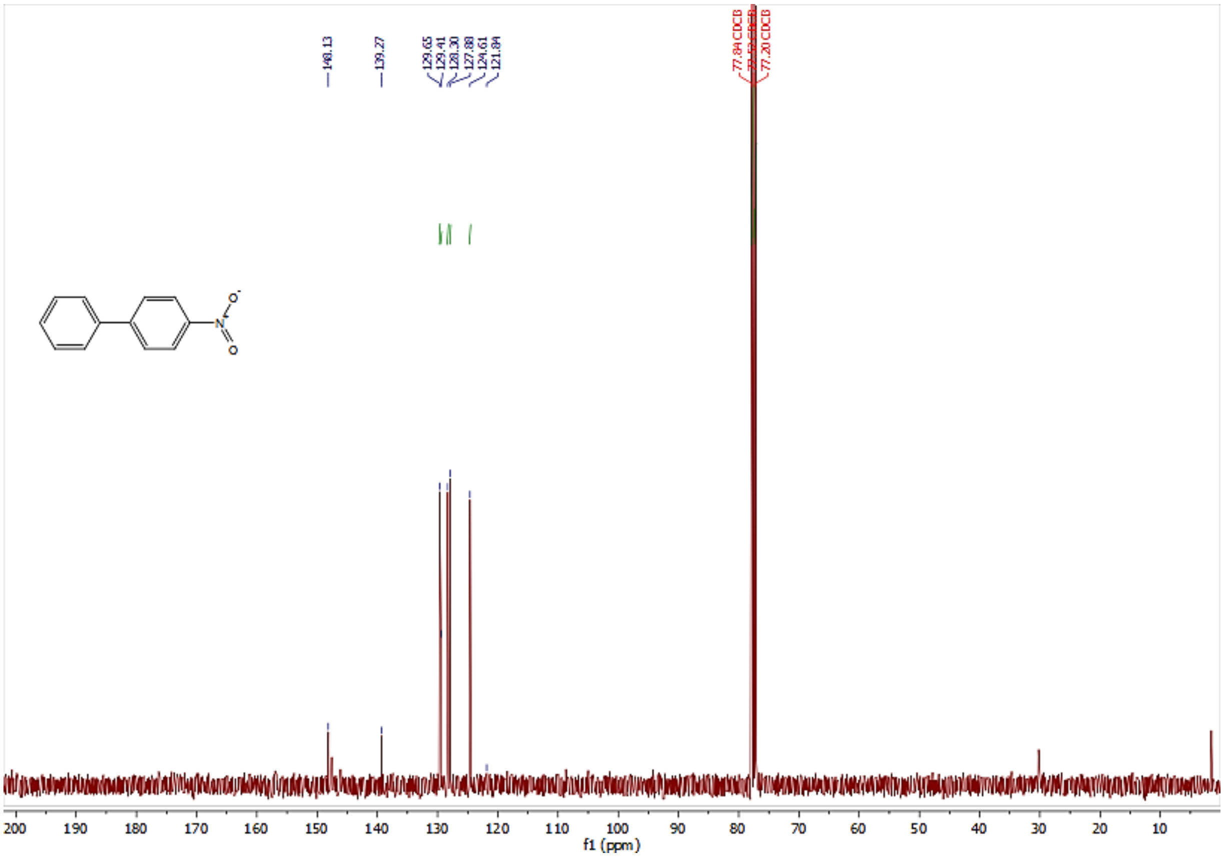
148.13
139.27
77.84 CDCl3
77.20 CDCl3
f1 (ppm)

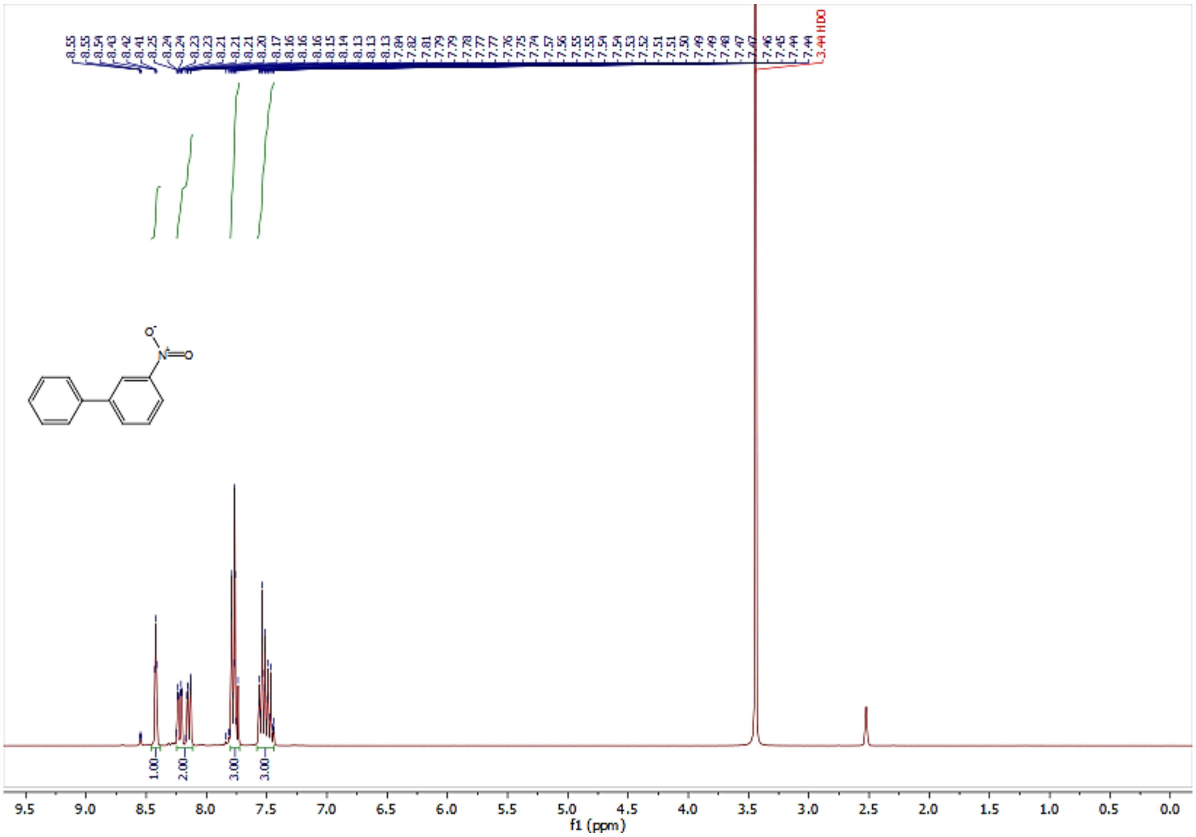

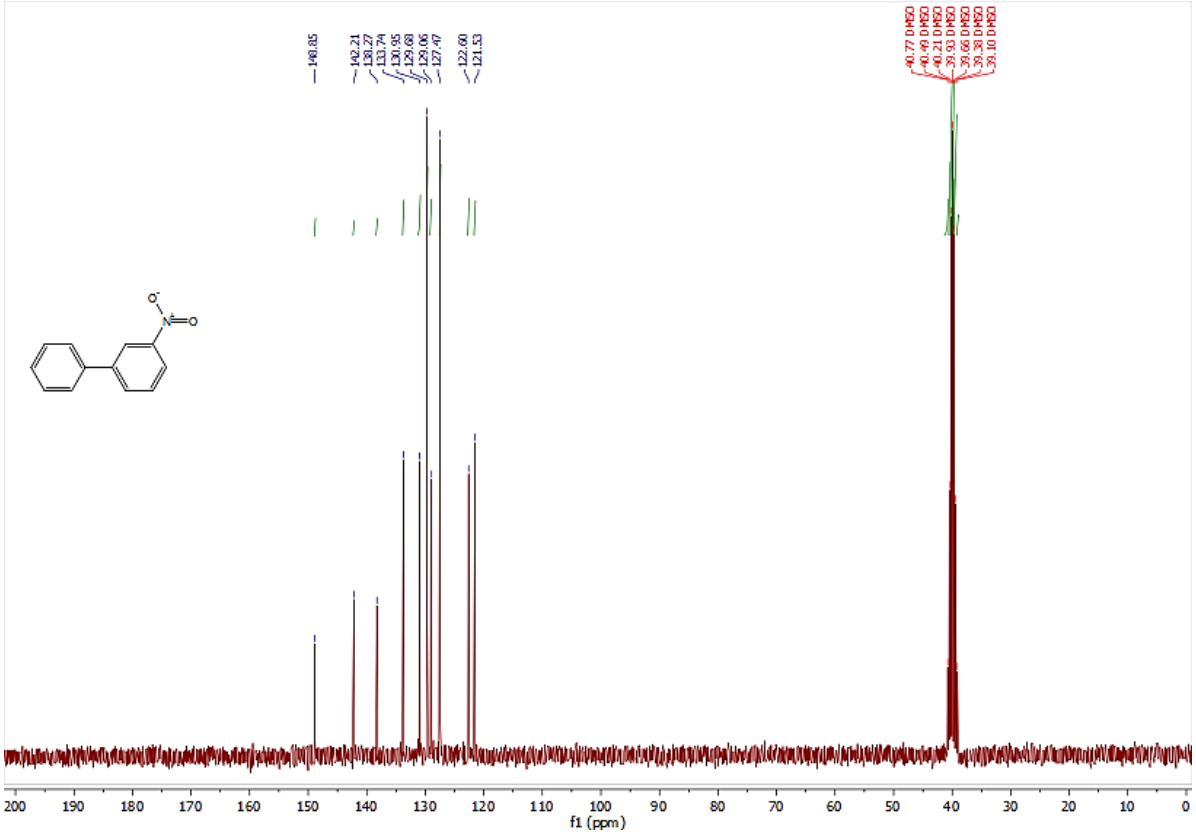

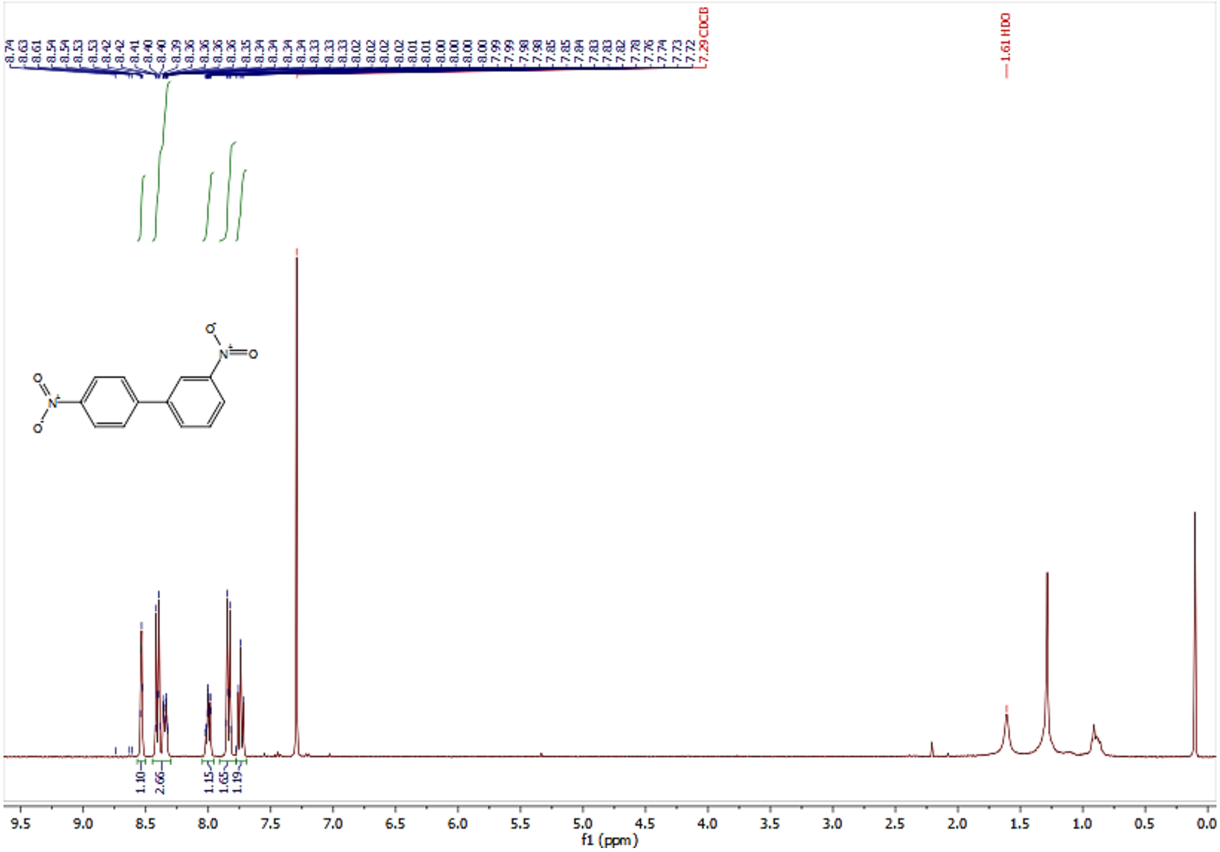

1.61 HDO
1.10
2.66
1.15
1.65
1.19
f1 (ppm)

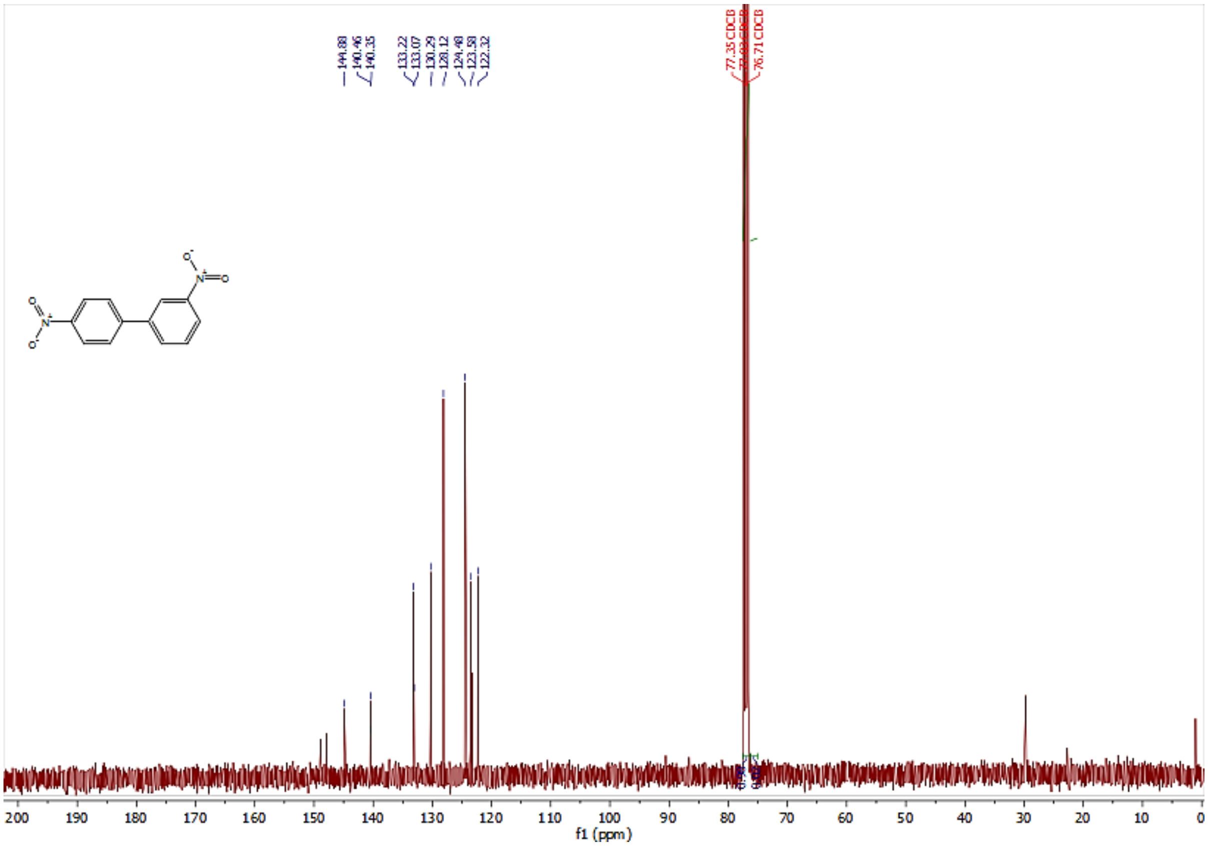

144.88
140.46
140.35
133.22
133.07
130.29
128.12
124.48
123.58
122.32
76.71 CDCl3
f1 (ppm)

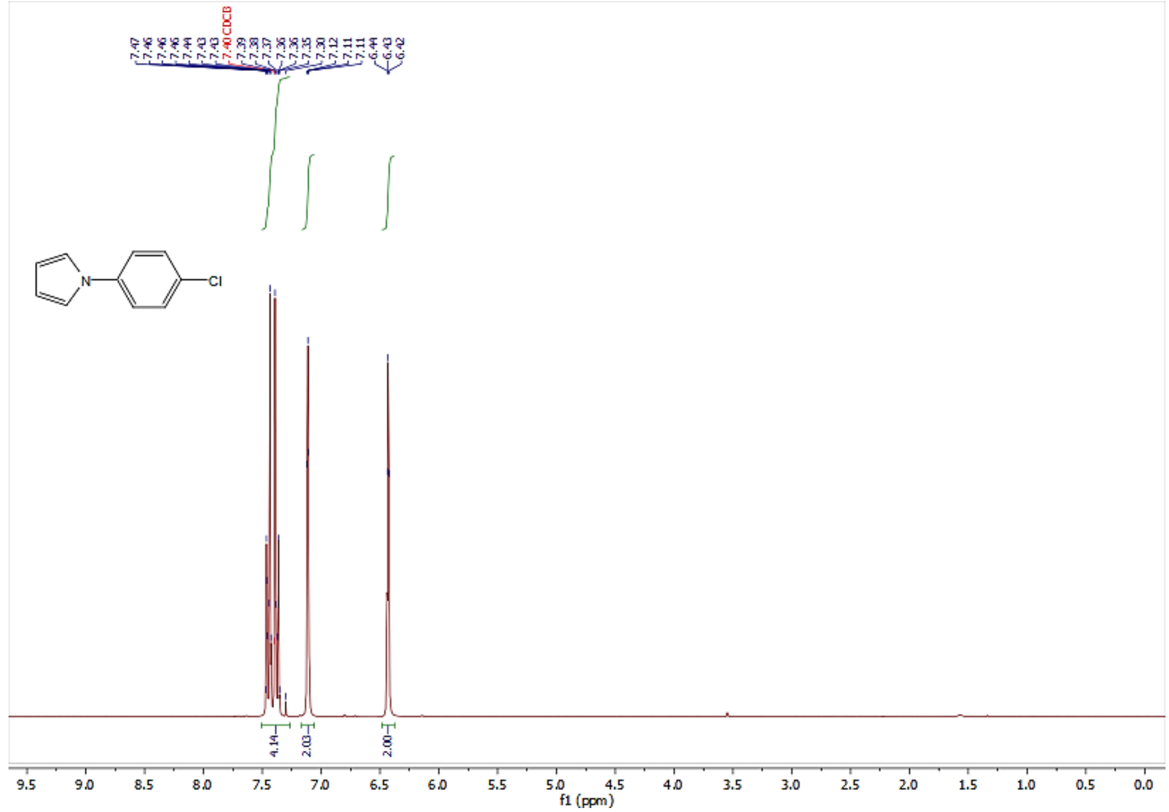
7.47
7.46
7.46
7.46
7.44
7.43
7.43
7.40 CDCB
7.39
7.38
7.37
7.36
7.36
7.35
7.30
7.12
7.11
7.11
6.44
6.43
6.42
N
Cl
4.14
2.03
2.00
9.5
9.0
8.5
8.0
7.5
7.0
6.5
6.0
5.5
5.0
4.5
4.0
3.5
3.0
2.5
2.0
1.5
1.0
0.5
0.0
f1 (ppm)

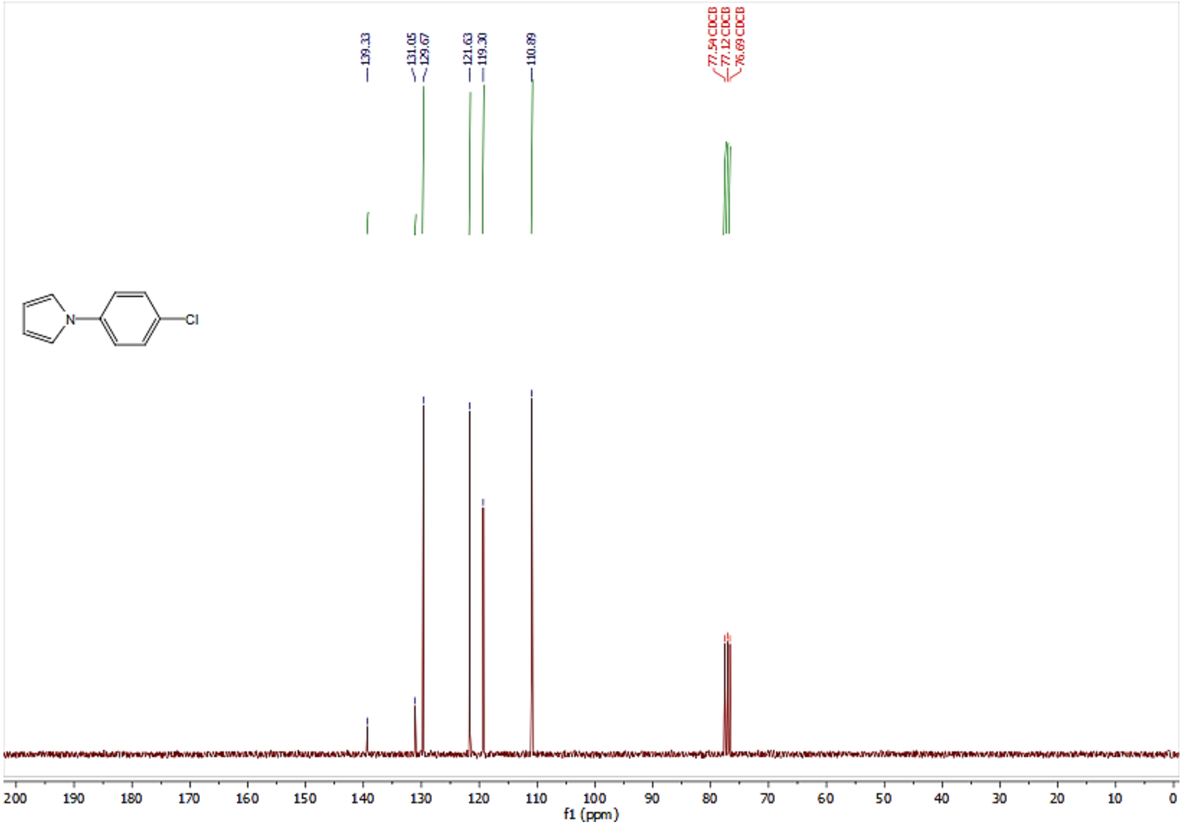
139.33
131.05
129.67
121.63
119.30
110.89
77.54 CDCB
77.12 CDCB
76.69 CDCB
N
Cl
200
190
180
170
160
150
140
130
120
110
100
90
80
70
60
50
40
30
20
10
0
f1 (ppm)

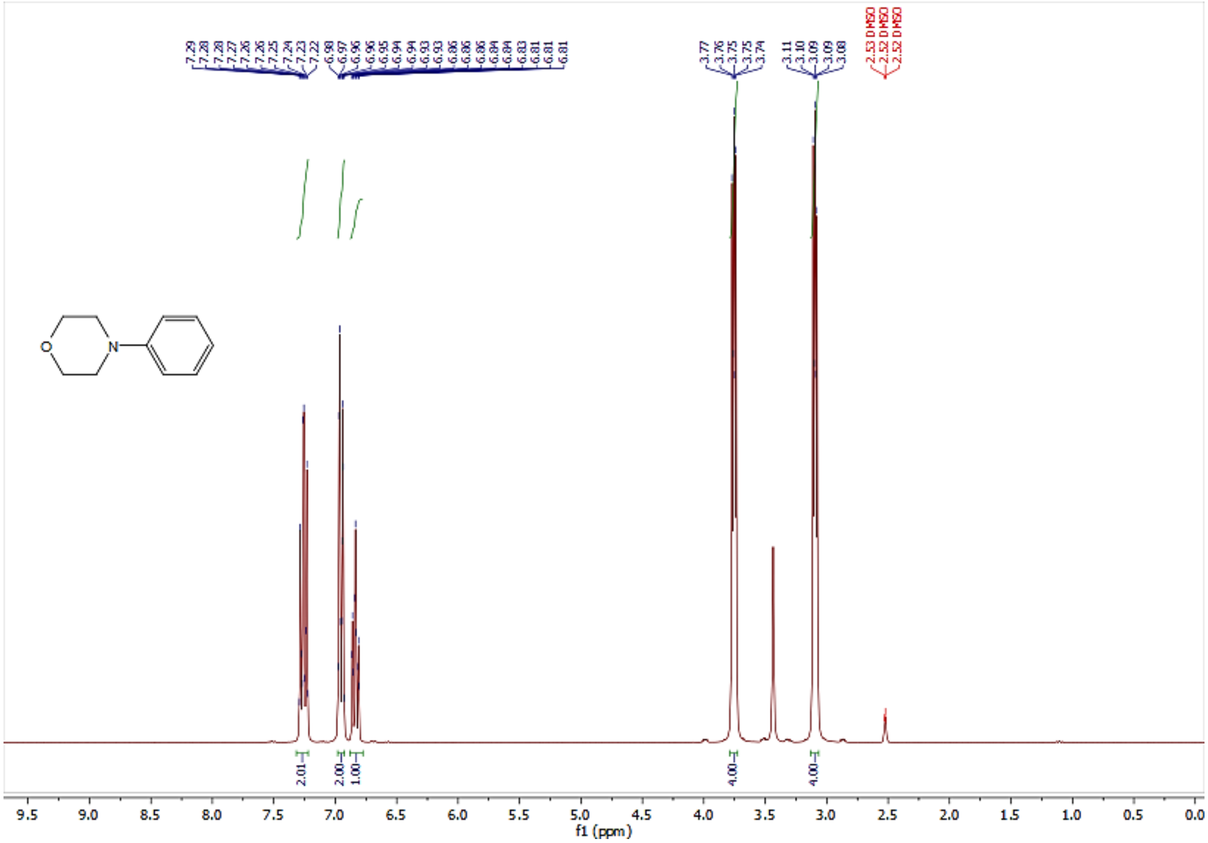
7.29
7.28
7.28
7.27
7.26
7.26
7.25
7.24
7.23
7.22
6.98
6.97
6.96
6.96
6.95
6.94
6.94
6.94
6.93
6.93
6.86
6.86
6.86
6.84
6.84
6.83
6.81
6.81
6.81
3.77
3.76
3.75
3.75
3.74
3.11
3.10
3.09
3.09
3.08
2.53 DMSO
2.52 DMSO
2.52 DMSO
O
N
2.01
2.00
1.00
4.00
4.00
f1 (ppm)

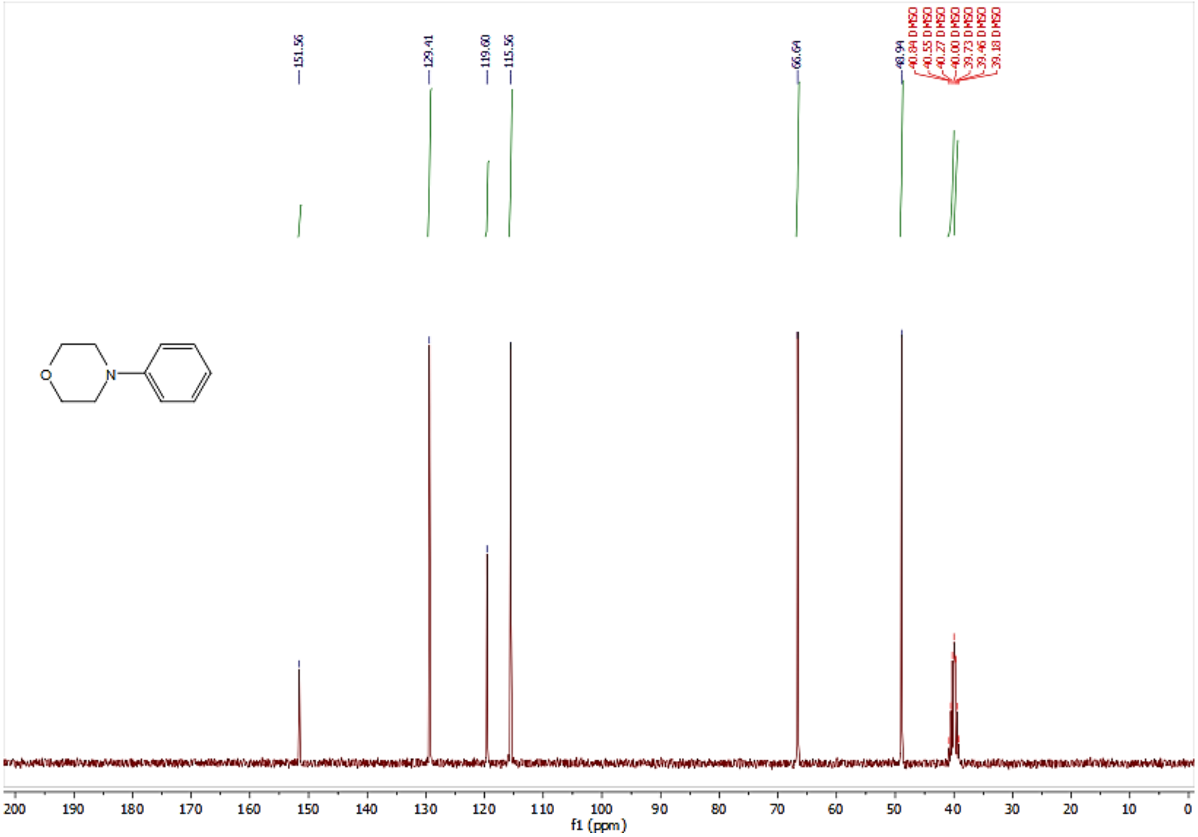
151.56
129.41
119.60
115.56
66.64
48.94
40.84 DMSO
40.55 DMSO
40.27 DMSO
40.00 DMSO
39.73 DMSO
39.46 DMSO
39.18 DMSO
O
N
f1 (ppm)

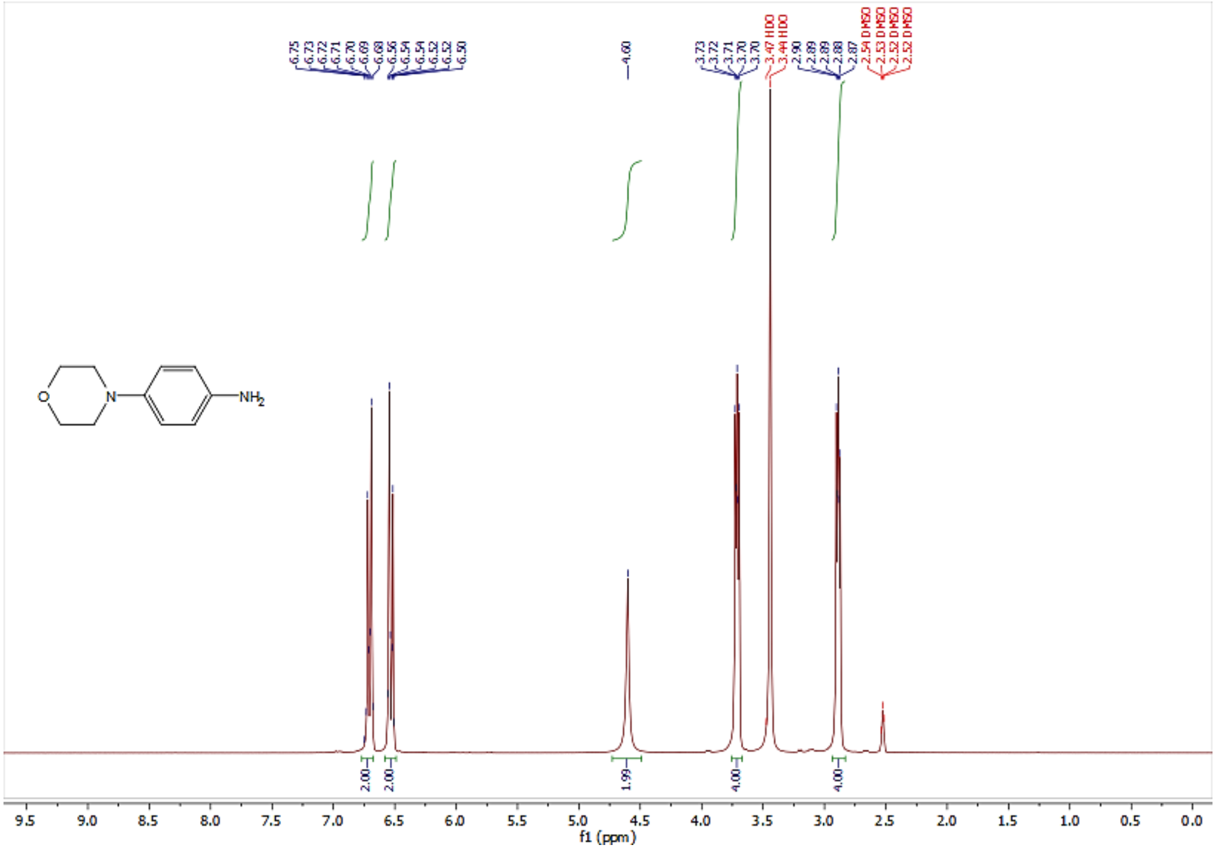
6.75
6.73
6.72
6.71
6.70
6.69
6.68
6.56
6.55
6.54
6.52
6.52
6.50
4.60
3.73
3.72
3.71
3.70
3.70
3.47 HDO
3.44 HDO
2.90
2.89
2.89
2.88
2.87
2.54 DMSO
2.53 DMSO
2.52 DMSO
2.52 DMSO
NH2
2.00
2.00
1.99
4.00
4.00
f1 (ppm)

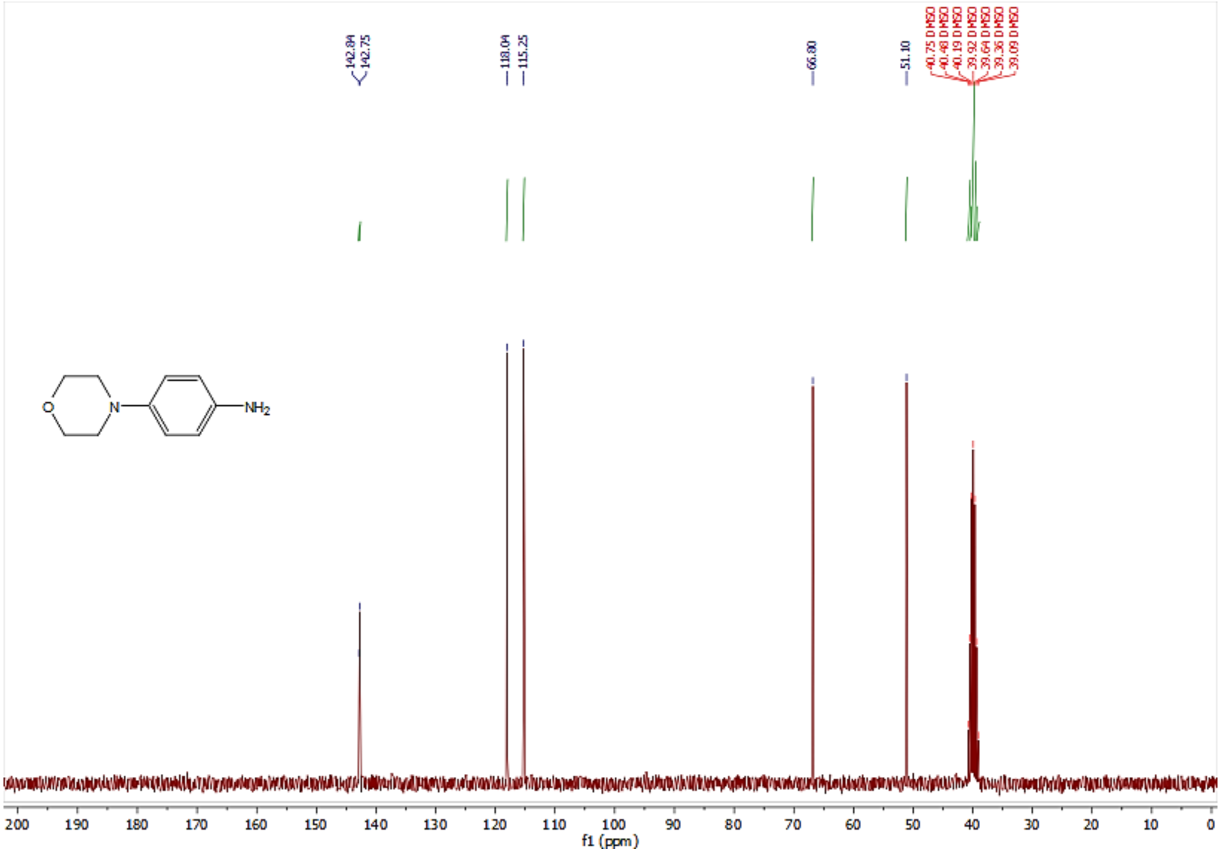
142.84
142.75
118.04
115.25
66.80
51.10
40.75 DMSO
40.48 DMSO
40.19 DMSO
39.92 DMSO
39.64 DMSO
39.36 DMSO
39.09 DMSO
NH2
f1 (ppm)

References:

1. Sobhani, S.; Pakdin-Parizi, Z. Palladium-DABCO complex supported on γ-Fe_2O_3 magnetic nanoparticles: A new catalyst for C-C bond formation via Mizoroki-Heck cross-coupling reaction. *Appl. Catal. A: Gen.* **2014,** *479*, 112-120.

2. Keihan, A. H.; Veisi, H.; Biabri, P. M. Facile synthesis of PEG-coated magnetite (Fe_3O_4) and embedment of gold nanoparticle as a nontoxic antimicrobial agent. *Appl. Organomet. Chem.* **2017,** *31*, e3873.

3. Tyagi, A.; Yamamoto, A.; Yoshida, H., Photocatalytic Ullmann coupling of aryl halides by a novel blended catalyst consisting of a TiO_2 photocatalyst and an Al_2O_3 supported Pd–Au bimetallic catalyst. *Catal. Sci. Technol.* **2018,** *8*, 6196-6203.

4. Sahoo, M.; Mansingh, S.; Subudhi, S.; Mohapatra, P.; Parida, K., A plasmonic AuPd bimetallic nanoalloy decorated over a GO/LDH hybrid nanocomposite via a green synthesis route for robust Suzuki coupling reactions: a paradigm shift towards a sustainable future. *Catal. Sci. Technol.* **2019,** *9*, 4678-4692.

5. Chen, T.; Gao, J.; Shi, M., A novel tridentate NHC–Pd (II) complex and its application in the Suzuki and Heck-type cross-coupling reactions. *Tetrahedron.* **2006,** *62*, 6289-6294.

6. Fortea-Pérez, F. R.; Schlegel, I.; Julve, M.; Armentano, D.; De Munno, G.; Stiriba, S.-E., Sustainable carbon–carbon bond formation catalyzed by new oxamate-containing palladium (II) complexes in ionic liquids. *J. Organomet. Chem.* **2013,** *743*, 102-108.

7. Jang, Y.; Chung, J.; Kim, S.; Jun, S. W.; Kim, B. H.; Lee, D. W.; Kim, B. M.; Hyeon, T., Simple synthesis of Pd–Fe_3O_4 heterodimer nanocrystals and their application as a magnetically recyclable catalyst for Suzuki cross-coupling reactions. *Phys. Chem. Chem. Phys.* **2011,** *13*, 2512-2516.

8. Shylesh, S.; Wang, L.; Demeshko, S.; Thiel, W. R., Facile synthesis of mesoporous magnetic nanocomposites and their catalytic application in carbon–carbon coupling reactions. *ChemCatChem* **2010,** *2*, 1543-1547.

9. Liao, Y.; He, L.; Huang, J.; Zhang, J.; Zhuang, L.; Shen, H.; Su, C.-Y., Magnetite nanoparticle-supported coordination polymer nanofibers: synthesis and catalytic application in Suzuki-Miyaura coupling. *ACS Appl. Mater. Interfaces.* **2010,** *2*, 2333-2338.

10. Moghadam, H. H.; Sobhani, S.; Sansano, J. M., New nanomagnetic heterogeneous cobalt catalyst for the synthesis of aryl nitriles and biaryls. *ACS omega* **2020,** *5*, 18619-18627.

11. Keesara, S.; Parvathaneni, S.; Dussa, G.; Mandapati, M. R., Polystyrene supported thiopseudourea Pd (II) complex: applications for Sonogashira, Suzuki-Miyaura, Heck, Hiyama and Larock heteroannulation reactions. *J. Organomet. Chem.* **2014,** *765*, 31-38.

12. Nuri, A.; Mansoori, Y.; Bezaatpour, A.; Shchukarev, A.; Mikkola, J. P., Magnetic Mesoporous SBA-15 Functionalized with a NHC Pd (II) Complex: An Efficient and Recoverable Nanocatalyst for Hiyama Reaction. *ChemistrySelect.* **2019,** *4*, 1820-1829.

13. Pan, C.; Liu, M.; Zhao, L.; Wu, H.; Ding, J.; Cheng, J., Palladium chloride catalyzed Hiyama cross-coupling reaction using phenyltrimethoxysilane. *Catal. Commun.* **2008,** *9*, 1685-1687.

14. Ando, S.; Hirota, Y.; Matsunaga, H.; Ishizuka, T., Nickel-catalyzed N-arylation of amines with arylboronic acids under open air. *Tetrahedron Lett.* **2019,** *60*, 1277-1280.

15. Nasseri, M. A.; Rezazadeh, Z.; Kazemnejadi, M.; Allahresani, A., A Co–Cu bimetallic magnetic nanocatalyst with synergistic and bifunctional performance for the base-free Suzuki, Sonogashira, and C–N cross-coupling reactions in water. *Dalton trans.* **2020,** *49*, 10645-10660.

16. Sahoo, A. K.; Oda, T.; Nakao, Y.; Hiyama, T., Cross-Coupling of Triallyl (aryl) silanes with Aryl Bromides and Chlorides: An Alternative Convenient Biaryl Synthesis. *Adv. Synth. Catal.* **2004,** *346*, 1715-1727.

17. Tong, X.; Luo, S.-S.; Shen, H.; Zhang, S.; Cao, T.; Luo, Y.-P.; Huang, L.-L.; Ma, X.-T.; Liu, X.-W., Nickel-catalyzed defluorinative alkylation of C (sp^2)–F bonds. *Org. Chem. Front.* **2021,** *8*, 4533-4542.
18. Zhang, J.; Zhang, W.; Wang, Y.; Zhang, M., Palladium-Iminodiacetic Acid Immobilized on pH-Responsive Polymeric Microspheres: Efficient Quasi-Homogeneous Catalyst for Suzuki and Heck Reactions in Aqueous Solution. *Adv. Synth. Catal.* **2008,** *350*, 2065-2076.
19. Sheikh, S.; Nasseri, M. A.; Chahkandi, M.; Reiser, O.; Allahresani, A., Dendritic structured palladium complexes: magnetically retrievable, highly efficient heterogeneous nanocatalyst for Suzuki and Heck cross-coupling reactions. *RSC adv.* **2022,** *12*, 8833-8840.
20. Bencivenni, G.; Lanza, T.; Leardini, R.; Minozzi, M.; Nanni, D.; Spagnolo, P.; Zanardi, G., Iminyl radicals from α-Azido o-Iodoanilides via 1, 5-H transfer reactions of Aryl radicals: new transformation of α-azido acids to decarboxylated nitriles. *J. Org. Chem.* **2008,** *73*, 4721-4724.
21. Zuo, B.; Chen, J.; Liu, M.; Ding, J.; Wu, H.; Su, W., Scandium triflate-catalysed synthesis of N-substituted pyrroles from amine and 2, 5-dimethoxytetrahydrofuran. *J. Chem. Res.* **2009,** *2009*, 14-16.
22. Sun, W.-B.; Zhang, P.-Z.; Jiang, T.; Li, C.-K.; An, L.-T.; Shoberu, A.; Zou, J.-P., CoPc/Cu (OAc) 2-catalyzed N-arylation of amines with arylhydrazines leading to N-aryl amines. *Tetrahedron.* **2016,** *72*, 6477-6483.
